# Chromene-based BioAIEgens: ‘in-water’ synthesis, regiostructure-dependent fluorescence and ER-specific imaging

**DOI:** 10.1093/nsr/nwad233

**Published:** 2023-09-04

**Authors:** Xu-Min Cai, Yuting Lin, Jianyu Zhang, Ying Li, Zhenguo Tang, Xuedan Zhang, Ying Jia, Wenjin Wang, Shenlin Huang, Parvej Alam, Zheng Zhao, Ben Zhong Tang

**Affiliations:** Jiangsu Co-Innovation Center of Efficient Processing and Utilization of Forest Resources, International Innovation Center for Forest Chemicals and Materials, College of Chemical Engineering, Nanjing Forestry University, Nanjing 210037, China; School of Science and Engineering, Shenzhen Institute of Aggregate Science and Technology, The Chinese University of Hong Kong, Shenzhen (CUHK-Shenzhen), Shenzhen 518172, China; Jiangsu Co-Innovation Center of Efficient Processing and Utilization of Forest Resources, International Innovation Center for Forest Chemicals and Materials, College of Chemical Engineering, Nanjing Forestry University, Nanjing 210037, China; Department of Chemistry, Hong Kong Branch of Chinese National Engineering Research Center for Tissue Restoration and Reconstruction, The Hong Kong University of Science and Technology, Hong Kong 999077, China; School of Science and Engineering, Shenzhen Institute of Aggregate Science and Technology, The Chinese University of Hong Kong, Shenzhen (CUHK-Shenzhen), Shenzhen 518172, China; Jiangsu Co-Innovation Center of Efficient Processing and Utilization of Forest Resources, International Innovation Center for Forest Chemicals and Materials, College of Chemical Engineering, Nanjing Forestry University, Nanjing 210037, China; Jiangsu Co-Innovation Center of Efficient Processing and Utilization of Forest Resources, International Innovation Center for Forest Chemicals and Materials, College of Chemical Engineering, Nanjing Forestry University, Nanjing 210037, China; Jiangsu Co-Innovation Center of Efficient Processing and Utilization of Forest Resources, International Innovation Center for Forest Chemicals and Materials, College of Chemical Engineering, Nanjing Forestry University, Nanjing 210037, China; School of Science and Engineering, Shenzhen Institute of Aggregate Science and Technology, The Chinese University of Hong Kong, Shenzhen (CUHK-Shenzhen), Shenzhen 518172, China; Jiangsu Co-Innovation Center of Efficient Processing and Utilization of Forest Resources, International Innovation Center for Forest Chemicals and Materials, College of Chemical Engineering, Nanjing Forestry University, Nanjing 210037, China; Clinical Translational Research Center of Aggregation-Induced Emission, School of Medicine, The Second Affiliated Hospital, School of Science and Engineering, The Chinese University of Hong Kong, Shenzhen (CUHK-Shenzhen), Shenzhen 518172, China; School of Science and Engineering, Shenzhen Institute of Aggregate Science and Technology, The Chinese University of Hong Kong, Shenzhen (CUHK-Shenzhen), Shenzhen 518172, China; School of Science and Engineering, Shenzhen Institute of Aggregate Science and Technology, The Chinese University of Hong Kong, Shenzhen (CUHK-Shenzhen), Shenzhen 518172, China

**Keywords:** chromene, BioAIE, ‘in-water’ chemistry, regiostructure dependence, ER-specific imaging

## Abstract

Exploration of artificial aggregation-induced emission luminogens (AIEgens) has garnered extensive interest in the past two decades. In particular, AIEgens possessing natural characteristics (BioAIEgens) have received more attention recently due to the advantages of biocompatibility, sustainability and renewability. However, the extremely limited number of BioAIEgens extracted from natural sources have retarded their development. Herein, a new class of BioAIEgens based on the natural scaffold of chromene have been facilely synthesized via green reactions in a water system. These compounds show regiostructure-, polymorphism- and substituent-dependent fluorescence, which clearly illustrates the close relationship between the macroscopic properties and hierarchical structure of aggregates. Due to the superior biocompatibility of the natural scaffold, chromene-based BioAIEgens can specifically target the endoplasmic reticulum (ER) via the introduction of tosyl amide. This work has provided a new chromene scaffold for functional BioAIEgens on the basis of green and sustainable ‘in-water’ synthesis, applicable regiostructure-dependent fluorescence, and effective ER-specific imaging.

## Teaser

“In water” synthesis was conducted to produce a new class of natural chromene-based BioAIEgens with regiostructure-, polymorphism- and substituent-dependent fluorescence and ER-specific imaging.

## INTRODUCTION

**Figure 1.**
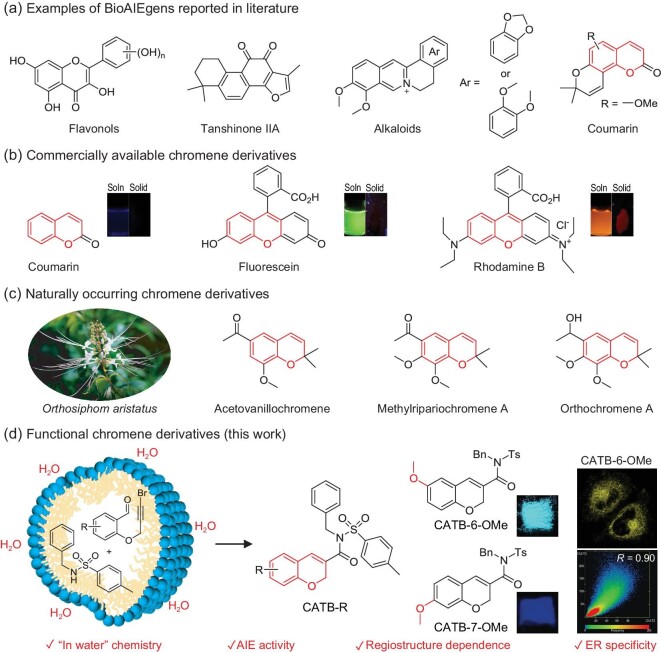
BioAIEgens reported in literature and chromene derivatives. (a) Examples of BioAIEgens reported in literature. (b) Commercially available chromene derivatives and their fluorescence images in solution (soln) and solid states. The structure highlighted in red represents chromene. (c) An image of the herb *Orthosiphom aristatus* and the chemical structures of the naturally occurring chromene derivatives extracted from *O. aristatus* (*O. aristatus* modified from an online image). (d) Synthesis and properties of functional chromene-based BioAIEgens in this work.

Natural products have always played a crucial role in human development and have been studied extensively in order to comprehend their structure–property relationships. These investigations have benefited from the fast growth of organic chemistry [1–4], and medicinal applications for human health have been mainly targeted [3,4]. For instance, Chinese herbs have been used for thousands of years but remain controversial because of their unclear functional route; clarifying this route is a task for the modern pharmaceutical industry. Hence, studying natural products may provide a new method for tracking their functional mechanisms and metabolic processes in living organisms. In recent years, besides their medicinal applications, some natural products have been found to have fluorescent properties, enriching their multifunctionality and attracting a lot of interest [1,2]. Thorough investigations demonstrate that the restriction of intramolecular motion is one main mechanism accounting for the emission in the aggregate state, laying the basic fluorescence foundation for aggregation-induced emission (AIE) luminogens (AIEgens) [5–7]. Although a large amount of artificial AIEgens have been reported and applied in the areas of imaging [8], theragnostics [9], organic light-emitting diodes [10], etc. during the last 20 years, many of them inevitably encountered disadvantages in biocompatibility, sustainability and renewability. Recently, a dozen AIEgens either possessing natural scaffolds or derived from natural resources (BioAIEgens) have been developed for biological diagnosis and treatment (Fig. 1a) [11–19]. For example, flavanols can be used for bioimaging [11,12]. In addition, both alkaloids and tanshinone IIA are not only useful for imaging but are also used to kill cancer cells and bacteria via photodynamic therapy [13–16]. Recently, natural coumarin-based isomers with dramatically distinct AIE properties have been verified with successful wash-free mitochondria imaging [17]. However, these pure natural BioAIEgens still need to be improved due to a lack of scaffold variety and complex extraction. Therefore, exploring BioAIEgens with new structural scaffolds through chemical modifications might be a good alternative to pure extracted BioAIEgens.

Coumarin is a widespread natural product possessing a universal chromene scaffold that is analogous to coumarin and chromone (Supplementary Fig. S1) [20]. Due to its blue-violet emission [21], coumarin cannot be used in practical applications. The recent discovery of natural coumarin-derivatized BioAIEgens confirms the feasibility of the derivatization method for further applications (*vide supra*). With chemical modifications, the chromene scaffold can be transformed into either fluorescein or rhodamine B, which are commercially available as fluorescent markers (Fig. 1b). However, these compounds undergo a destructive aggregation-caused quenching (ACQ) effect [22,23]. From a pharmaceutical point of view, chromene scaffolds are present in various natural products, such as acetovanillochromene, methylripariochromene A and orthochromene A, extracted from the herb *Orthosiphom aristatus* (Fig. 1c), which can be used as a traditional medicine for diabetes and hypertension [20,24], hence the molecular design endowing the bioactive chromene scaffold with an AIE property, making it a win-win strategy with regard to the functionalization of the chromene core.

In general, most chemical reactions are carried out in organic solvents, which require additional engineering procedures accompanied by toxic and environmentally unfriendly organic waste. In fact, natural products are biosynthesized by living organisms in the aqueous environment [25]. As a result, the synthesis of functional chromene derivatives in water will mean that the synthetic route has a green and sustainable impact. One option is to conduct reactions in aqueous micellar systems, i.e. in homogeneously distributed self-assembled nanoreactors formed by amphiphilic agents in water, such as surfactants [26,27]. Thus, the chemical modifications within a micellar system in water will endow the resultant chromene-based BioAIEgens with green and sustainable characteristics besides their functions.

So far, numerous fluorescent dyes have been obtained by chemical modification based on molecular design, but they do not always exhibit corresponding properties in the macroscopic state [28,29]. More studies also back up the fact that aggregate structures could affect macroscopic properties to a large extent [30–32]. Nevertheless, it is challenging to illustrate the relationships between aggregate structures and macroscopic properties. For instance, substituent-dependent fluorescence is often utilized to understand structure-property relationships [18,22,33,34], but fluorescence with regiostructural variations is less targeted, especially at the aggregate level [35,36]. Therefore, explicitly demonstrating the regiostructure–property relationship between aggregate structures and macroscopic behaviors is challenging but considerably significant for molecular design.

In this work, a series of BioAIEgens (CATB-R) based on the natural scaffold of chromene were constructed in the environmentally friendly solvent of water, and rationally incorporated the endoplasmic reticulum (ER) targeted group of tosyl amide and regiostructure-dependent substituents (Fig. 1d). The regiodependent substituent has exhibited the completely uniform tendency of the six-substituted products possessing stronger and red-shifted emissions compared to the seven-substituted ones, suggesting the successful regulation of macroscopic properties via the tuning of regiostructures at molecular level. In addition, the polymorphism-dependent behaviors of CATB-6-OMe also verify the importance of aggregate structures in their macroscopic performance. As a proof-of-concept, these BioAIEgens can be specifically utilized to visualize the ER organelle, attributed to the superior biocompatibility of the natural chromene scaffold and the targeting moiety of tosyl amide. This work introduces an ‘in-water’ method to synthesize chromene-based BioAIEgens and paves the way to molecular design based on regiostructure–property relationships. Benefiting from both the outstanding bioimaging performance and facile regiostructural substitution on the chromene scaffold, these BioAIEgens exhibit great potential in theragnostic applications.

## RESULTS AND DISCUSSION

### ‘In-water’ synthesis of chromene derivatives and their AIE properties

**Figure 2.**
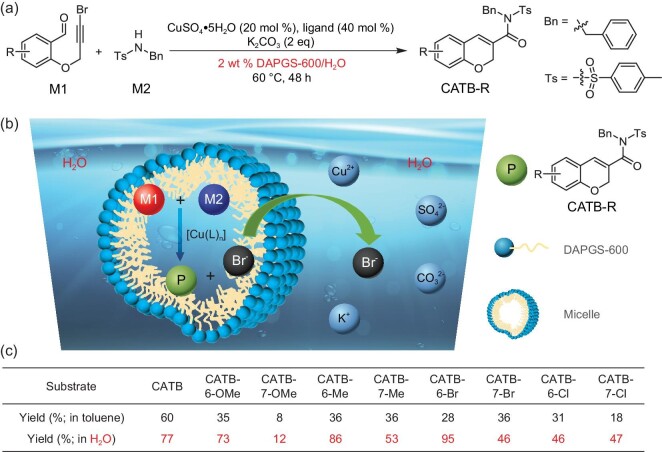
‘In-water’ synthesis of chromene derivatives. (a) Synthetic route to chromene derivatives in water. (b) Schematic process for the synthesis of chromene derivatives in water. (c) Isolation yields of chromene derivatives either in toluene or in water.

Inspired by the biosynthesis of natural products in the aqueous environment, we have applied this chromene-targeted ring-closing metathesis reaction in an aqueous micellar system. The optimization of the reaction conditions indicates that reactants of M1 and M2 undergo the reaction in a 2 wt% DAPGS-600/H_2_O micellar system [37,38] at 60°C for 48 h catalyzed by cheap copper salt and corresponding reagents (Fig. 2a, Supplementary Table S1). Compared to the same reaction in toluene [39], these regiosubstituted chromene derivatives synthesized in the micellar system show higher isolation yields (Fig. 2c). Figure 2b shows a schematic illustration of the reaction procedure. Firstly, due to their hydrophobic characteristic, M1, M2 and the copper-based catalyst ([Cu(L)_n_]) prefer to enter the surfactant-constituted micelle, where the hydrophobic rosin moiety levels are favorable to dissolve them. Then, their collision rate is improved due to the confinement effect of the micelle, which increases the concentration of reactants. In addition, the side product of Br^–^ can pass through the micelle membrane to water easily due to its obvious hydrophilic nature, therefore accelerating the reaction. The above-mentioned positive effects clearly illustrate higher yields than those in toluene. Besides, the surfactant of DAPGS-600 can give rise to the best yield compared to SDBS, TPGS-750-M [40] and APGS-550-M (Supplementary Table S1), most probably because the benzene-ring-incorporated rosin moiety inside the micelle is more miscible to aromatic species. In addition to the regiostructure-dependent products, a nitrogen-containing heterocyclic product (QATB-N-Ts) structurally analogous to chromene can also be obtained according to the identical synthetic route (Supplementary Fig. S2). This creative synthetic method not only verifies the synthetic feasibility of the targeted chromene structure in water but also means that the reaction is green and sustainable.

Details of the synthesis of surfactants, the optimization of reaction conditions, the gram-scale reaction and other synthetic protocols can be found in the Supplementary Data. The structures and purity of the obtained products were confirmed by nuclear magnetic resonance (NMR) and high-resolution mass spectroscopic (HRMS) measurements (please see NMR and HRMS spectra in the Supplementary Data). Single crystals of polymorphic CATB-6-OMe (C_b_ and C_g_) and CATB-6-Me suitable for X-ray diffraction measurement could be obtained via slow evaporation in their respective solutions (please see details in the Supplementary Data).

To verify the regiostructure-dependent photophysical properties of the chromene-based BioAIEgens, their photoluminescence (PL) spectra were measured in acetonitrile/water (ACN/H_2_O) mixtures (Supplementary Figs S3–S15). The chromene derivatives possess a propeller conformation that is centralized on the nitrogen atom from a structural point of view, which may endow them with AIE properties. As expected, the non-substituted product of CATB shows an opposite PL change to fluorescein and rhodamine B with ACQ behavior, realizing the activation of the AIE property on the chromene scaffold. In addition, CATB exhibits a longer-wavelength emission than coumarin, despite its non-conjugated structure. For the methoxy-group-substitued CATB-6-OMe, the PL intensity first decreases with the increased water fraction (*f*_w_) and then immediately increases when *f*_w_ is over 70%, whereas the change of wavelength goes the opposite way, suggesting a typical twisted intramolecular charge transfer (TICT) and AIE behavior (Supplementary Fig. S6). The nitrogen-containing analogue of QATB-N-Ts also displays similar TICT-AIE properties, verified by its PL spectra (Supplementary Fig. S15). Regarding other chromene-based products, they all exhibit gradually enhanced PL intensity with an increase in *f*_w_ due to their hydrophobic nature. In essence, the obtained chromene derivatives are all AIE-active but vary in intensity and wavelength, with structural variations.

### Fluorescence and proposed structures in the excited state

**Figure 3.**
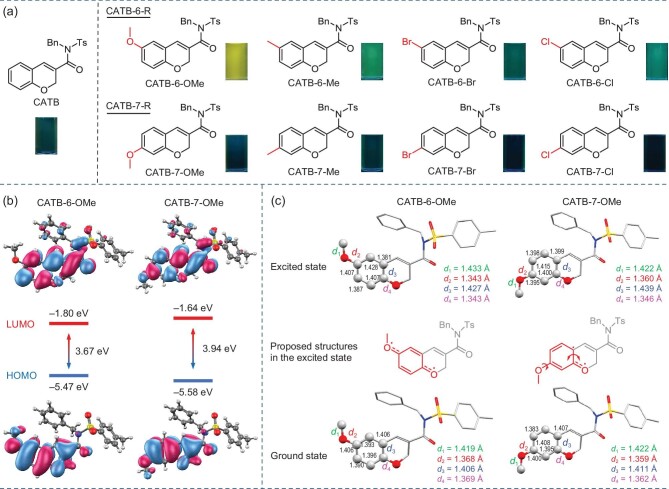
Fluorescence and proposed structures in the excited state. (a) Chemical structures and fluorescent images of six-/seven-substituted chromene-based BioAIEgens in acetonitrile solutions (20 μM) under a 365 nm UV lamp. (b) Frontier molecular orbitals and corresponding energy levels of CATB-6-OMe and CATB-7-OMe based on their optimized ground-state geometries. (c) Calculated geometries of CATB-6-OMe and CATB-7-OMe in the ground and excited states, as well as their proposed structures in the excited state with the quinone part highlighted in red.

After analyzing the PL properties of all products, an interesting phenomenon is observed. It appears that six-substituted products possess stronger and red-shifted emissions at the molecular level compared to those of seven-substituted products that have similar emission properties to the non-substituted CATB (Fig. 3a, Supplementary Fig. S16). To shed more light on the special fluorescence properties, density functional theory (DFT) calculations were carried out. As shown in Fig. 3b and Supplementary Figs S17–S21, CATB shows a band gap of 4.10 eV, which is close to that of seven-substituted products but larger than six-substituted ones, indicating that their emission color is similar to that of seven-substituted products but more blue-shifted than six-substituted ones. Further, the six-substituted products show a stronger charge transfer (CT) effect. Further, the six-substituted products show a stronger charge transfer (CT) effect than the seven-substituted ones, suggesting their redder emission. Their band gaps and electron-hole analysis also verify the above conclusion. Overall, CATB-6-OMe exhibits the strongest CT effect, which is in good accordance with its TICT behavior. Apart from emission wavelength, the stronger emission intensity of the six-substituted products has given rise to another curiosity. According to previously reported work [18], emission intensity is highly dependent on the molecular motion in the excited state. In other words, the more active the molecular motion in the excited state is, the weaker the emission turns out to be. Hence, the excited-state geometries of each pair of regioisomers have been optimized for analysis using the time-dependent DFT method (Fig. 3c, Supplementary Figs S18–S20). For CATB-6-OMe, the two O−C bond lengths (*d*_2_ = 1.343 and *d*_4_ = 1.343 Å) in the excited state are largely shorter than those in the ground state, whereas the lengths of *d*_1_ (1.433 Å) and *d*_3_ (1.427 Å) become larger, indicating its quasi quinone structure, which can rigidify the molecular motion in the excited state. For CATB-7-OMe, the lengths of *d*_1_ and *d*_2_ are similar in both excited and ground states. In contrast, *d*_3_ in the excited state (1.439 Å) is larger than in the ground state (1.411 Å), while *d*_4_ has the opposite trend. The above results suggest a semi-quasi-quinone structure with the methoxy characteristic maintained but the C=C bond (*d*_3_) elongated. Such a semi-quasi-quinone structure can provide CATB-7-OMe with more active molecular motion in the excited state, resulting in much weaker emissions than those of CATB-6-OMe. Similar structural differences can be found for other pairs of regioisomers, suggesting that all seven-substituted products have a clear tendency towards stronger molecular motion and weaker emissions.

### Regiostructure-dependent fluorescence at the varied aggregate level

**Figure 4.**
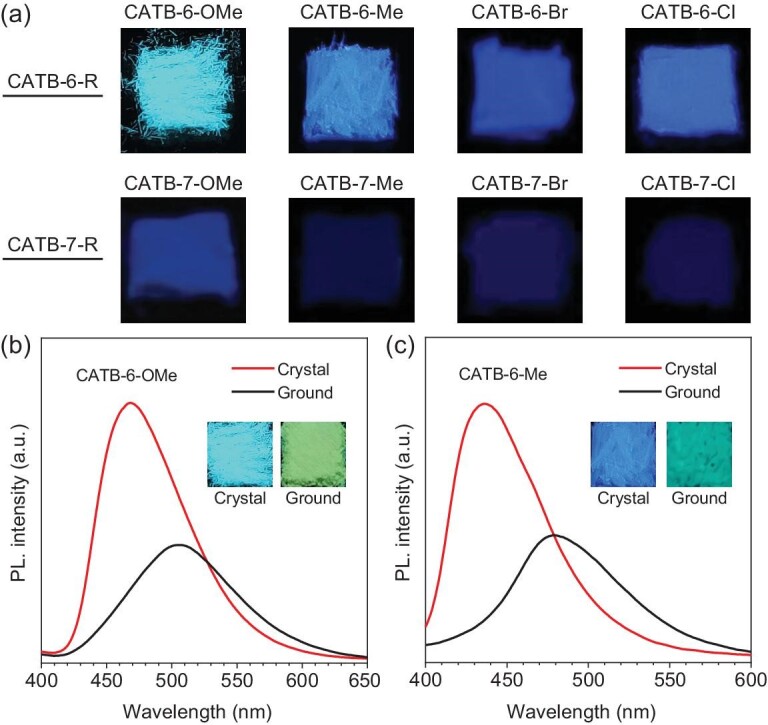
Regiostructure-dependent fluorescence at varied aggregate level. (a) Fluorescence images of crystalline six-/seven-substituted chromene-based BioAIEgens under a 365 nm UV lamp. (b and c) PL spectra of crystalline CATB-6-OMe (b) and CATB-6-Me (c) before and after grinding. CATB-6-OMe: *λ*_ex_: 365 nm; CATB-6-Me: *λ*_ex_: 355 nm.

As demonstrated by fluorescein and rhodamine B, commercial chromene derivatives display fluorescence quenching in the solid state. Therefore, the solid-state photophysical properties of the newly synthesized chromene derivatives were further investigated. As a result, a similar trend is observed in the solid state; the six-substituted products produce longer-wavelength emissions and stronger intensity than the seven-substituted ones (Fig. 4a, Supplementary Fig. S22), indicating the effective regulation of aggregate-state properties by regiostructural design at the molecular level. However, both the solid-state six- and seven-substituted products respectively present blue-shifted emissions compared to the molecular level (Supplementary Fig. S23), suggesting the essential role of hierarchical structures in their photophysical properties. The crystals of CATB-6-OMe exhibit strong sky-blue emissions, while much weaker and red-shifted emissions are observed after grinding (Fig. 4b), which is closely related to the crystallization-induced emission (CIE) effect [41]. More specifically, the crystals go through the Frank-Condon channel with the least non-radiative decay. After grinding, the crystals are probably destructed to form amorphous states on the crystalline surfaces (Supplementary Fig. S24), destroying multiple intermolecular interactions and resulting in more active intramolecular motion. As a result, CATB-6-OMe relaxes to an excited-state geometry with a smaller band gap and enhanced non-radiative decay, leading to red-shifted and weakened emissions. The similar phenomenon of CATB-6-Me also supports the above conclusion (Fig. 4c). In addition, the stronger emissions of six-substituted products also indicate that they intrinsically possess more intermolecular interactions in the solid state than seven-substituted products. Accordingly, they are more inclined to form single crystals (only single crystals of CATB-6-OMe and CATB-6-Me are obtained).

### Polymorphism-dependent fluorescence with distinct aggregate structures

**Figure 5.**
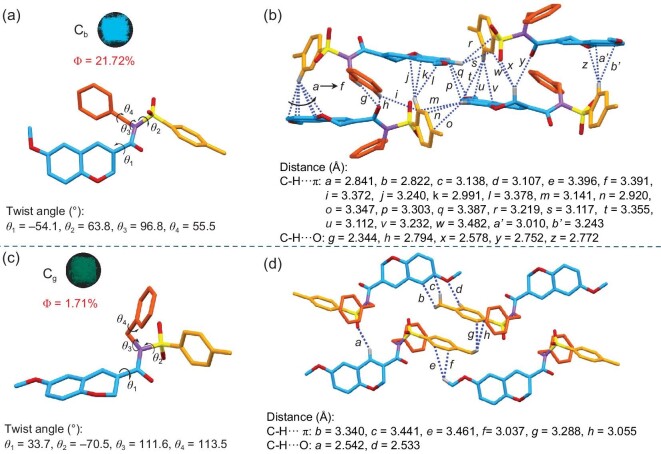
Polymorphism-dependent fluorescence with distinct aggregate structures. (a and c) Fluorescence images taken under a 365 nm UV lamp. Quantum yield (Φ) and molecular conformations for C_b_ (a) and C_g_ (c) of CATB-6-OMe. (b and d) Intermolecular C-H···π and C-H···O interactions for C_b_ (b) and C_g_ (d) of CATB-6-OMe.

Although some results discussed above indicate the differences in photophysical properties between the crystalline and ground samples, it is not easy to clearly illustrate the structure-property relationships in the aggregate state due to the vague aggregated structure of the ground samples. Fortunately, two single-crystal structures (C_b_ and C_g_) of CATB-6-OMe with distinct luminescent properties are obtained, providing an excellent platform to understand their polymorph-dependent properties (Fig. 5, Supplementary Fig. S25 and Supplementary Table S2). The sky-blue emissive crystals (C_b_) show a propeller conformation with torsion angles of *θ*_1_ = –54.1°, *θ*_2_ = 63.8°, *θ*_3_ = 96.8° and *θ*_4_ = 55.5°, respectively. Such twisted conformation gives rise to intensive rigidifications with C-H···π and C-H···O interactions, restricting their molecular motions in the excited state and producing a quantum yield of 21.72% in the solid state. Interestingly, when the benzyl moiety rotates to the tosyl group, a green emissive crystal (C_g_) with a dramatic torsion angle change (*θ*_1_ = –54.1°, *θ*_2_ = 63.8°, *θ*_3_ = 96.8° and *θ*_4_ = 55.5°) can be obtained, affording another conformation with distinct spatial orientation. This unique conformation has unexpectedly led to fewer intermolecular C-H···π and C-H···O interactions, resulting in comparatively strong molecular motion with a lower quantum yield of 1.71%. Coincidently, C_g_ shows a red-shifted emission, probably due to the more relaxed geometry with a smaller band gap. For CATB-6-Me, a similar spatial orientation to C_b_ can be found with torsion angles of *θ*_1_ = –49.1°, *θ*_2_ = 55.1°, *θ*_3_ = 108.3° and *θ*_4_ = 166.4°, respectively. Thus, the distinct aggregate structure of CATB-6-Me with a medium amount of intermolecular C-H···π and C-H···O interactions gives rise to the blue-shifted emission with a moderate quantum yield of 13.93% (Supplementary Fig. S26). The more prominent fluorescent distinctions between polymorphic CATB-6-OMe (C_b_ and C_g_) over the variable-substituted CATB-6-Me further affirm that the solid-state photophysical property is highly dependent on the aggregate structures.

### ER-specific imaging

**Figure 6.**
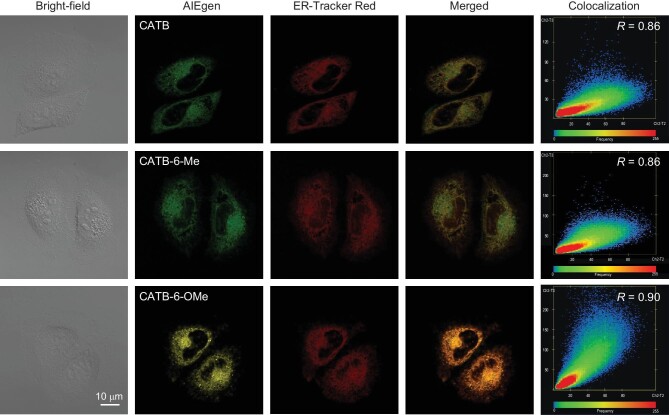
ER-specific imaging. Fluorescence images of A549 cells co-stained with 10 μM of CATB, CATB-6-Me, CATB-6-OMe, and 1 μM of ER-Tracker Red for 30 min, respectively. CATB, CATB-6-Me, CATB-6-OMe: *λ*_ex_: 405 nm, *λ*_em_: 450–600 nm; ER-Tracker Red: *λ*_ex_: 561 nm, *λ*_em_: 600–700 nm. *R* = Pearson's correlation coefficient.

The ER is the largest organelle in eukaryotic cells. It plays a significant role in bodily functions and is responsible for protein synthesis and transport, lipid metabolism and calcium storage [42]. A dysfunction of the ER can lead to serious diseases like Alzheimer's disease, prion disease and other cardiovascular diseases [43–45]. Hence, it is important to have proper imaging of the ER to understand the progression of disease in living organisms. So far, imaging sensors for the ER organelle are limited [43,46], far behind the progress of other organelles like mitochondria and lysosomes [47–49], resulting in a demand for the development of effective ER-specific sensors. Previous research suggested the targeting ability of sulfonamide on the ER organelle [43,50]. This was adopted in the initial molecular design by introducing tosyl amide in our synthetic route. Besides, chromene as a natural product may bring intrinsic biocompatibility, endowing the resultant BioAIEgens with very low toxicity. To verify the feasibility of ER-specific imaging based on the chromene scaffold, we first evaluated cytotoxicity, employing both 3-(4,5-dimethyl-2-thiazolyl)-2,5-diphenyl-2-H-tetrazolium bromide (MTT) and live-dead cell staining experiments. In the case of A549 cells, it has been observed that up to 98% viability is maintained even at a high concentration of 20 μM (Supplementary Fig. S27a), indicating that these chromene-based BioAIEgens have low toxicity and do not cause a disturbance to cellular metabolism during ER imaging processes. The above results are further confirmed by the photo-killing process of the concentration-dependent live-dead cell staining test (Supplementary Figs S28–S31). Due to their outstanding compatibility, CATB, CATB-6-Me and CATB-6-Cl are applied in cell imaging, and efficiently stain ER with Pearson's correlation coefficients (*R*) of 0.86 and 0.85, respectively (Fig. 6, Supplementary Fig. S32). When the substituent is replaced to a methoxy group, CATB-6-OMe can achieve brighter imaging and a more superior colocalization effect with a higher *R* value (up to 0.90), suggesting the improvement of imaging via substituent variation. Regarding HeLa cells, their cell imaging exhibits an *R* coefficient from 0.84 to 0.88 (Supplementary Fig. S33). CATB-6-OMe also shows the highest *R* coefficient and the best biocompatibility, with cell viability of ∼96% under a concentration of 20 μM (Supplementary Fig. S27b). The above results confirm the superior performance of the natural scaffold and highlight the possibility that both the cell viability and imaging behaviors of these BioAIEgens can be readily regulated by varying the substituents on the chromene scaffold, providing more possibilities to explore outstanding BioAIEgens based on this system.

## CONCLUSION

Natural BioAIEgens with unique structural scaffolds extracted from bioresources are significant types of bioactive materials that have the advantages of natural availability, renewability, sustainability and biocompatibility compared with artificial AIEgens. However, the limited variety of BioAIEgens that are currently available has hindered their practical application. As a versatile natural scaffold, chromene can be transformed into commercial dyes such as fluorescein and rhodamine B, which exhibit ACQ behaviors in the aggregate state. To overcome the ACQ limitations of existing chromene derivatives, we have successfully synthesized a new class of chromene-based BioAIEgens in a unique aqueous micellar system with higher yields than toluene, endowing the reaction with green and sustainable characteristics. Surprisingly, a regiostructure-dependent fluorescence property is observed in that the six-substituted series show significantly stronger fluorescence at longer wavelengths than the seven-substituted series. Furthermore, the polymorphism-dependent fluorescence distinctions of CATB-6-OMe (C_b_ and C_g_) have confirmed the close relationship between the solid-state photophysical property and the hierarchical structure of aggregates. Taking advantage of the superior biocompatibility endowed by the natural scaffold, the synthesized BioAIEgens are successfully utilized in order to visualize ER organelles. Therefore, this work not only provides a unique aqueous synthetic route to obtaining chromene-based BioAIEgens, but also endows them with tunable fluorescence and imaging abilities via substitution- and regiostructure-dependent variations.

## METHODS AND MATERIALS

The experimental details are given in the online Supplementary Data.

## Supplementary Material

nwad233_Supplemental_Files

## Data Availability

The X-ray crystallographic coordinates for the structures reported in this article have been deposited at the Cambridge Crystallographic Data Centre (CCDC) under deposition numbers CCDC 2084308 (C_b_ of CATB-6-OMe), 2084311 (C_g_ of CATB-6-OMe) and 2084312 (CATB-6-Me). These data can be obtained free of charge from the Cambridge Crystallographic Data Centre via www.ccdc.cam.ac.uk/data_request/cif.
