# Supplementary material for: Chromene-based BioAIEgens: ‘in-water’ synthesis, regiostructure-dependent fluorescence and ER-specific imaging
Source: Natl Sci Rev. 2023 Sep 4;10(11):nwad233. doi: 10.1093/nsr/nwad233 (PMC10769509; doi:10.1093/nsr/nwad233)
Supplement: nwad233_Supplemental_Files [file nwad233_supplemental_files.zip › Revised Supplementary data_NSR_MS-2023-295.docx]

**Supplementary data**

**Chromene-based BioAIEgens: “in water” synthesis, regiostructure-dependent fluorescence and ER-specific imaging**

Xu-Min Cai^1,2,†,*^ , Yuting Lin^1,†^, Jianyu Zhang^3,†^, Ying Li^2^, Zhenguo Tang^1^, Xuedan Zhang^1^, Ying Jia^1^, Wenjin Wang^2^, Shenlin Huang^1^^,^*, Parvej Alam^4^, Zheng Zhao^2,^* and Ben Zhong Tang^2,^*

^1^ Jiangsu Co-Innovation Center of Efficient Processing and Utilization of Forest Resources, International Innovation Center for Forest Chemicals and Materials, College of Chemical Engineering, Nanjing Forestry University, Nanjing 210037, China

^2^ School of Science and Engineering, Shenzhen Institute of Aggregate Science and Technology, The Chinese University of Hong Kong, Shenzhen (CUHK-Shenzhen), Guangdong 518172, China

^3^ Department of Chemistry, Hong Kong Branch of Chinese National Engineering Research Center for Tissue Restoration and Reconstruction, The Hong Kong University of Science and Technology, Clear Water Bay, Kowloon, Hong Kong 999077, China

^4^ Clinical Translational Research Center of Aggregation-Induced Emission, School of Medicine, The Second Affiliated Hospital, School of Science and Engineering, The Chinese University of Hong Kong, Shenzhen (CUHK-Shenzhen), Guangdong 518172, China.

***Corresponding authors.**

E-mails:

[xumin.cai@njfu.edu.cn](mailto:xumin.cai@njfu.edu.cn);

[shuang@njfu.edu.cn](mailto:shuang@njfu.edu.cn);

[zhaozheng@cuhk.edu.cn](mailto:zhaozheng@cuhk.edu.cn);

[tangbenz@cuhk.edu.cn](mailto:tangbenz@cuhk.edu.cn)

†Equally contributed to this work

**This PDF file includes:**

Supplementary experimental procedures

Supplementary Figures S1 to S63, Supplementary Table S1 to S19

**1. Supplementary experimental procedures**

**1.1 Materials availability**

The surfactant precursor, dehydroabietic alcohol succinate, was synthesized according to previously reported procedures in the literature.^1^ Different alkynyl bromides M1 were synthesized according to the literature procedures.^2^ M2 was purchased from Aladdin (≥ 98.0%) and used as received. A 2 wt% DAPGS-600/H2O solution was prepared by dissolving DAPGS-600 in water. All reagents and solvents were used as received from commercial suppliers unless otherwise indicated. Reactions were monitored using thin layer chromatography (TLC) on Merck silica gel plates (GF-254) using UV light at 254 or 365 nm as the visualizing agent. Milli-Q water was from a Milli-Q purification system (Merck Millipore, Germany). Phosphate-buffered saline (PBS) and dulbecco’s minimum essential medium (DMEM) were purchased from Gibco. Fetal bovine serum (FBS), penicillin, and streptomycin were from Invitrogen. ER-Tracker Red was purchased from Beyoyime Biotechnology (China). Calcein AM/PI was purchased from KeyGEN BioTECH (China). A549 cells and HeLa cells were from ATCC.

**1.2 Characteristic**

^1^H NMR and ^13^C NMR spectra were recorded on a Bruker AVANCE-III-600 (^1^H, 600 Hz; ^13^C, 150 Hz) and AVANCE NEO-400 (^1^H, 400 Hz; ^13^C, 100 Hz) instruments internally referenced to CDCl_3_ signals. High-resolution mass spectra (HRMS) were acquired by Agilent 6500 QTOFMS (ESI) and Q Exactive (ESI). Powder X-ray diffraction (PXRD) patterns were collected on a Rigaku Ultima IV diffractometer with Cu Kα radiation (scan range: 5-45°). UV-Vis absorption spectra were obtained with a Shimadzu UV2450 spectrometer. Photoluminescence (PL) spectra were measured by a Fluoromax-4 spectrometer. The absolute fluorescence quantum yields were measured by a Fluoromax-4 spectrometer with a Quanta-ϕ integrating sphere. Confocal laser scanning microscopy (CLSM) images were conducted using an LSM880 (Carl Zeiss, Germany). The ground-state geometries and corresponding frontier molecular orbitals were calculated using the density functional theory (DFT) method at the B3LYP-D3/6-31G(d,p) level. The excited-state geometries and hole-electron analysis were calculated using the time-dependent DFT method at the B3LYP-D3/6-31G(d,p) level. Analytical frequency calculations were also carried out at the same level of theory to confirm the local minimum point of the optimized structures. The above calculations were performed using Gaussian 16 program, and the orbitals were visualized using IQmol program. Single crystal data of CATB-6-OMe (C_b_ and C_g_) and CATB-6-Me were selected and mounted on a SuperNova, Dual, Cu at zero, AtlasS2 diffractometer using Cu Kα radiation (λ = 1.54184 Å) (C_b_ of CATB-6-OMe and CATB-6-Me) and Mo Kα radiation (λ = 0.71073 Å) (C_g_ of CATB-6-OMe). Using Olex2, the structure was solved with the ShelXT structure solution program using Intrinsic Phasing and refined with the ShelXL refinement package using Least Squares minimization.

**1.3 Cell culturing and Cell imaging**

A549 cells and HeLa cells were cultured in DMEM containing 10% FBS and antibiotics (100 units mL^−1^ penicillin and 100 µg mL^−1^ streptomycin) in a 5% CO_2_ humidity incubator at 37°C. Cells were seeded in a confocal dish (5 × 10^4^ cells/mL). After being cultured for 24 h, cells were added with 10 μM of CATB, CATB-6-OMe, CATB-6-Me, CATB-6-Cl, and 1 μM of ER-Tracker Red in culture medium and incubated for 30 min at 37°C. Then, the medium was removed, and the cells were rinsed with PBS (pH = 7.4) three times and then imaged by using CLSM. Capture condition: CATB, CATB-6-OMe, CATB-6-Me, CATB-6-Cl: λ_ex_ = 405 nm, λ_em_ = 450-600 nm; ER-Tracker Red: λ_ex_ = 561 nm, λ_em_ = 600-700 nm.

**1.4 Cytotoxicity assay**

The cell proliferation and cytotoxicity assay were carried out by 3-(4,5-dimethylthiazol-2-yl)-2,5-diphenyltetrazolium bromide (MTT) (Sigma, USA) as follows: A549 and HeLa cell lines were seeded in 96-well plates at a density of 1 × 10^4^ cells per well (200 μL). After 24 h culturing, the medium in each well was replaced by fresh medium containing different concentrations (0, 2, 5, 10, and 20 μM) of CATB, CATB-6-OMe, CATB-6-Me, CATB-6-Cl for 24 h. The medium was removed completely and 100 μL of MTT mixed solution was added to each culture well, which consisted of 90 μL of culture medium and 10 μL of MTT solution (5 mg mL^−1^). After 4 h of incubation at 37°C and in a 5% CO_2_ atmosphere, the 100 μL MTT mixed solution was removed, and then 100 μL of DMSO was added to each well. Finally, OD was measured using an enzyme mark instrument (Tecan, infinite F50, Switzerland), at an optical absorbance of 570 nm. Five replicate measurements were obtained for each sample (n = 5). The survival rate of cells was determined by dividing the cell viability of the cells incubated with CATB, CATB-6-OMe, CATB-6-Me, CATB-6-Cl by the cell viability of the control group performed in the absence of CATB, CATB-6-OMe, CATB-6-Me, CATB-6-Cl.

**1.5 Live-dead cell staining**

A549 cells were seeded and cultured in 96-well plates (1 × 10^4^ cells/200 μL). After 24 h, the culture medium was removed, 100 μL different concentrations of CATB, CATB-6-OMe, CATB-6-Me, CATB-6-Cl (0, 2, 5, 10, 20 μM) were incubated with the cells for another 24 h. The culture medium was removed, and the cells were washed with PBS twice, and 500 μL Calcein AM (2 μM) /PI (8 μM) mixed solution was added into each well and incubated for 20 min. Then, the cells were gently washed with PBS and the imaging was observed by CLSM. Capture condition: Calcein AM: *λ*_ex_: 488 nm, *λ*_em_: 500-550 nm; PI: *λ*_ex_: 561 nm, *λ*_em_: 600-700 nm.

**2. Experimental section**

**2.1 Synthesis of DAPGS-600**

A mixture containing dehydroabietic alcohol succinate (3.3 mmol), polyethylene glycol-600 (3.0 mmol) and *p*-TsOH (0.5 mmol) in toluene (13 mL) was refluxed for 5 h using a Dean-Stark trap. Then the mixture was cooled to RT, and a saturated aqueous NaHCO_3_ solution was added. Extracting with DCM, the combined organic layers were washed with saturated NaHCO_3_ (3 × 50 mL), brine (2 × 30 mL), dried over anhydrous Na_2_SO_4_, and concentrated in vacuo to afford DAPGS-600 as a colorless oil. ^1^H NMR (600 MHz, CDCl_3_), *δ* (ppm): 7.12 (d, *J* = 8.2 Hz, 1H), 6.94 (dd, *J* = 8.1, 1.6 Hz, 1H), 6.84 (d, *J* = 1.1 Hz, 1H), 4.20-4.19 (m, 1H), 4.12-4.10 (m, 2H), 3.94 (d, *J* = 10.9 Hz, 1H), 3.61-3.58 (m, 49H), 2.86-2.75 (m, 3H), 2.61-2.56 (m, 6H), 2.25-2.22 (m, 1H), 1.74-1.70 (m, 2H), 1.69-1.65 (m, 1H), 1.64-1.60 (m, 1H), 1.58-1.56 (m, 1H), 1.39-1.32 (m, 3H), 1.18 (s, 3H), 1.17(d, *J* = 2.3 Hz, 6H), 0.89 (s, 3H). ^13^C NMR (150 MHz, CDCl_3_), *δ* (ppm): 172.2, 172.1, 147.0, 145.5, 134.6, 126.8, 124.2, 123.8, 72.6, 72.5, 70.54, 70.50, 70.2, 69.0, 63.8, 63.7, 61.6, 44.2, 38.2, 37.3, 36.7, 35.4, 33.3, 30.1, 29.1, 29.0, 28.9, 25.3, 23.94, 23.93, 18.9, 18.5, 17.4.

**2.2 Experimental procedures for CATB-R and QATB-N-Ts in water**

Alkynyl bromides (M1: 0.40 mmol), *N*-benzyl-4-methylbenzenesulfonamide (M2: 0.20 mmol), CuSO_4_⋅5H_2_O (0.04 mmol), 4,4'-di-tert-butyl-2,2'-bipyridine (L_2_: 0.08 mmol) and potassium carbonate (0.40 mmol) were added in a 2 wt% DAPGS-600/H_2_O (0.4 mL, 0.5 M) solvent and stirred at 60 ℃ for 48 h. Then the reactions were cooled to RT and extracted with EtOAc. The organic layers were concentrated in vacuo and separated by silica gel column chromatography to obtain the crude products. The crude products were purified by recrystallization from an EtOH solution.

QATB-N-Ts was prepared according to the above synthetic procedures of CATB-R.

**2.3 Optimization of the reaction conditions**

Initially, we used 2-((3-bromoprop-2-yn-1-yl)oxy)benzaldehyde (M1 of CATB) and M2 to the condition^[a]^ to test our hypothesis. The desired product CATB was formed in a 63% yield in 48 h, employing CuSO_4_⋅5H_2_O (20 mol %) as the catalyst, 1,10-phenanthroline (L_1_: 40 mol %) as the ligand, 2 eq of K_2_CO_3_ as the base, and 2 wt % DAPGS-600/H_2_O as the solvent at 60 ℃ (Table S1, Entry 1). After confirming the applicability of the reaction in the aqueous phase, we selected M1 of CATB and M2 as models to explore the ring-closing metathesis to uncover the optimal reaction conditions. When the reaction time was reduced^[c]^ or increased^[d]^, the yield was dramatically decreased to 19% or 30% (Entries 2-3). Under the same reaction conditions, ligand was changed and L_2_ provided a 77% yield for CATB (Entries 4-8). When L_2_ was fixed as ligand, we attempted to replace CuSO_4_⋅5H_2_O with other copper catalysts, such as CuBr_2_, CuCl_2_⋅2H_2_O, and Cu(OAc)_2_. However, much lower yields were obtained (Entries 9-11). After determination of catalyst and ligand, we optimized the amounts^[e-f]^ and found that CuSO_4_⋅5H_2_O (20 mol %) and L_2_ (40 mol %) can give rise to CATB with the highest yield (Entries 12-13). Moreover, inferior reaction performance with decreased yields was also observed when bases like NaOH, K_3_PO_4_ and aqueous systems like TPGS-750-M,^3^ APGS-550-M,^4^ SDBS, and pure water were used (Entries 14-19).

**Table S1.** Optimization of the reaction conditions^[a]^

**Scheme S1.** Ligand scope.

**2.4 Experimental procedures for CATB-R and QATB-N-Ts in toluene^2^**

Alkynyl bromides (0.20 mmol), M2 (0.20 mmol), CuSO_4_⋅5H_2_O (0.04 mmol), 1,10-phenanthroline (L_1_: 0.08 mmol) and potassium carbonate (0.40 mmol) were added in toluene (0.52 mL) and stirred at 70 °C for 48 h. Then the reaction was cooled to RT and extracted with EtOAc. The organic layers were concentrated in vacuo and separated by silica gel column chromatography to obtain the crude products. The crude products were purified by recrystallization from an EtOH solution.

QATB-N-Ts was prepared according to the above synthetic procedures of CATB-R.

**2.5 Gram-scale preparation of CATB**

2-((3-bromoprop-2-yn-1-yl)oxy)benza-ldehyde (M1 of CATB) reacted smoothly with M2 in gram-scale to afford the corresponding product of CATB in 49% yield.

**2.6 Characterization data for all products**

***N*-benzyl-*N*-tosyl-2*H*-chromene-3-carboxamide (CATB)**

White solid, 77% yield. ^1^H NMR (600 MHz, CDCl_3_), *δ* (ppm): 7.65 (d, *J* = 8.1 Hz, 2H), 7.30-7.26 (m, 7H), 7.22 (t, *J* = 7.6 Hz, 1H), 6.98 (d, *J* = 7.3 Hz, 1H), 6.90 (t, *J* = 7.4 Hz, 1H), 6.85 (s, 1H), 6.82 (d, *J* = 8.1 Hz, 1H), 4.91 (s, 2H), 4.78 (s, 2H), 2.44 (s, 3H).

***N*-benzyl-6-methoxy-*N*-tosyl-2*H*-chromene-3-carboxamide (CATB-6-OMe)**

C_b_ (needle-like) was obtained by slow evaporation from an EtOH solution at RT. C_g_ (block-like) was obtained by slow evaporation from an EtOH solution cooled in the refrigerator. 73% yield. ^1^H NMR (600 MHz, CDCl_3_), *δ* (ppm): 7.64 (d, *J* = 8.2 Hz, 2H), 7.32-7.26 (m, 7H), 6.82-6.75 (m, 3H), 6.52 (d, *J* = 2.8 Hz, 1H), 4.90 (s, 2H), 4.71 (s, 2H), 3.76 (s, 3H), 2.44 (s, 3H). ^13^C NMR (150 MHz, CDCl_3_), *δ* (ppm): 169.3, 154.5, 149.1, 145.0, 136.2, 136.0, 133.1, 129.9, 128.9, 128.4, 128.1, 127.5, 121.3, 118.2, 117.1, 113.1, 65.1, 55.9, 50.7, 21.8. HRMS-ESI (m/z) [M+Na]^+^ calcd for C_25_H_23_NNaO_5_S: 472.1189; found: 472.1181.

***N*-benzyl-7-methoxy-*N*-tosyl-2*H*-chromene-3-carboxamide (CATB-7-OMe)**

White solid, 12% yield. ^1^H NMR (600 MHz, CDCl_3_), *δ* (ppm): 7.66 (d, *J* = 8.1 Hz, 2H), 7.32-7.26 (m, 7H), 7.00 (s, 1H), 6.94 (d, *J* = 8.4 Hz, 1H), 6.48 (d, *J* = 9.7 Hz, 1H), 6.38 (s, 1H), 4.83 (s, 2H), 4.78 (s, 2H), 3.78 (s, 3H), 2.44 (s, 3H). ^13^C NMR (150 MHz, CDCl_3_), *δ* (ppm): 169.4, 163.4, 157.0, 144.8, 136.2, 135.9, 134.8, 130.5, 129.8, 128.8, 128.4, 128.3, 128.0, 123.2, 114.0, 108.6, 101.6, 65.2, 55.6, 50.9, 21.8. HRMS-ESI (m/z) [M+Na]^+^ calcd for C_25_H_23_NNaO_5_S: 472.1189; found: 472.1167.

***N*-benzyl-6-methyl-*N*-tosyl-2*H*-chromene-3-carboxamide (CATB-6-Me)**

White solid, 86% yield. Crystal (needle-like) was obtained by slow evaporation from an EtOH solution at RT. ^1^H NMR (600 MHz, CDCl_3_), *δ* (ppm): 7.64 (d, *J* = 8.3 Hz, 2H), 7.31-7.26 (m, 7H), 7.02 (d, *J* = 8.1 Hz, 1H), 6.86 (s, 1H), 6.79 (s, 1H), 6.72 (d, *J* = 8.2 Hz, 1H), 4.90 (s, 2H), 4.74 (s, 2H), 2.44 (s, 3H), 2.25 (s, 3H). ^13^C NMR (150 MHz, CDCl_3_), *δ* (ppm): 169.3, 153.0, 144.9, 136.2, 136.0, 133.5, 132.9, 131.3, 129.8, 129.4, 128.8, 128.4, 128.3, 128.0, 126.6, 120.5, 116.0, 65.1, 50.7, 21.8, 20.6. HRMS-ESI (m/z) [M+Na]^+^ calcd for C_25_H_23_NNaO_4_S: 456.1240; found: 456.1238.

***N*-benzyl-7-methyl-*N*-tosyl-2*H*-chromene-3-carboxamide (CATB-7-Me)**

White solid, 53% yield. ^1^H NMR (600 MHz, CDCl_3_), *δ* (ppm): 7.65 (d, *J* = 8.2 Hz, 2H), 7.31-7.26 (m, 7H), 6.90-6.87 (m, 2H), 6.72 (d, *J* = 7.7 Hz, 1H), 6.64 (s, 1H), 4.88 (s, 2H), 4.75 (s, 2H), 2.44 (s, 3H), 2.30 (s, 3H). ^13^C NMR (150 MHz, CDCl_3_), *δ* (ppm): 169.4, 155.2, 144.9, 143.4, 136.2, 136.0, 133.8, 129.8, 129.1, 128.8, 128.40, 128.38, 128.0, 125.4, 122.9, 118.2, 116.9, 65.1, 50.8, 21.9, 21.8. HRMS-ESI (m/z) [M+Na]^+^ calcd for C_25_H_23_NNaO_4_S: 456.1240; found: 456.1228.

***N*-benzyl-6-bromo-*N*-tosyl-2*H*-chromene-3-carboxamide (CATB-6-Br)**

White solid, 95% yield. ^1^H NMR (600 MHz, CDCl_3_), *δ* (ppm): 7.60 (d, *J* = 8.2 Hz, 2H), 7.33-7.26 (m, 8H), 7.03 (d, *J* = 2.5 Hz, 1H), 6.70 (d, *J* = 8.6 Hz, 1H), 6.61 (s, 1H), 4.91 (s, 2H), 4.78 (s, 2H), 2.45 (s, 3H). ^13^C NMR (150 MHz, CDCl_3_), *δ* (ppm): 168.9, 153.9, 145.2, 136.1, 136.0, 134.4, 131.2, 130.6, 129.9, 128.9, 128.4, 128.3, 128.2, 128.1, 122.4, 118.1, 113.8, 65.2, 50.3, 21.8. HRMS-ESI (m/z) [M+Na]^+^ calcd for C_24_H_20_BrNNaO_4_S: 520.0189; found: 520.0177.

***N*-benzyl-7-bromo-*N*-tosyl-*2H*-chromene-3-carboxamide (CATB-7-Br)**

White solid, 46% yield. ^1^H NMR (600 MHz, CDCl_3_), *δ* (ppm): 7.63 (d, *J* = 8.3 Hz, 2H), 7.33-7.26 (m, 7H), 7.04 (dd, *J* = 8.1, 1.8 Hz, 1H), 6.99 (d, *J* = 1.3 Hz, 1H), 6.82 (d, *J* = 8.1 Hz, 1H), 6.76 (s, 1H), 4.88 (s, 2H), 4.76 (s, 2H), 2.44 (s, 3H). ^13^C NMR (150 MHz, CDCl_3_), *δ* (ppm): 169.0, 155.6, 145.1, 136.1, 135.9, 131.7, 129.91, 129.88, 128.9, 128.4, 128.3, 128.1, 127.0, 125.4, 125.2, 119.8, 119.6, 65.3, 50.5, 21.8. HRMS-ESI (m/z) [M+Na]^+^ calcd for C_24_H_20_BrNNaO_4_S: 520.0189; found: 520.0182.

***N*-benzyl-6-chloro-*N*-tosyl-2*H*-chromene-3-carboxamide (CATB-6-Cl)**

White solid, 46% yield. ^1^H NMR (600 MHz, CDCl_3_), *δ* (ppm): 7.61 (d, *J* = 8.3 Hz, 2H), 7.32-7.26 (m, 7H), 7.15 (dd, *J* = 8.6, 2.5 Hz, 1H), 6.90 (d, *J* = 2.5 Hz, 1H), 6.75 (d, *J* = 8.6 Hz, 1H), 6.62 (s, 1H), 4.91 (s, 2H), 4.78 (s, 2H), 2.45 (s, 3H). ^13^C NMR (150 MHz, CDCl_3_), *δ* (ppm): 169.0, 153.5, 145.2, 136.3, 131.6, 130.7, 129.9, 128.9, 128.5, 128.4, 128.3, 128.2, 126.8, 122.0, 117.7, 65.4, 50.4, 21.8. HRMS-ESI (m/z) [M+Na]^+^ calcd for C_24_H_20_ClNNaO_4_S: 476.0694; found: 476.0679.

***N*-benzyl-7-chloro-*N*-tosyl-2*H*-chromene-3-carboxamide (CATB-7-Cl)**

White solid, 47% yield. ^1^H NMR (600 MHz, CDCl_3_), *δ* (ppm): 7.61 (d, *J* = 7.9 Hz, 2H), 7.30-7.26 (m, 7H), 6.87 (s, 2H), 6.81 (s, 1H), 6.76 (s, 1H), 4.86 (s, 2H), 4.75 (s, 2H), 2.42 (s, 3H). ^13^C NMR (150 MHz, CDCl_3_), *δ* (ppm): 169.0, 155.6, 145.1, 137.3, 136.1, 135.9, 131.8, 129.9, 129.7, 128.9, 128.4, 128.3, 128.1, 126.8, 122.3, 119.2, 116.9, 65.3, 50.6, 21.8. HRMS-ESI (m/z) [M+Na]^+^ calcd for C_24_H_20_ClNNaO_4_S: 476.0694, found: 476.0678.

***N*-benzyl-*N*,1-ditosyl-1,2-dihydroquinoline-3-carboxamide (QATB-N-Ts)**

White solid, 92% yield. ^1^H NMR (600 MHz, CDCl_3_), *δ* (ppm): 7.77 (d, *J* = 8.1 Hz, 1H), 7.57 (d, *J* = 8.3 Hz, 2H), 7.39-7.37 (m, 1H), 7.33-7.30 (m, 3H), 7.27-7.25 (m, 2H), 7.22-7.19 (m, 3H), 7.10 (d, *J* = 8.3 Hz, 2H), 6.91 (dd, *J* = 7.5, 1.0 Hz, 1H), 6.76 (d, *J* = 8.1 Hz, 2H), 6.36 (s, 1H), 4.72 (s, 2H), 4.52 (s, 2H), 2.44 (s, 3H), 2.20 (s, 3H). ^13^C NMR (150 MHz, CDCl_3_), *δ* (ppm): 169.2, 145.0, 143.6, 136.3, 136.2, 136.1, 135.4, 132.1, 130.6, 130.0, 129.9, 129.0, 128.9, 128.8, 128.4, 128.3, 128.1, 127.8, 127.4, 126.92, 126.87, 50.6, 45.4, 21.8, 21.6. HRMS-ESI (m/z) [M+Na]^+^ calcd for C_31_H_28_N_2_NaO_5_S_2_: 595.1332; found: 595.1329.

**3. Photophysical properties**

**Figure S1. 2*H*-chromene (2*H*-1-benzopyran) (I), 4*H*-chromene (4*H*-1-benzopyran) (II), 2*H*-chromen-2-one (III), and 4*H*-chromen-4-one (IV).**


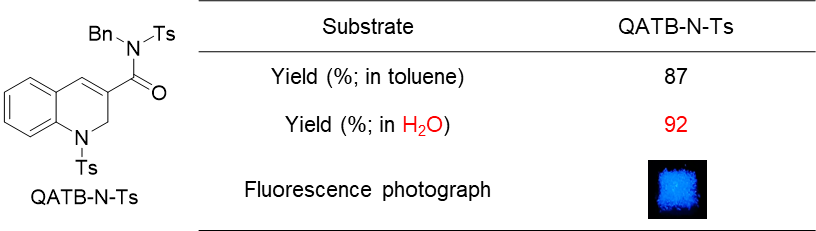


**Figure S2. Chemical structure, isolation yield in toluene and water, and its fluorescence image of QATB-N-Ts taken under a 365 nm UV lamp.**

**Figure S3.** **Absorption spectra of chromene-based BioAIEgens in dilute ACN solutions. Concentration: 20 μM.**


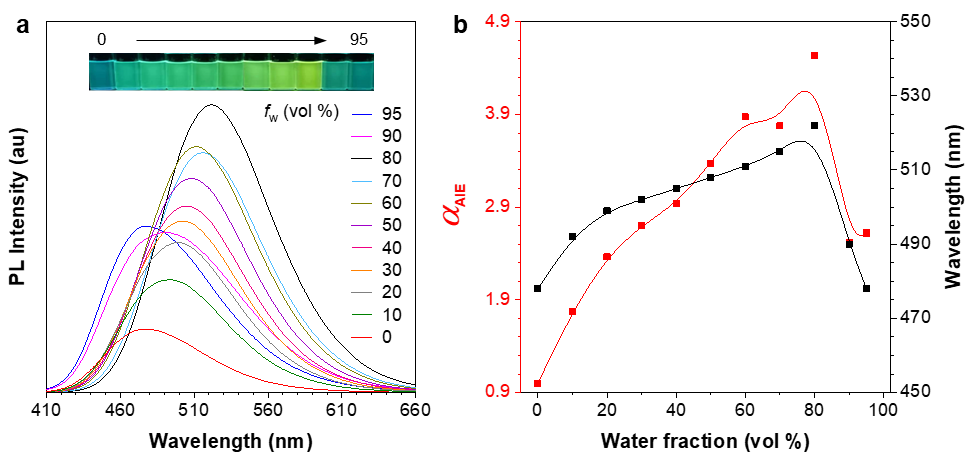


**Figure S4. Aggregation-induced emission properties of CATB.** (a) PL spectra of CATB in ACN/H_2_O mixtures with different *f*_w_. Concentration: 20 μM; *λ*_ex_: 345 nm. Inset: fluorescence images of CATB in ACN/H_2_O mixtures with different *f_w_* taken under a 365 nm UV lamp. (b) The plots of the *α*_AIE_ and maximum emission wavelength versus the composition of the aqueous mixture of CATB. *α*_AIE_ = *I/I*_0_, *I*_0_ = PL intensity in pure ACN.

**Figure S5. Absorption spectra of CATB-6-OMe in solvents with different polarities. Concentration: 20 μM.**


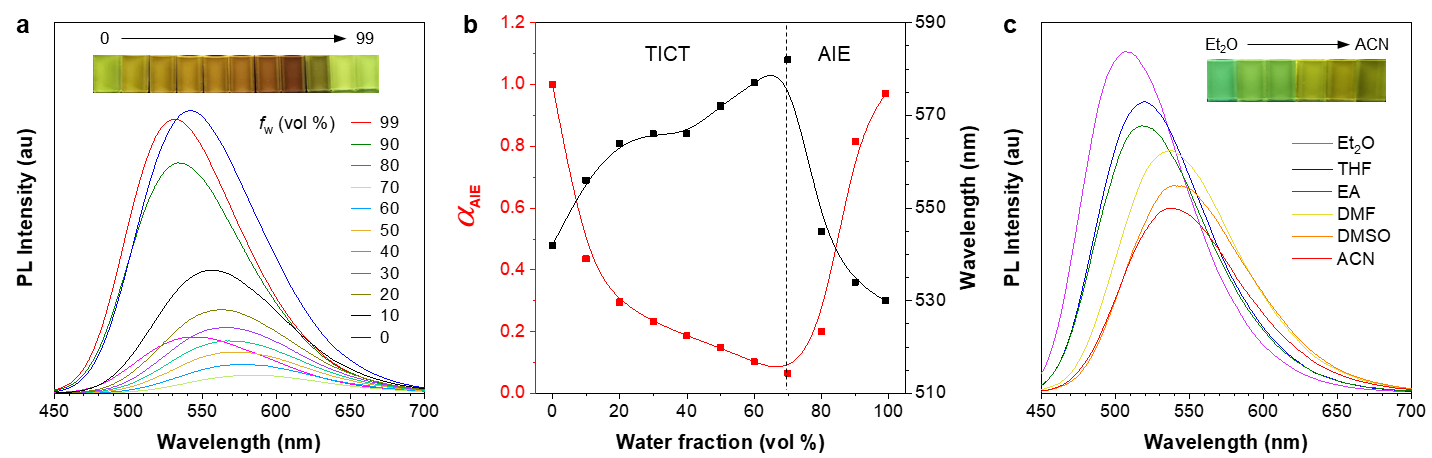


**Figure S6.** **Aggregation-induced emission properties of CATB-6-OMe.** (a) PL spectra of CATB-6-OMe in ACN/H_2_O mixtures with different *f_w_*. Concentration: 20 μM; *λ*_ex_: 365 nm. Inset: fluorescence photos of CATB-6-OMe in ACN/H_2_O mixtures with different *f_w_* taken under a 365 nm UV lamp. (b) The plots of the *α*_AIE_ and maximum emission wavelength versus the composition of the aqueous mixture of CATB-6-OMe. *α*_AIE_ = *I/I_0_*, *I_0_* = PL intensity in pure ACN (c) PL spectra of CATB-6-OMe in solvents with different polarities. Concentration: 20 μM. The absorption maximum of each solution was chosen as its excitation wavelength. Inset: fluorescence photos of CATB-6-OMe in organic solvents with different polarities taken under a 365 nm UV lamp.


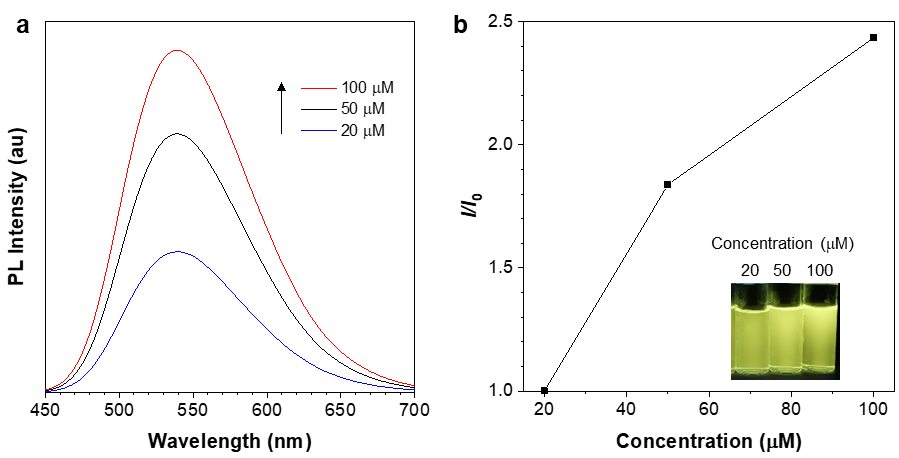


**Figure S7. Concentration effect of CATB-6-OMe.** (a) PL spectra of CATB-6-OMe in ACN solution with different concentrations. *λ*_ex_: 365 nm. (b) The plots of the emission intensity at the maximum versus the concentration of CATB-6-OMe in ACN. *I_0_* = PL intensity of CATB-6-OMe in ACN solution (20 μM). Inset: fluorescence photographs of CATB-6-OMe in ACN solution with different concentrations taken under a 365 nm UV lamp.


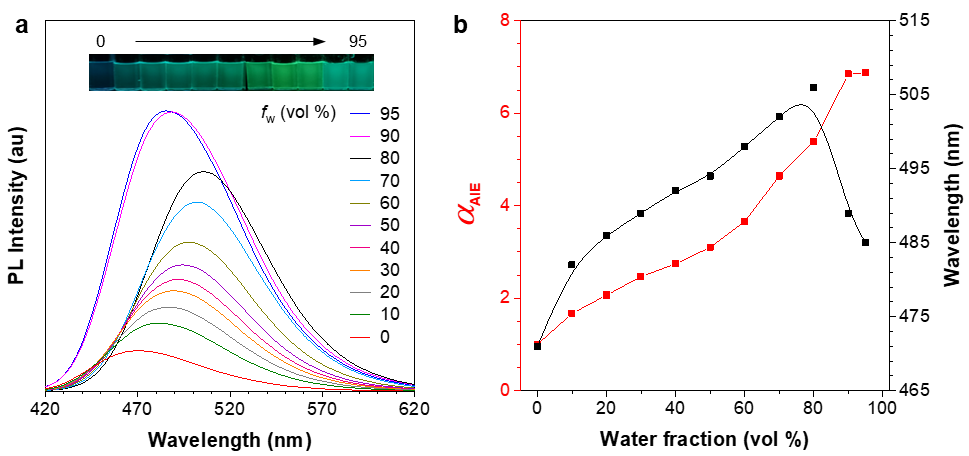


**Figure S8. Aggregation-induced emission properties of CATB-7-OMe.** (a) PL spectra of CATB-7-OMe in ACN/H_2_O mixtures with different *f_w_*. Concentration: 20 μM; *λ*_ex_: 355 nm. Inset: fluorescence images of CATB-7-OMe in ACN/H_2_O mixtures with different *f_w_* taken under a 365 nm UV lamp. (b) The plots of the *α*_AIE_ and maximum emission wavelength versus the composition of the aqueous mixture of CATB-7-OMe. *α*_AIE_ = *I/I_0_*, *I_0_* = PL intensity in pure ACN.


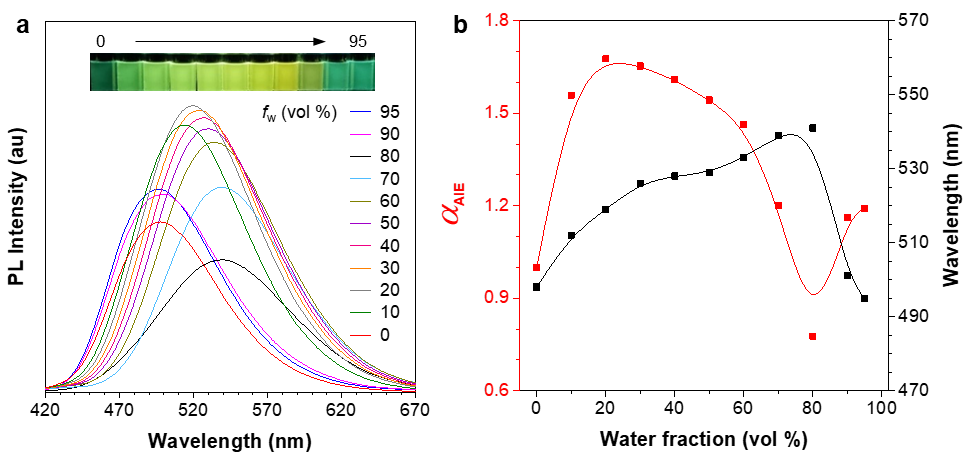


**Figure S9. Aggregation-induced emission properties of CATB-6-Me.** (a) PL spectra of CATB-6-Me in ACN/H_2_O mixtures with different *f_w_*. Concentration: 20 μM; *λ*_ex_: 355 nm. Inset: fluorescence images of CATB-6-Me in ACN/H_2_O mixtures with different *f_w_* taken under a 365 nm UV lamp. (b) The plots of the *α*_AIE_ and maximum emission wavelength versus the composition of the aqueous mixture of CATB-6-Me. *α*_AIE_ = *I/I_0_*, *I_0_* = PL intensity in pure ACN.


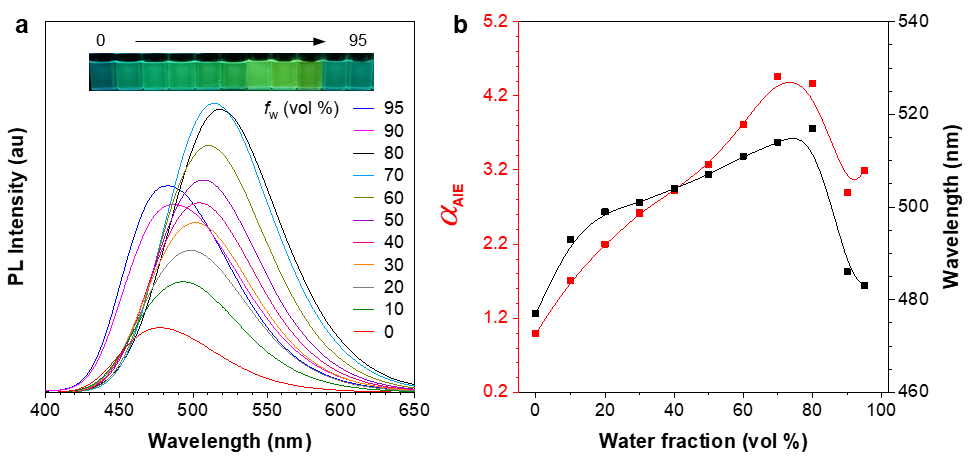


**Figure S10. Aggregation-induced emission properties of CATB-7-Me.** (a) PL spectra of CATB-7-Me in ACN/H_2_O mixtures with different *f_w_*. Concentration: 20 μM; *λ*_ex_: 348 nm. Inset: fluorescence images of CATB-7-Me in ACN/H_2_O mixtures with different *f_w_* taken under a 365 nm UV lamp. (b) The plots of the *α*_AIE_ and maximum emission wavelength versus the composition of the aqueous mixture of CATB-7-Me. *α*_AIE_ = *I/I_0_*, *I_0_* = PL intensity in pure ACN.


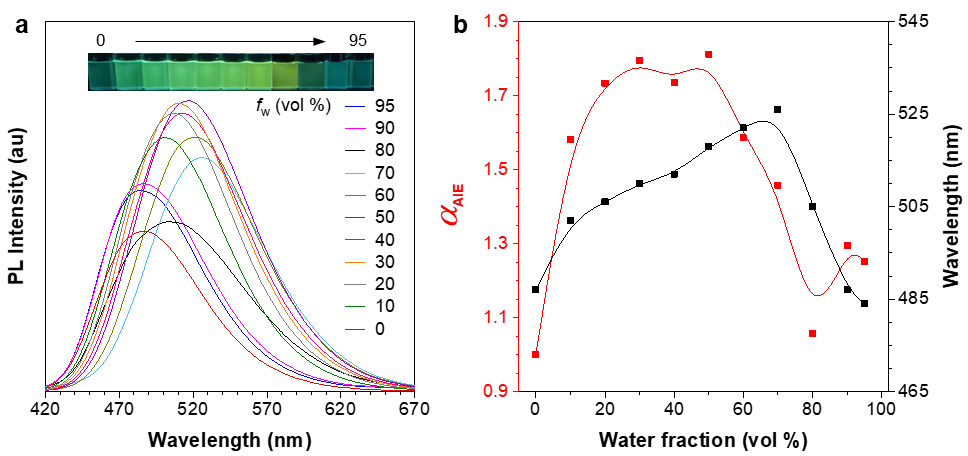


**Figure S11. Aggregation-induced emission properties of CATB-6-Br.** (a) PL spectra of CATB-6-Br in ACN/H_2_O mixtures with different *f_w_*. Concentration: 20 μM; *λ*_ex_: 345 nm. Inset: fluorescence images of CATB-6-Br in ACN/H_2_O mixtures with different *f_w_* taken under a 365 nm UV lamp. (b) The plots of the *α*_AIE_ and maximum emission wavelength versus the composition of the aqueous mixture of CATB-6-Br. *α*_AIE_ = *I/I_0_*, *I_0_* = PL intensity in pure ACN.


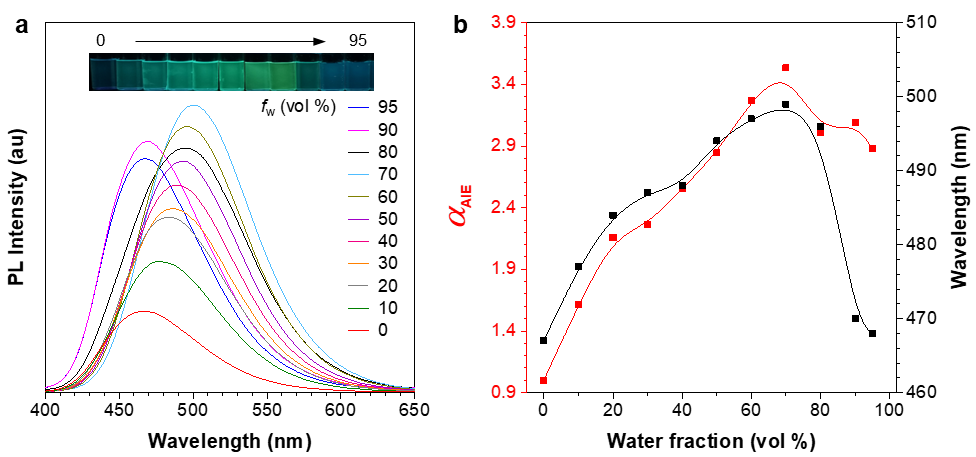


**Figure S12. Aggregation-induced emission properties of CATB-7-Br.** (a) PL spectra of CATB-7-Br in ACN/H_2_O mixtures with different *f_w_*. Concentration: 20 μM; *λ*_ex_: 338 nm. Inset: fluorescence images of CATB-7-Br in ACN/H_2_O mixtures with different *f_w_* taken under a 365 nm UV lamp. (b) The plots of the *α*_AIE_ and maximum emission wavelength versus the composition of the aqueous mixture of CATB-7-Br. *α*_AIE_ = *I/I_0_*, *I_0_* = PL intensity in pure ACN.


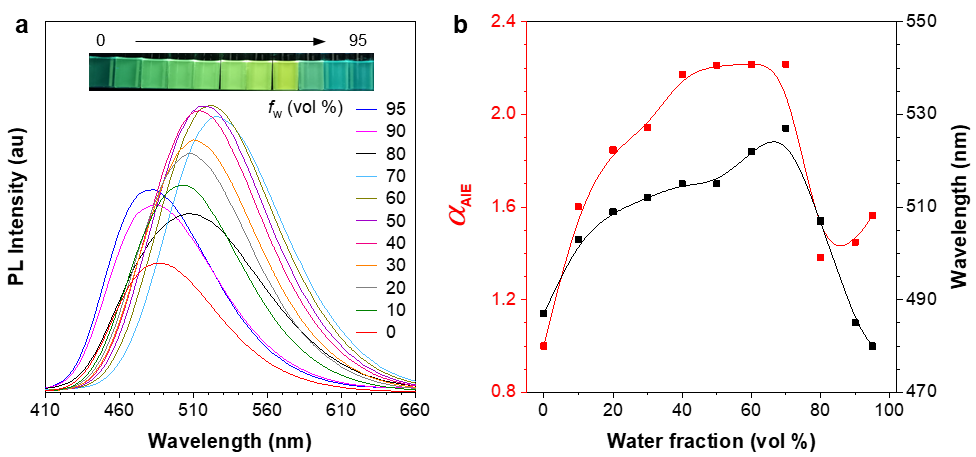


**Figure S13. Aggregation-induced emission properties of CATB-6-Cl.** (a) PL spectra of CATB-6-Cl in ACN/H_2_O mixtures with different *f_w_*. Concentration: 20 μM; *λ*_ex_: 345 nm. Inset: fluorescence images of CATB-6-Cl in ACN/H_2_O mixtures with different *f_w_* taken under a 365 nm UV lamp. (b) The plots of the *α*_AIE_ and maximum emission wavelength versus the composition of the aqueous mixture of CATB-6-Cl. *α*_AIE_ = *I/I_0_*, *I_0_* = PL intensity in pure ACN.


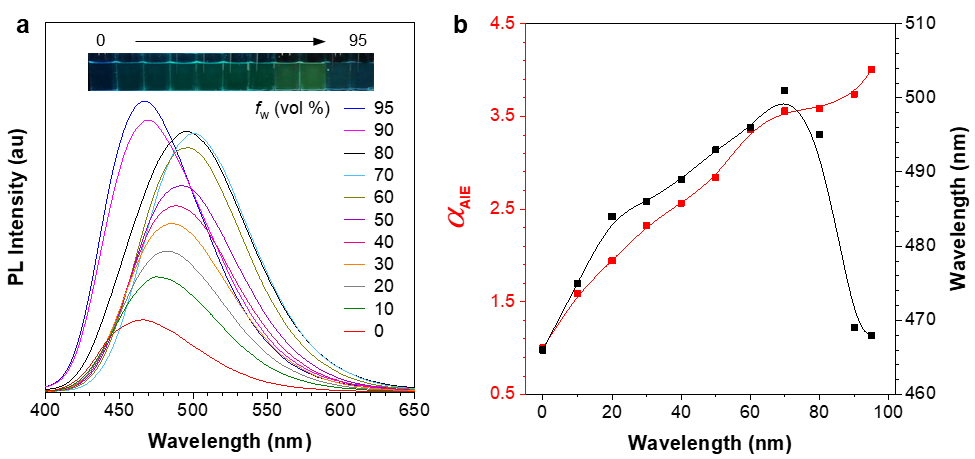


**Figure S14. Aggregation-induced emission properties of CATB-7-Cl.** (a) PL spectra of CATB-7-Cl in ACN/H_2_O mixtures with different *f_w_*. Concentration: 20 μM; *λ*_ex_: 338 nm. Inset: fluorescence images of CATB-7-Cl in ACN/H_2_O mixtures with different *f_w_* taken under a 365 nm UV lamp. (b) The plots of the *α*_AIE_ and maximum emission wavelength versus the composition of the aqueous mixture of CATB-7-Cl. *α*_AIE_ = *I/I_0_*, *I_0_* = PL intensity in pure ACN.


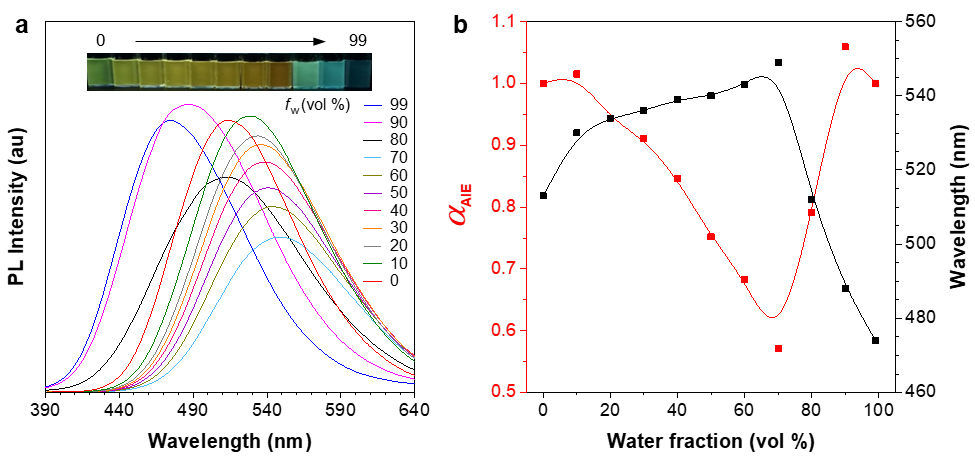


**Figure S15. Aggregation-induced emission properties of QATB-N-Ts.** (a) PL spectra of QATB-N-Ts in ACN/H_2_O mixtures with different *f_w_*. Concentration: 20 μM; *λ*_ex_: 335 nm. Inset: fluorescence images of QATB-N-Ts in ACN/H_2_O mixtures with different *f_w_* taken under a 365 nm UV lamp. (b) The plots of the *α*_AIE_ and maximum emission wavelength versus the composition of the aqueous mixture of QATB-N-Ts. *α*_AIE_ = *I/I_0_*, *I_0_* = PL intensity in pure ACN.


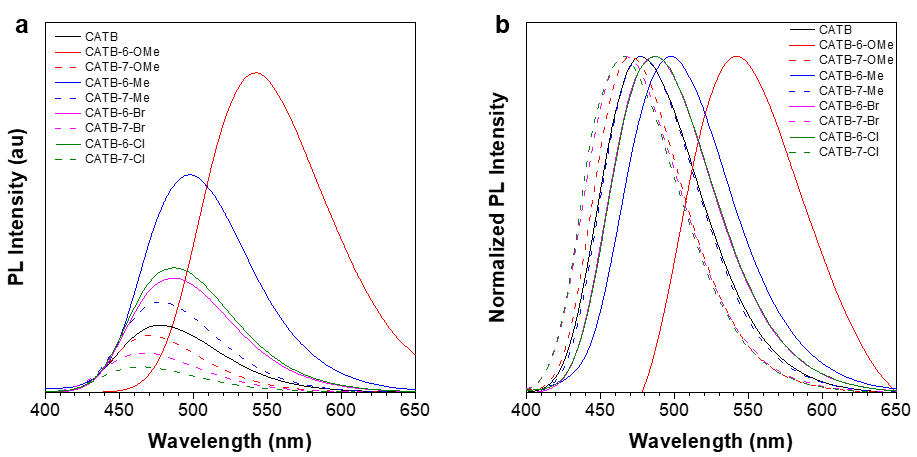


**Figure S16. Photophysical properties at molecular state.** (a) PL and (b) Normalized PL spectra of 6-/7-substituted products in dilute ACN solutions (20 μM). The absorption maximum of each solution was chosen as its excitation wavelength.


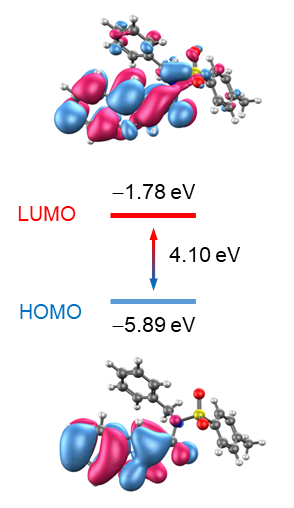


**Figure S17. Frontier molecular orbitals and corresponding energy levels of CATB based on the optimized ground-state geometry.**


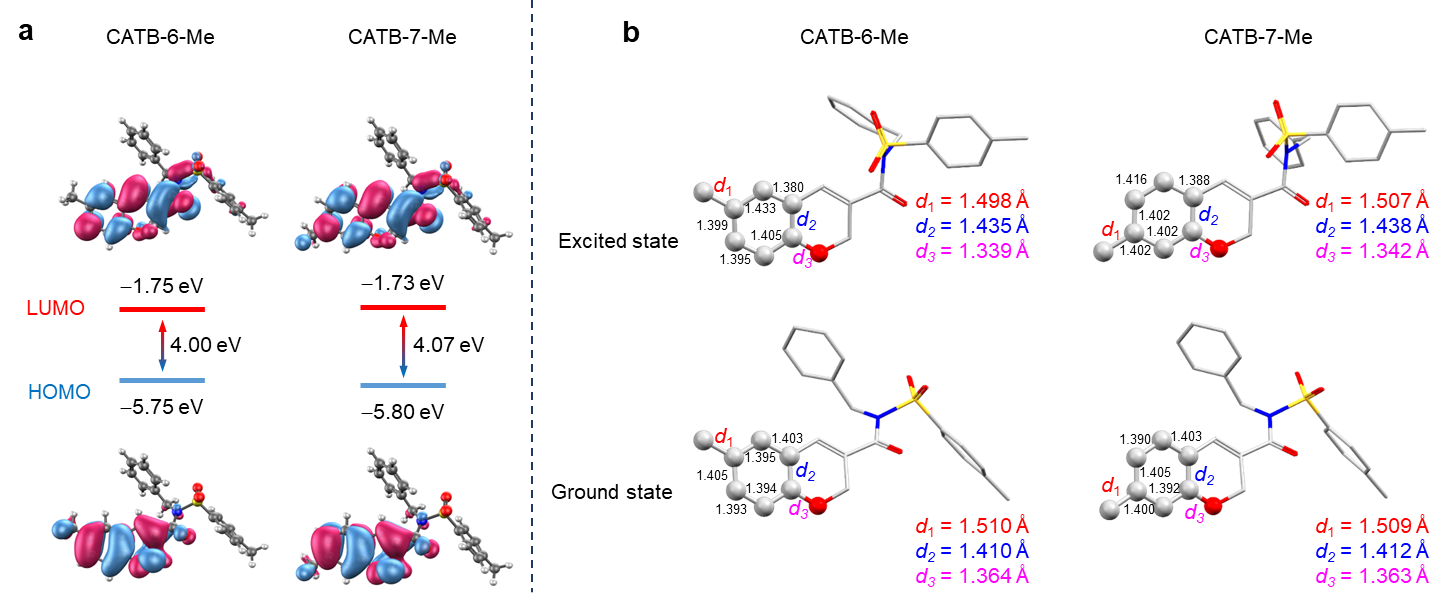


**Figure S18. Theoretical calculation of CATB-6-Me and CATB-7-Me.** (a) Frontier molecular orbitals and corresponding energy levels of CATB-6-Me and CATB-7-Me on their optimized ground-state geometry. (b) Calculated geometries of CATB-6-Me and CATB-7-Me in the ground and excited states.


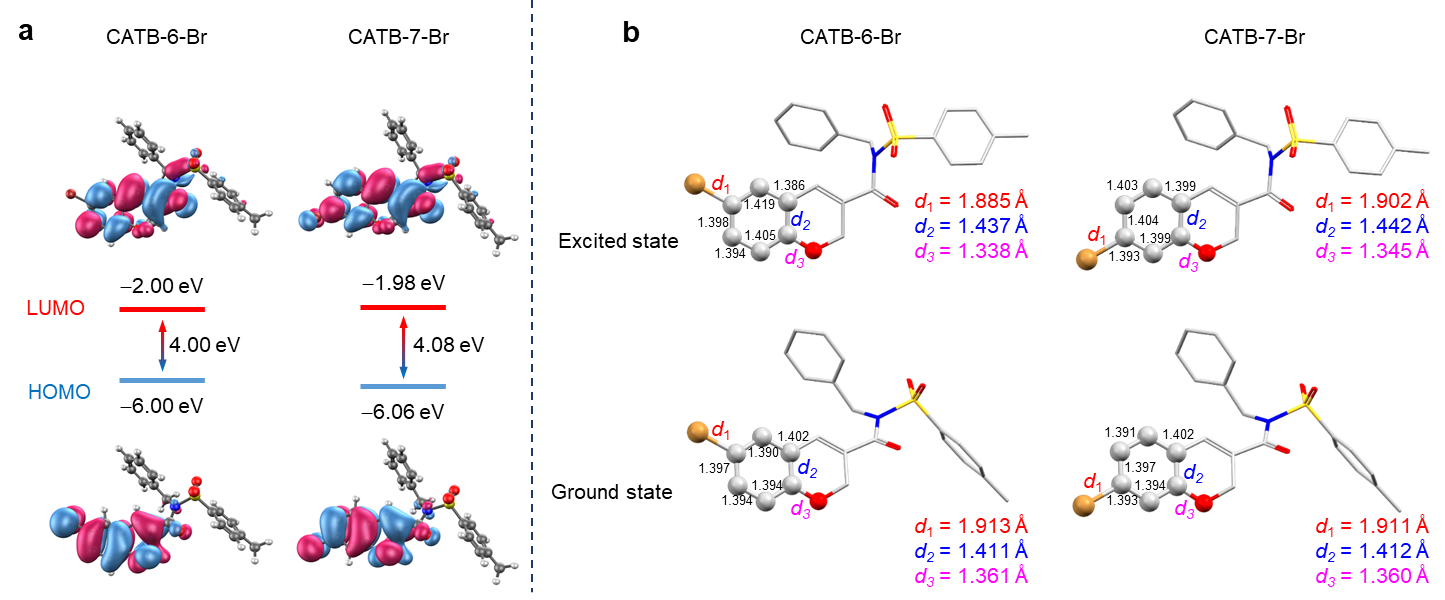


**Figure S19. Theoretical calculation of CATB-6-Br and CATB-7-Br.** (a) Frontier molecular orbitals and corresponding energy levels of CATB-6-Br and CATB-7-Br based on their optimized ground-state geometry. (b) Calculated geometries of CATB-6-Br and CATB-7-Br in ground and excited states.


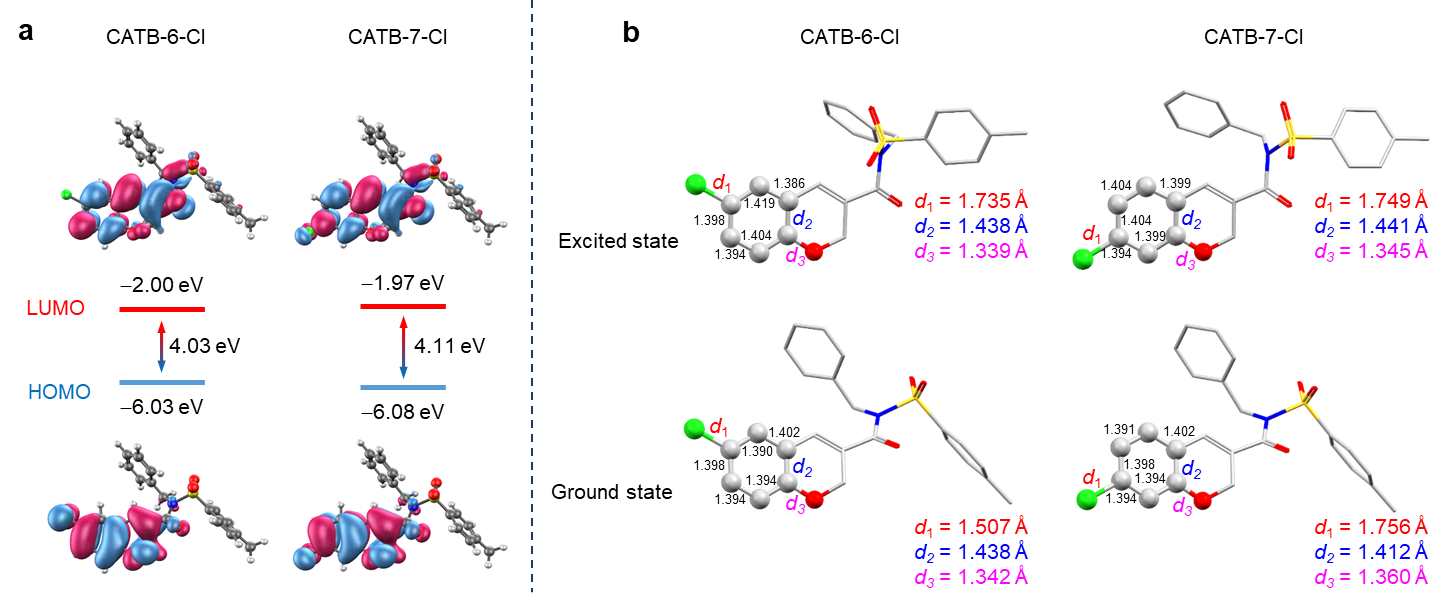


**Figure S20. Theoretical calculation of CATB-6-Cl and CATB-7-Cl.** (a) Frontier molecular orbitals and corresponding energy levels of CATB-6-Cl and CATB-7-Cl based on their optimized ground-state geometry. (b) Calculated geometries of CATB-6-Cl and CATB-7-Cl in ground and excited states.


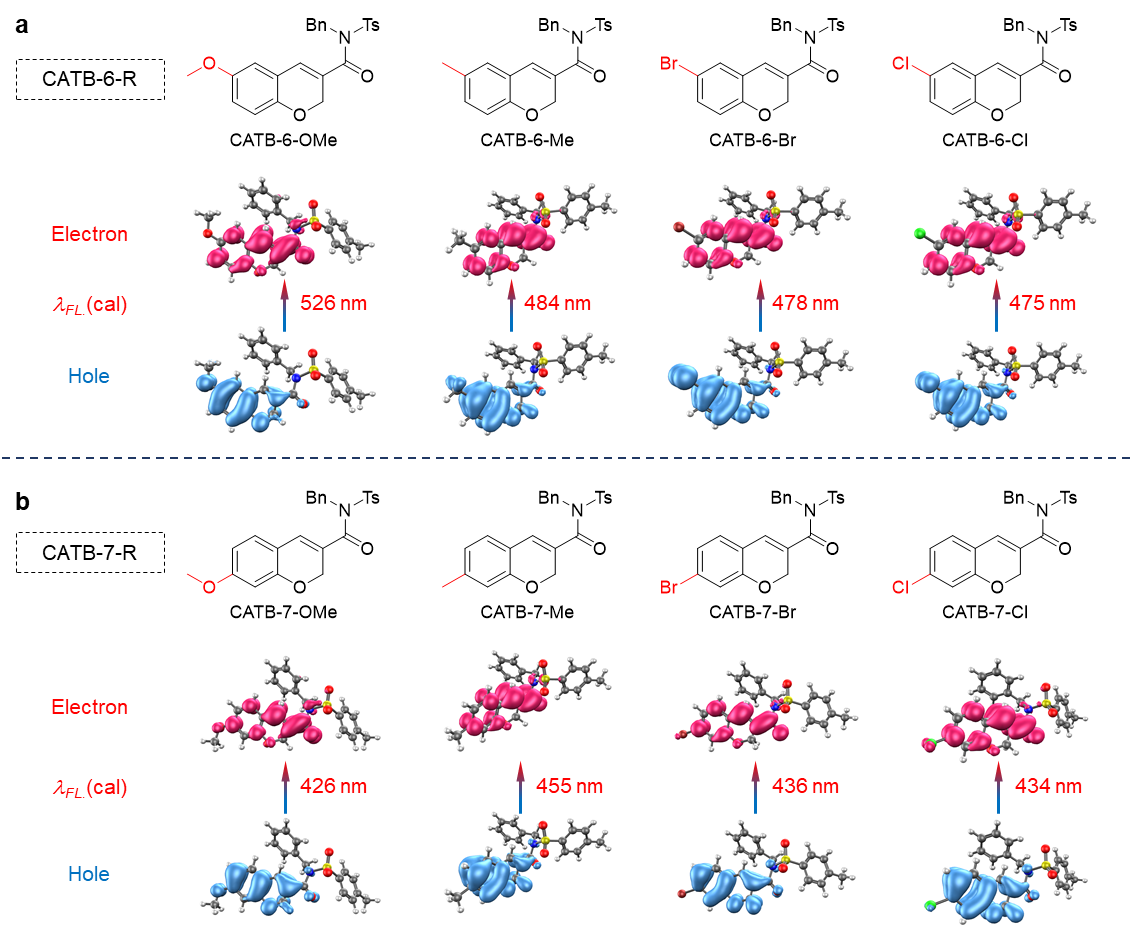


**Figure S21. Electron-hole analysis and calculated absorption wavelengths of 6-/7-substituted chromene-derived BioAIEgens based on their excited-state geometry.**


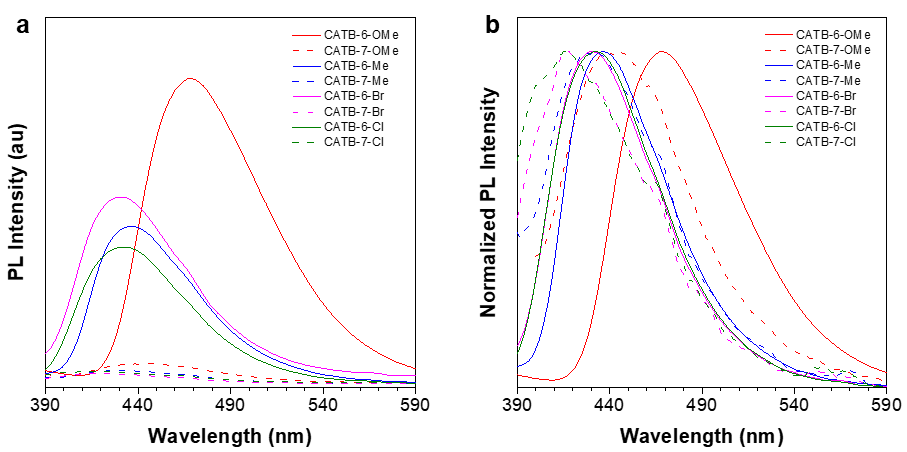


**Figure S22. Photophysical properties at crystalline state.** (a) PL and (b) normalized PL spectra of 6-/7-substituted products in the crystalline state. The absorption maximum of each solution was chosen as its excitation wavelength. C_b_ state is applied for CATB-6-OMe.


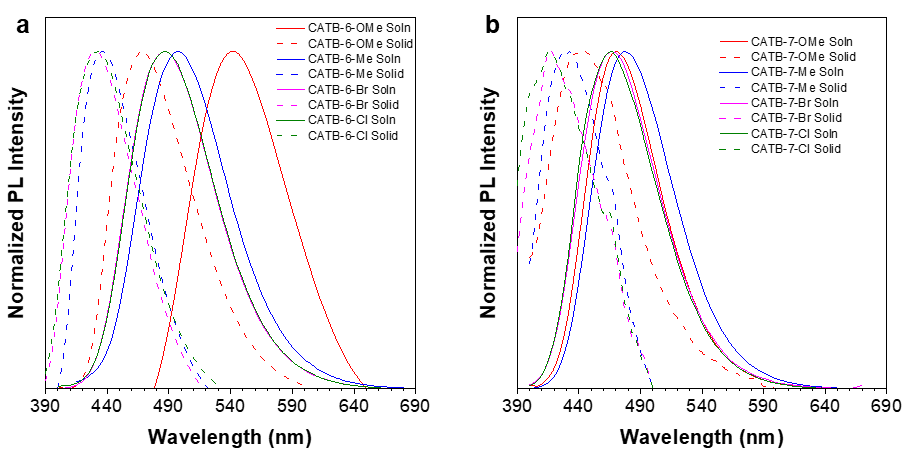


**Figure S23. Comparison of photophysical properties at molecular and crystalline state.** Normalized PL spectra of (a) 6- and (b) 7-substituted products in both solution (ACN solutions: 20 μM) and crystalline (solid) states. The absorption maximum of each sample was chosen as its excitation wavelength. C_b_ state is applied for CATB-6-OMe.

**Figure S24. PL spectra of crystalline CATB-6-OMe before (C_b_) and after (G_b_) grinding.**


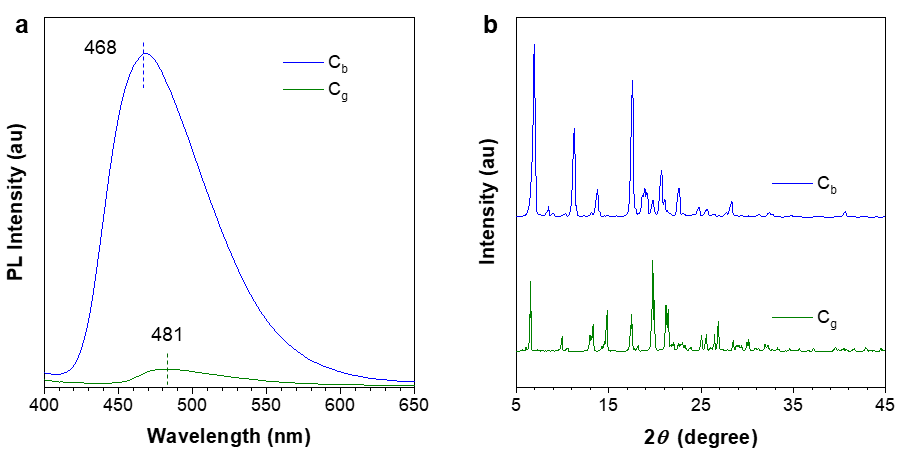


**Figure S25. (a) PL and (b) PXRD spectra of CATB-6-OMe as C_b_ and C_g_. *λ*_ex_: 365 nm.**


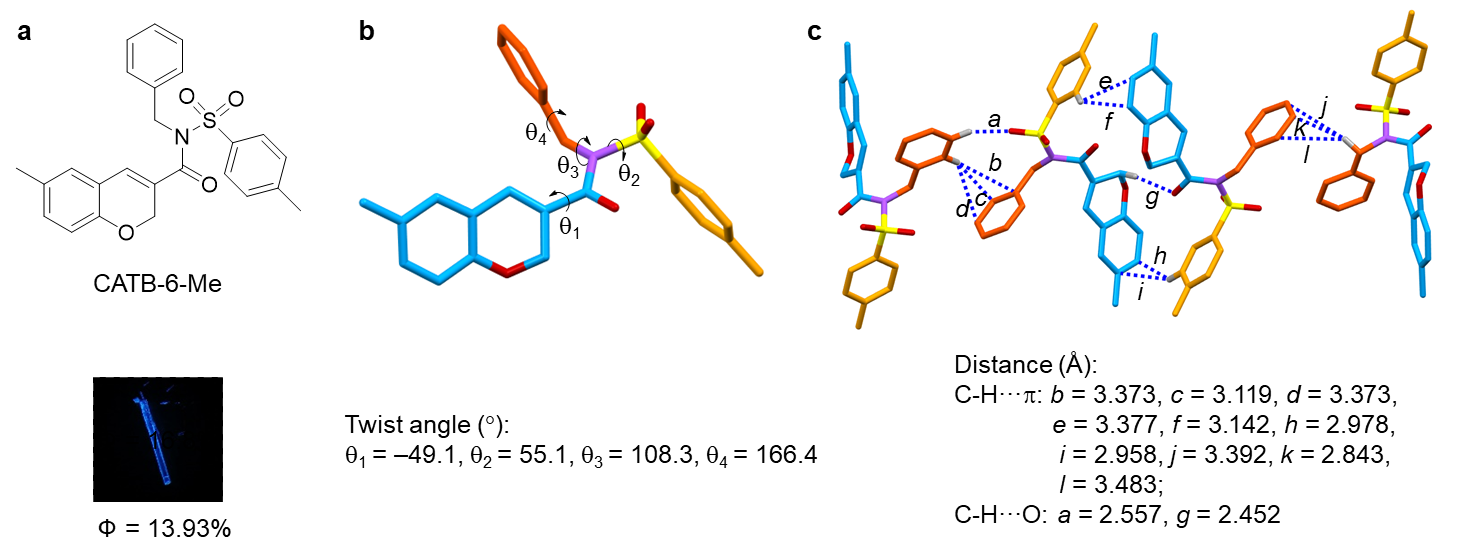


**Figure S26. Single crystal structure and crystal packing of CATB-6-Me.** (a) Chemical structure, fluorescence image of a single crystal taken under a 365 nm lamp, quantum yield, (b) molecular conformation, and (c) rigidifications of CATB-6-Me.

**Table S2.** Crystallographic data for compounds of C_b_ and C_g_ of CATB-6-OMe and CATB-6-Me.


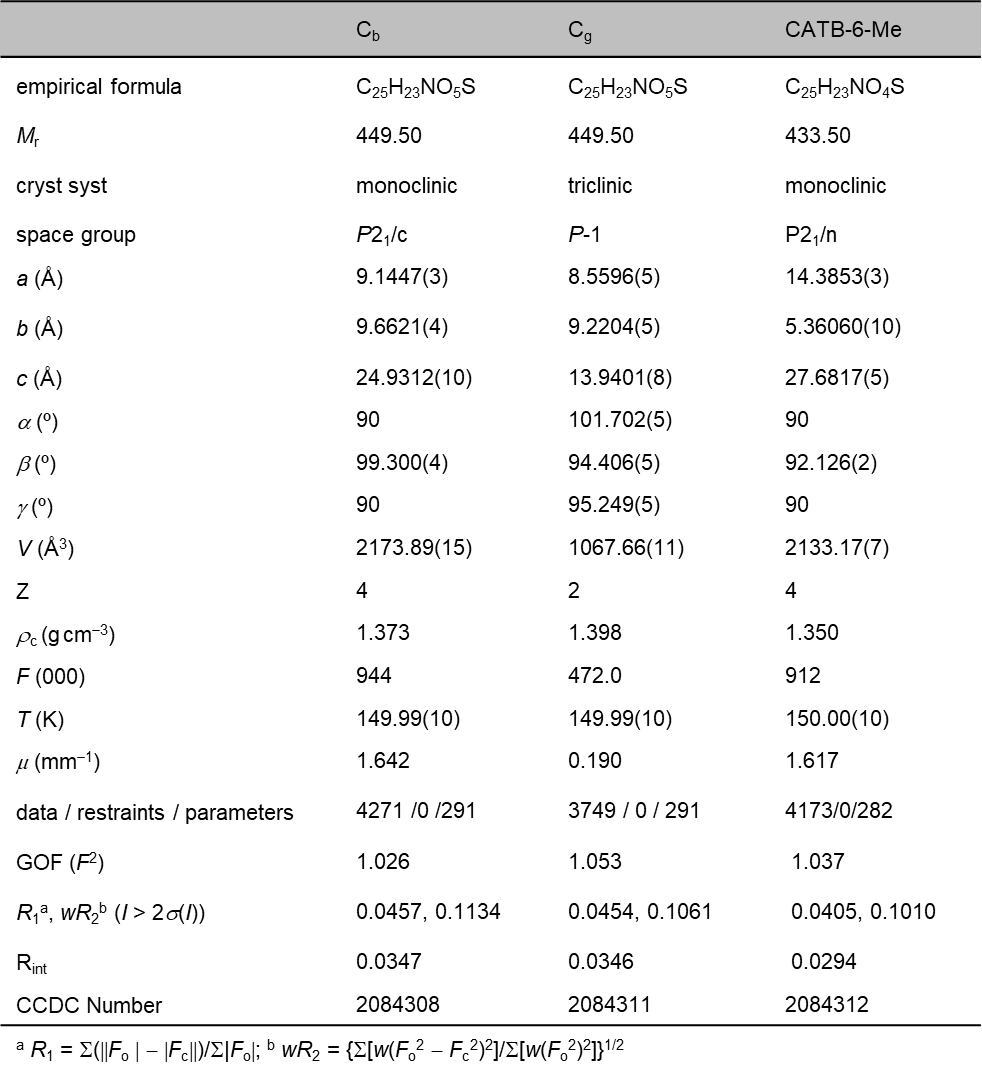


**4. MTT, live-dead cell staining experiments and ER-specific imaging**


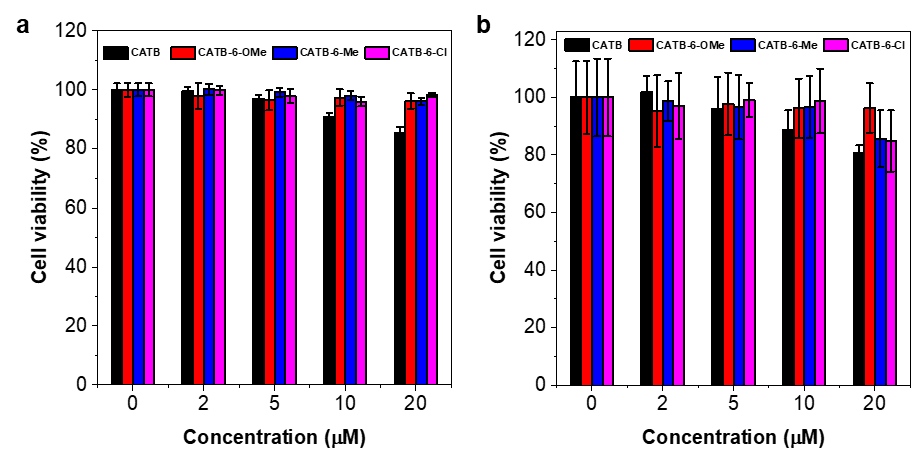


**Figure S27. Cytotoxicity study.** Cell viabilities of (a) A549 and (b) HeLa cells in the presence of different concentrations of CATB**,** CATB-6-OMe**,** CATB-6-Me, and CATB-6-Cl, respectively. Data are presented as mean ± SD (n = 5 independent experiments).


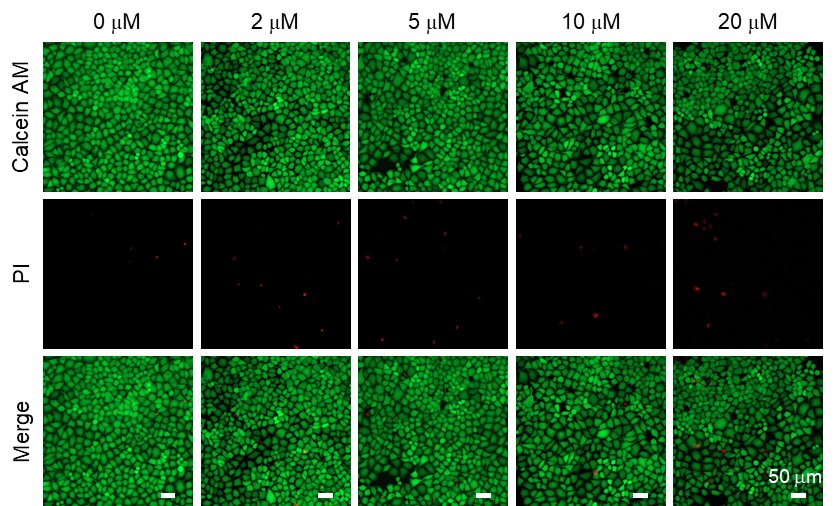


**Figure S28. Concentration-dependent live-dead cell staining test of CATB.** Fluorescence images of A549 cells incubated with different concentrations of CATB. The green fluorescence from Calcein AM (*λ*_ex_: 488 nm, *λ*_em_: 500-550 nm) and red fluorescence from PI (*λ*_ex_: 561 nm, *λ*_em_: 600-700 nm) represent live cell and dead cell, respectively. The fields of vision were randomly selected.


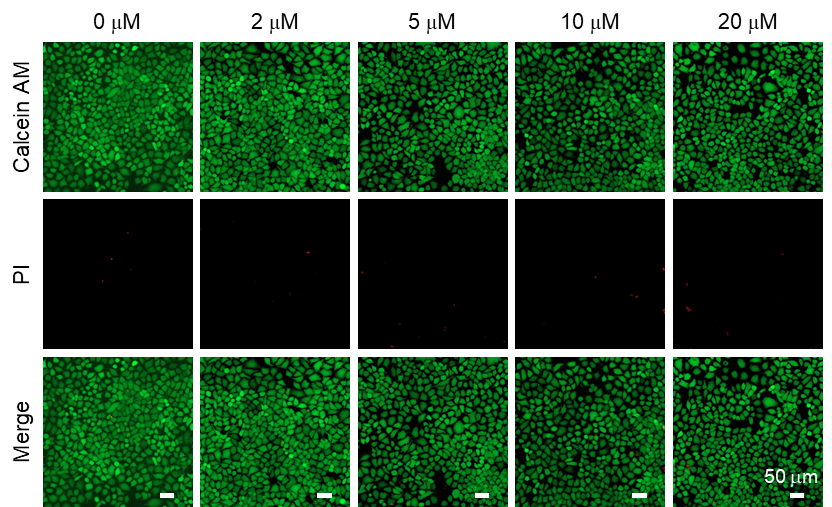


**Figure S29. Concentration-dependent live-dead cell staining test of CATB-6-OMe.** Fluorescence images of A549 cells incubated with different concentrations of CATB-6-OMe. The green fluorescence from Calcein AM (*λ*_ex_: 488 nm, *λ*_em_: 500-550 nm) and red fluorescence from PI (*λ*_ex_: 561 nm, *λ*_em_: 600-700 nm) represent live cell and dead cell, respectively. The fields of vision were randomly selected.


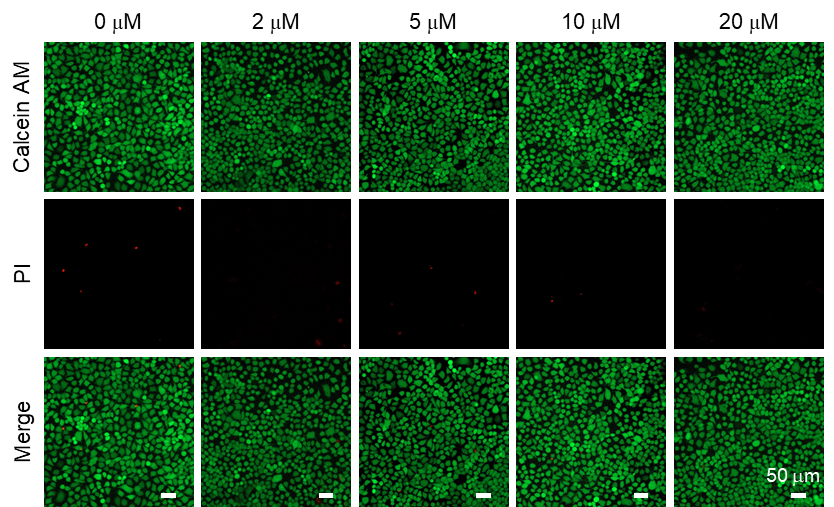


**Figure S30. Concentration-dependent live-dead cell staining test of CATB-6-Me.** Fluorescence images of A549 cells incubated with different concentrations of CATB-6-Me. The green fluorescence from Calcein AM (*λ*_ex_: 488 nm, *λ*_em_: 500-550 nm) and red fluorescence from PI (*λ*_ex_: 561 nm, *λ*_em_: 600-700 nm) represent live cell and dead cell, respectively. The fields of vision were randomly selected.


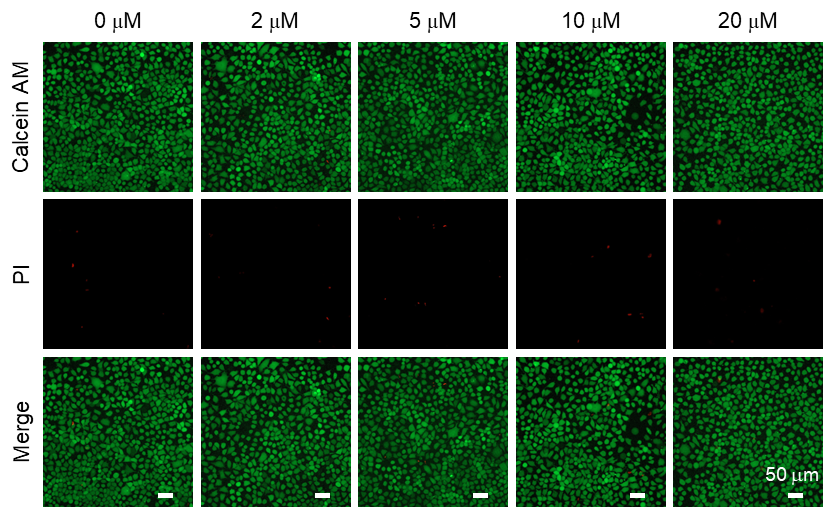


**Figure S31. Concentration-dependent live-dead cell staining test of CATB-6-Cl.** Fluorescence images of A549 cells incubated with different concentrations of CATB-6-Cl. The green fluorescence from Calcein AM (*λ*_ex_: 488 nm, *λ*_em_: 500-550 nm) and red fluorescence from PI (*λ*_ex_: 561 nm, *λ*_em_: 600-700 nm) represent live cell and dead cell, respectively. The fields of vision were randomly selected.


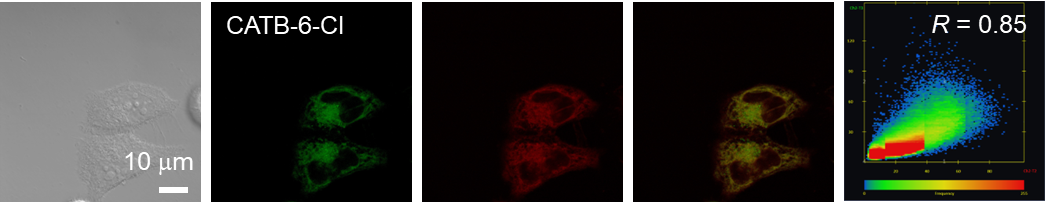


**Figure S32. ER-specific imaging of CATB-6-Cl in A549 cells.** Fluorescence images of A549 cells co-stained with 10 μM of CATB-6-Cl, and 1 μM of ER-Tracker Red for 30 min, respectively. Green channels for CATB-6-Cl: *λ*_ex_: 405 nm, *λ*_em_: 450-600 nm; red channel for ER-Tracker Red: *λ*_ex_: 561 nm, *λ*_em_: 600-700 nm. *R* = Pearson’s correlation coefficient.


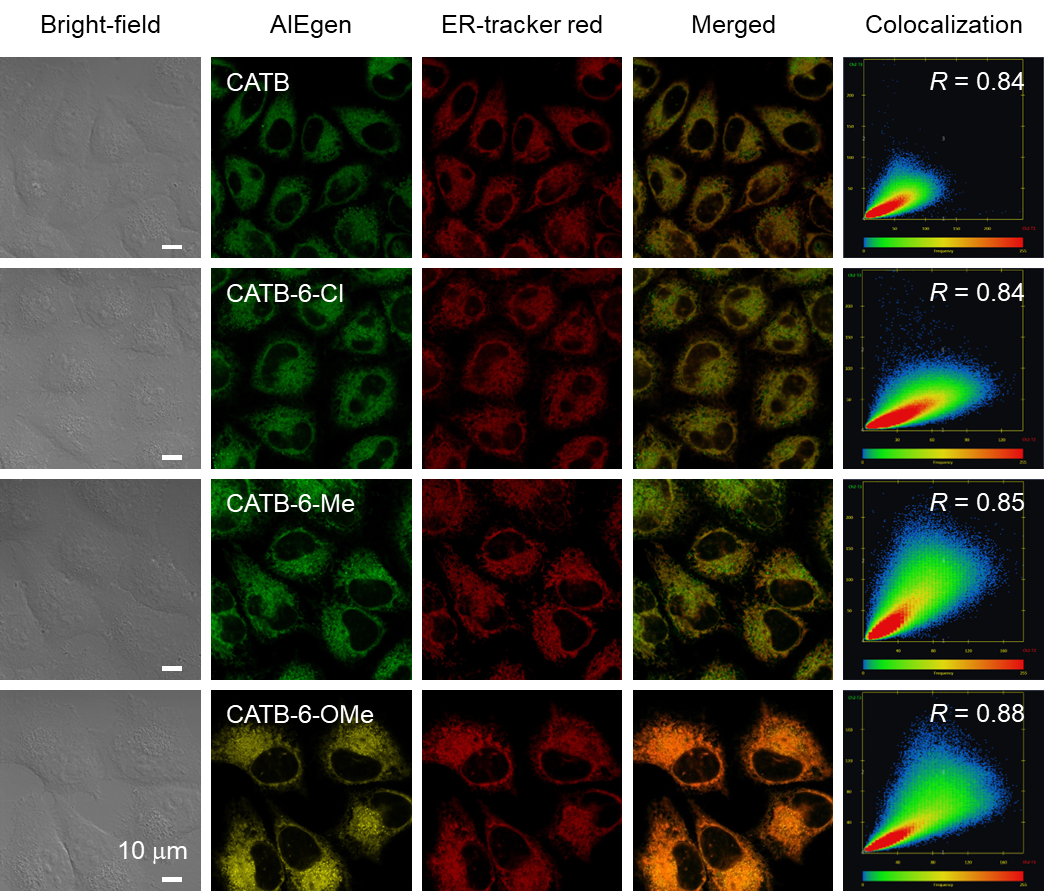


**Figure S33. ER-specific imaging in Hela cells.** Fluorescence images of HeLa cells co-stained with 10 μM of CATB, CATB-6-Cl, CATB-6-Me, CATB-6-OMe, and 1 μM of ER-Tracker red for 30 min, respectively. Green channels for CATB, CATB-6-Cl, CATB-6-Me, CATB-6-OMe: *λ*_ex_: 405 nm, *λ*_em_: 450-600 nm; Red channel for ER-Tracker red: *λ*_ex_: 561 nm, *λ*_em_: 600-700 nm. *R* = Pearson’s correlation coefficient.

**5. NMR and HRMS spectra**


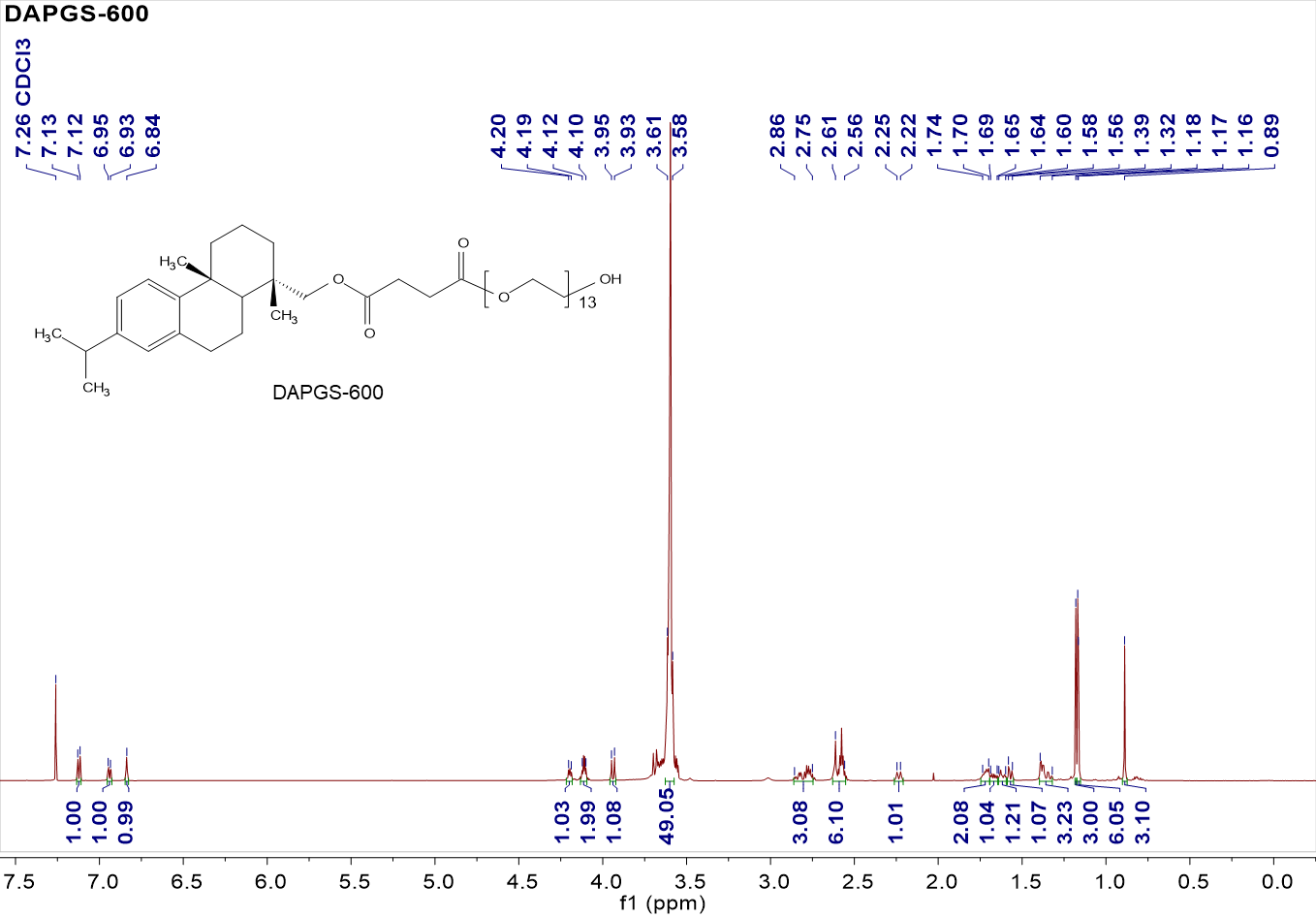


**Figure S34. ^1^H NMR spectrum of DAPGS-600.**


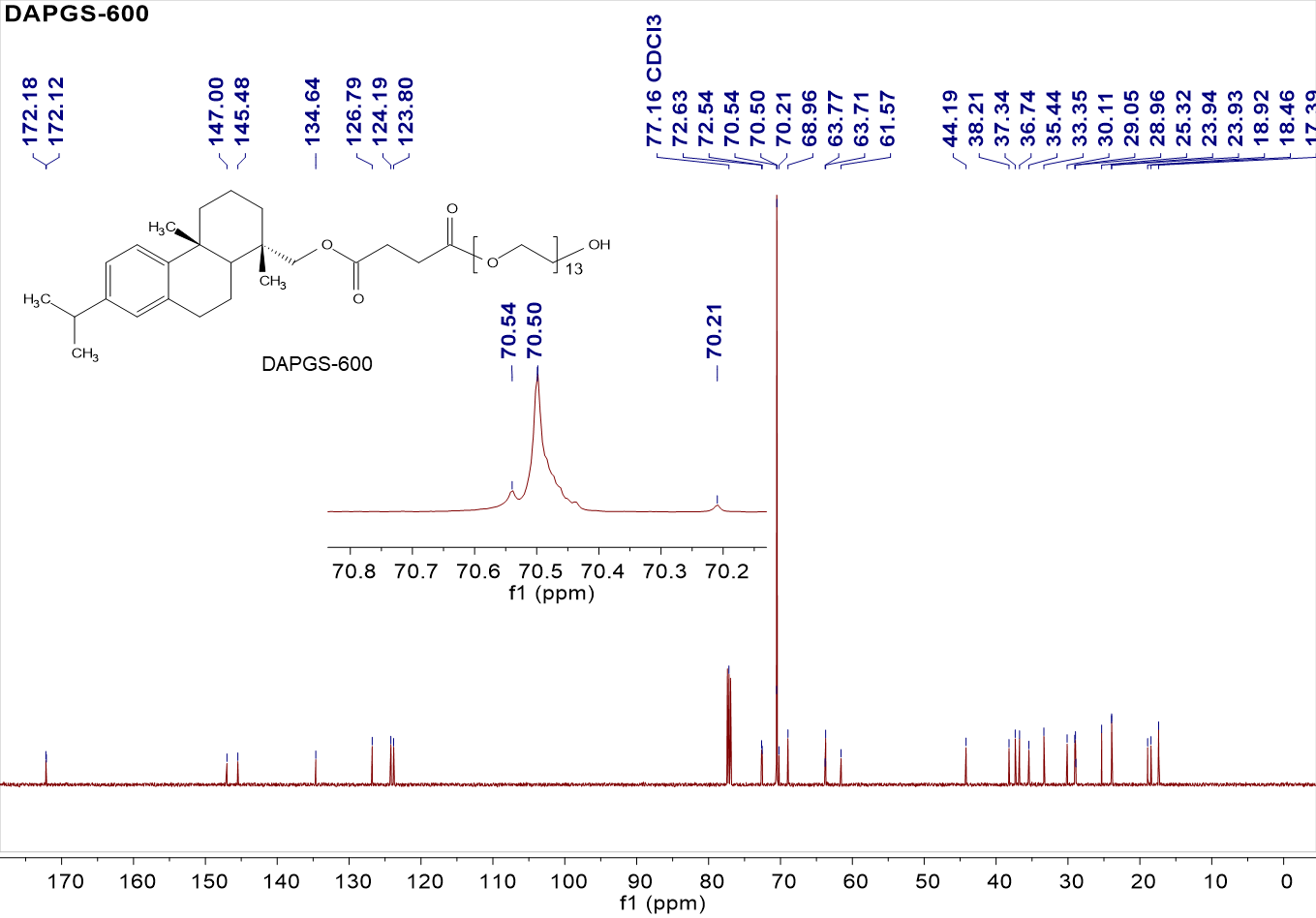


**Figure S35. ^13^C NMR spectrum of DAPGS-600.**


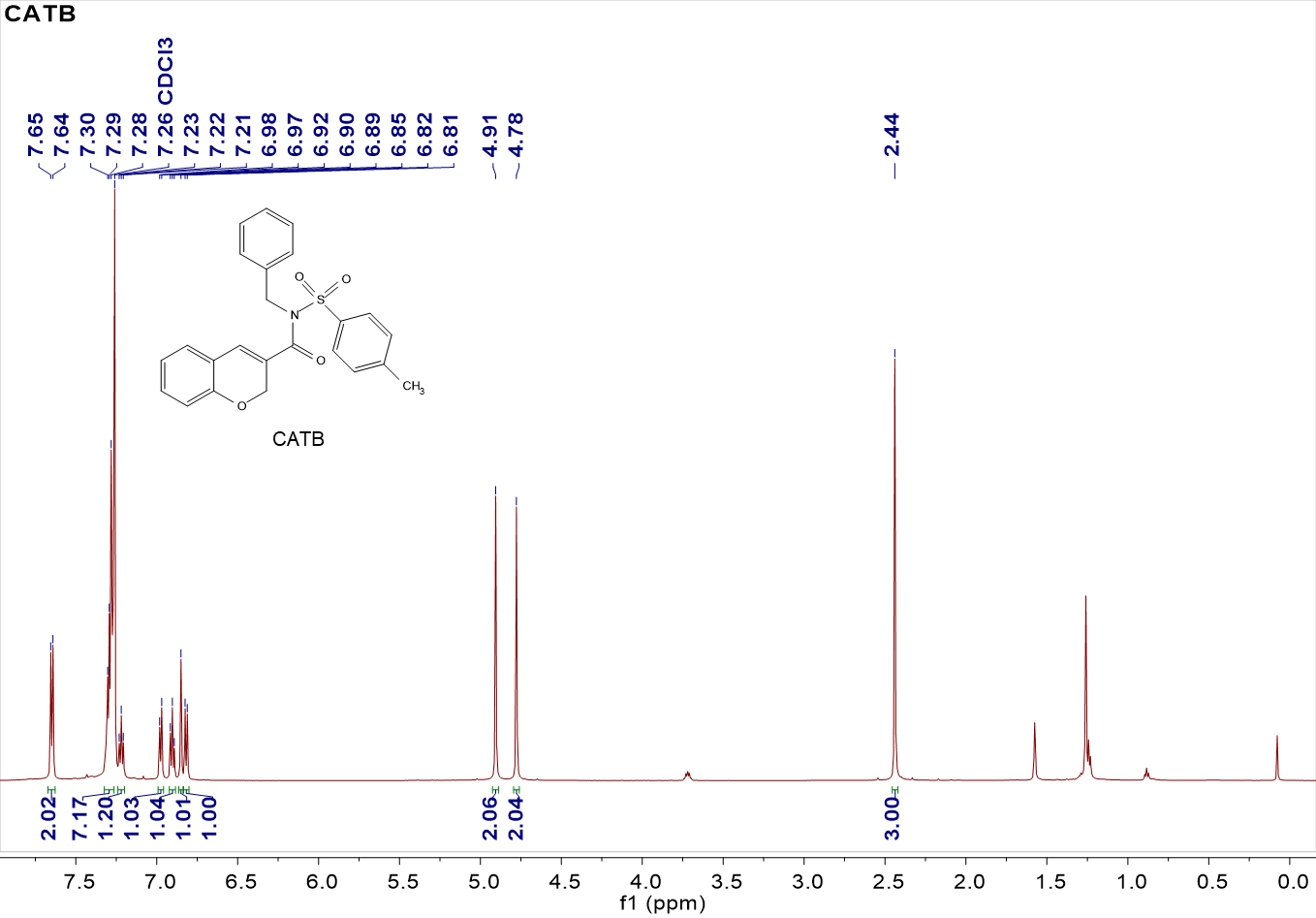


**Figure S36. ^1^H NMR spectrum of CATB.**


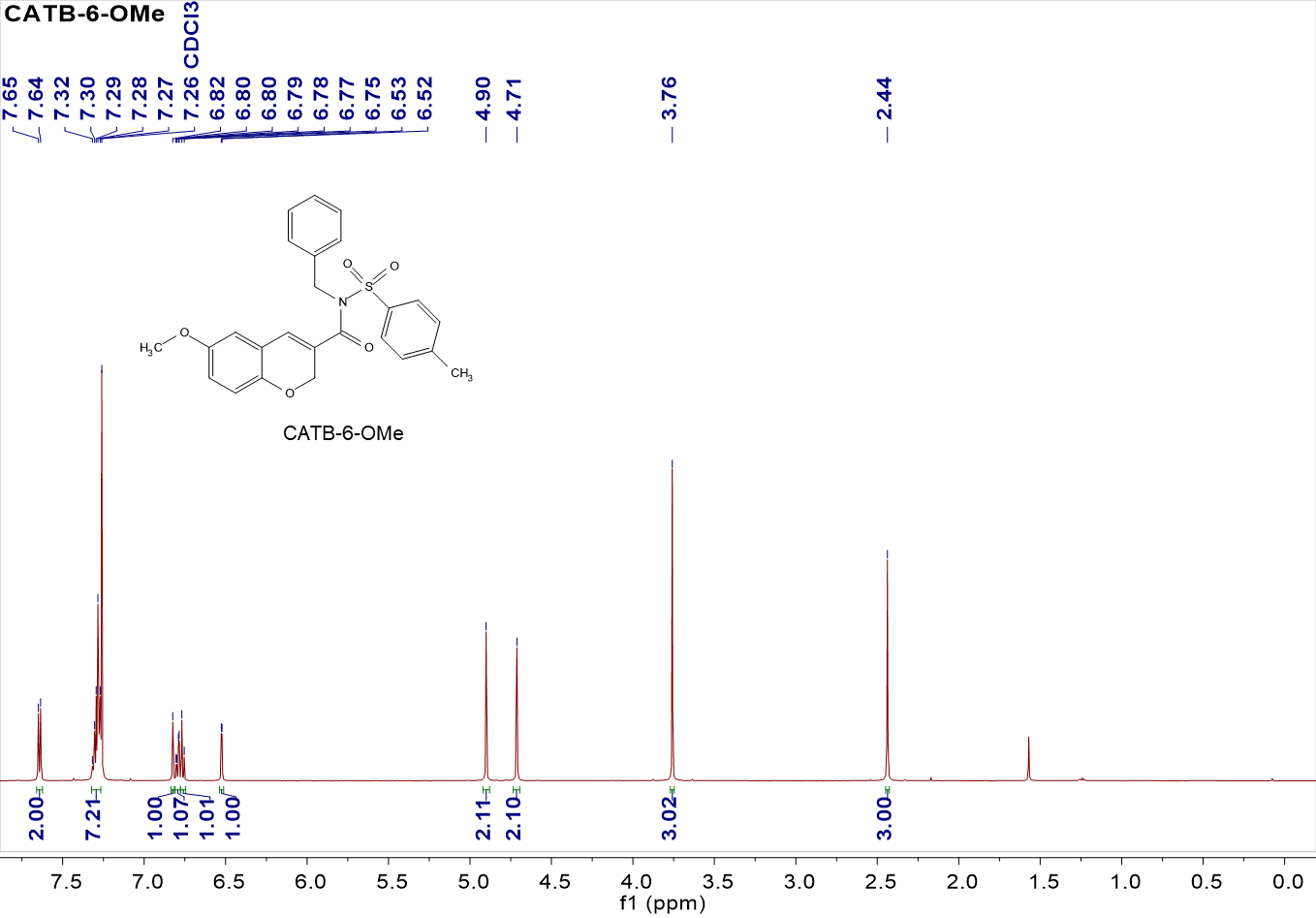


**Figure S37. ^1^H NMR spectrum of CATB-6-OMe.**


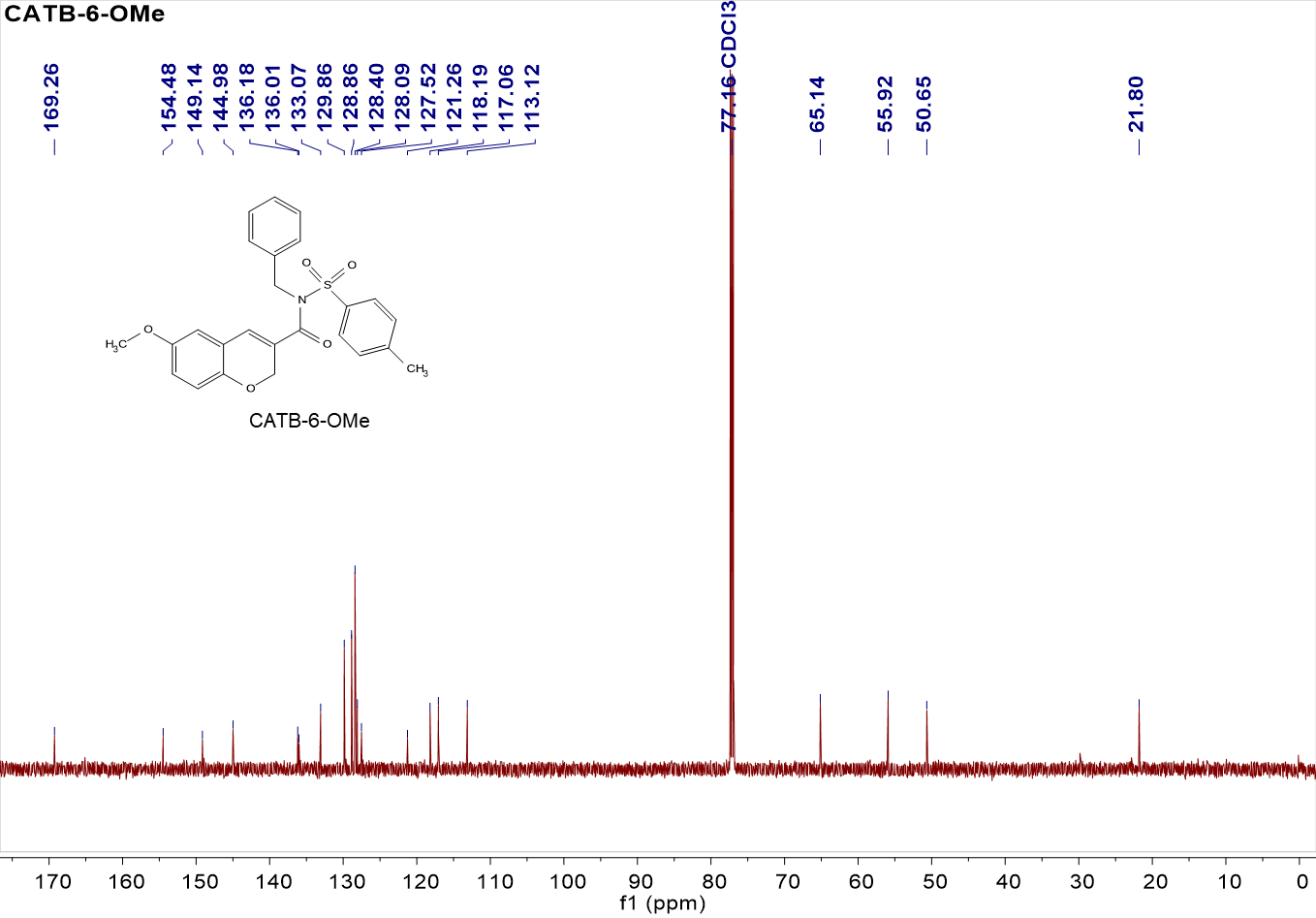


**Figure S38. ^13^C NMR spectrum of CATB-6-OMe.**


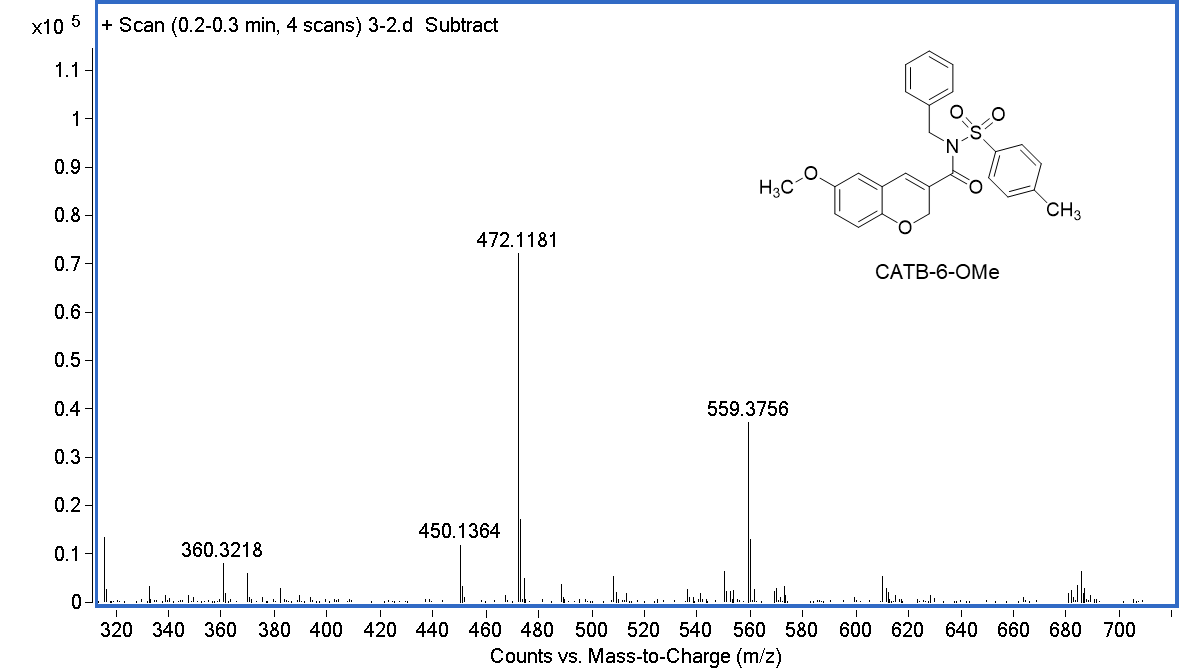


**Figure S39. High resolution mass spectrum of CATB-6-OMe.**


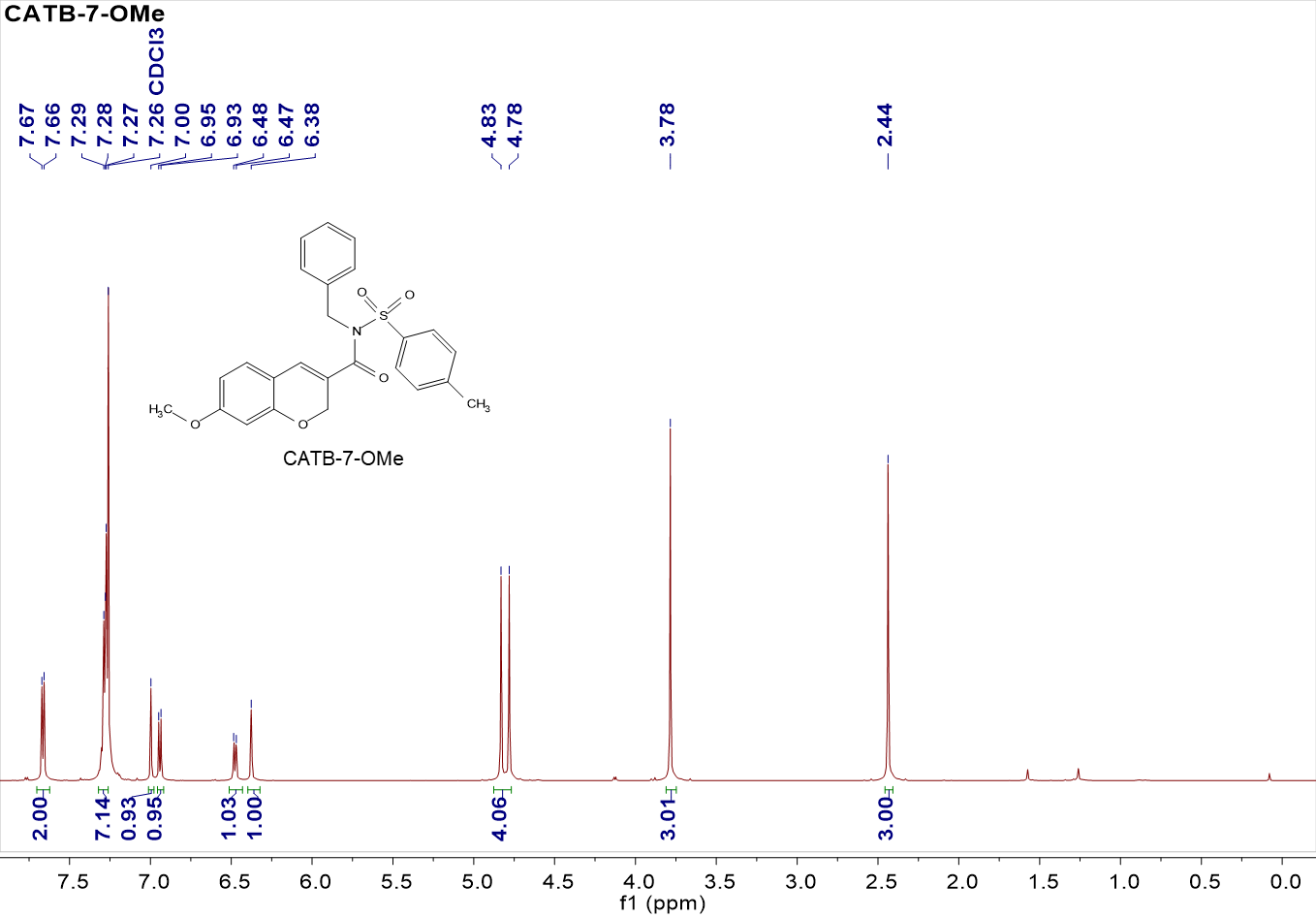


**Figure S40. ^1^H NMR spectrum of CATB-7-OMe.**


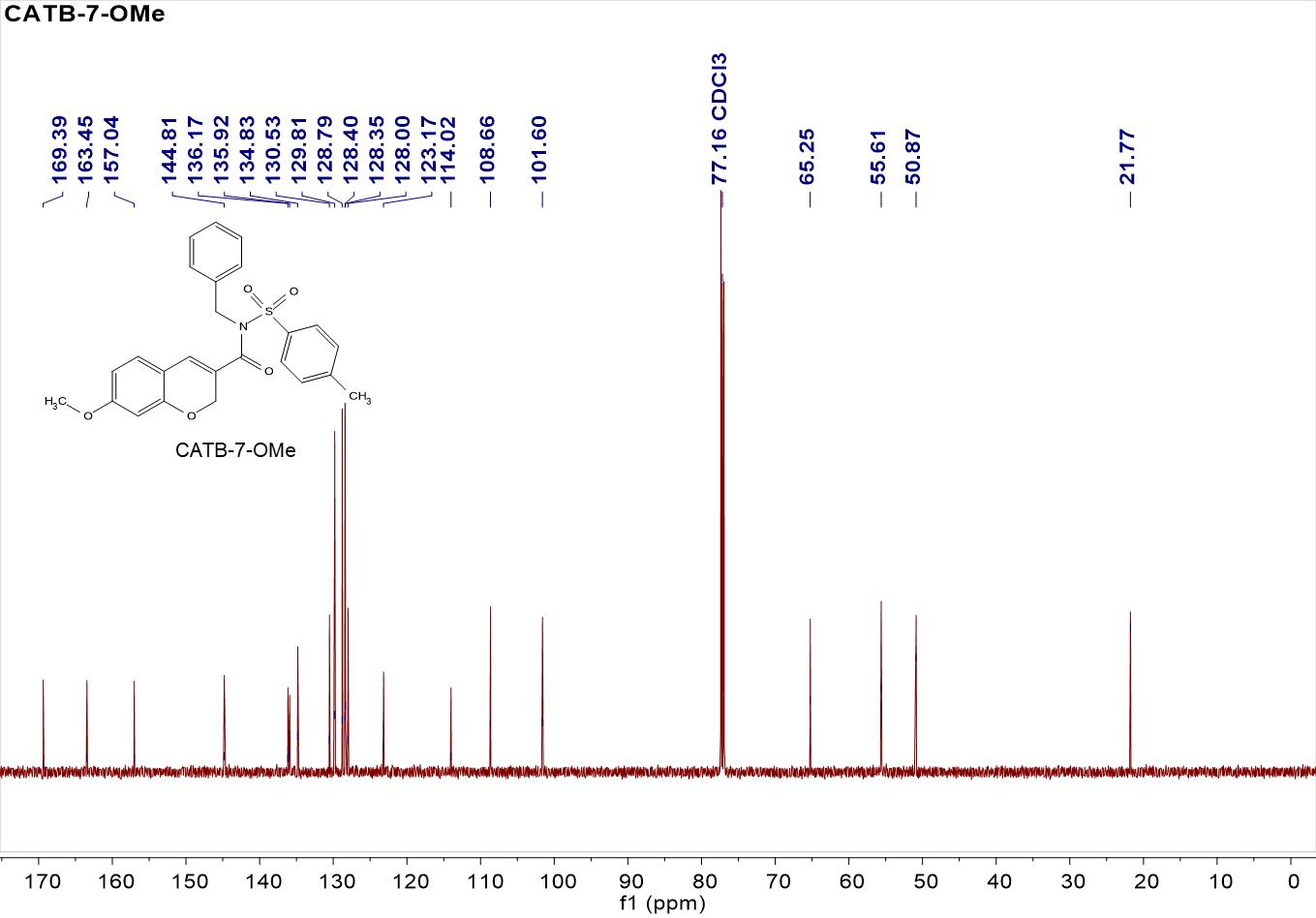


**Figure S41. ^13^C NMR spectrum of CATB-7-OMe.**


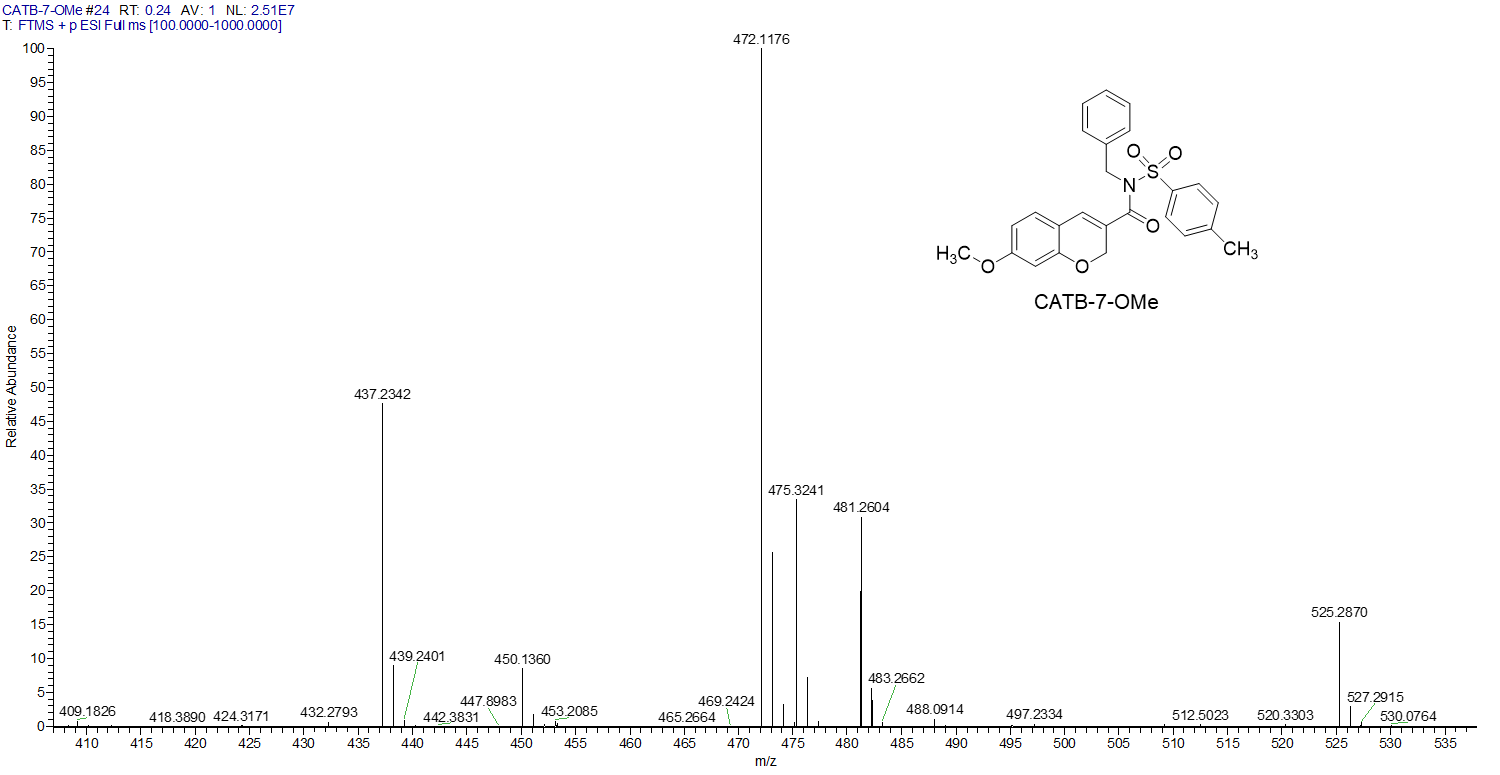


**Figure S42. High-resolution mass spectrum of CATB-7-OMe.**


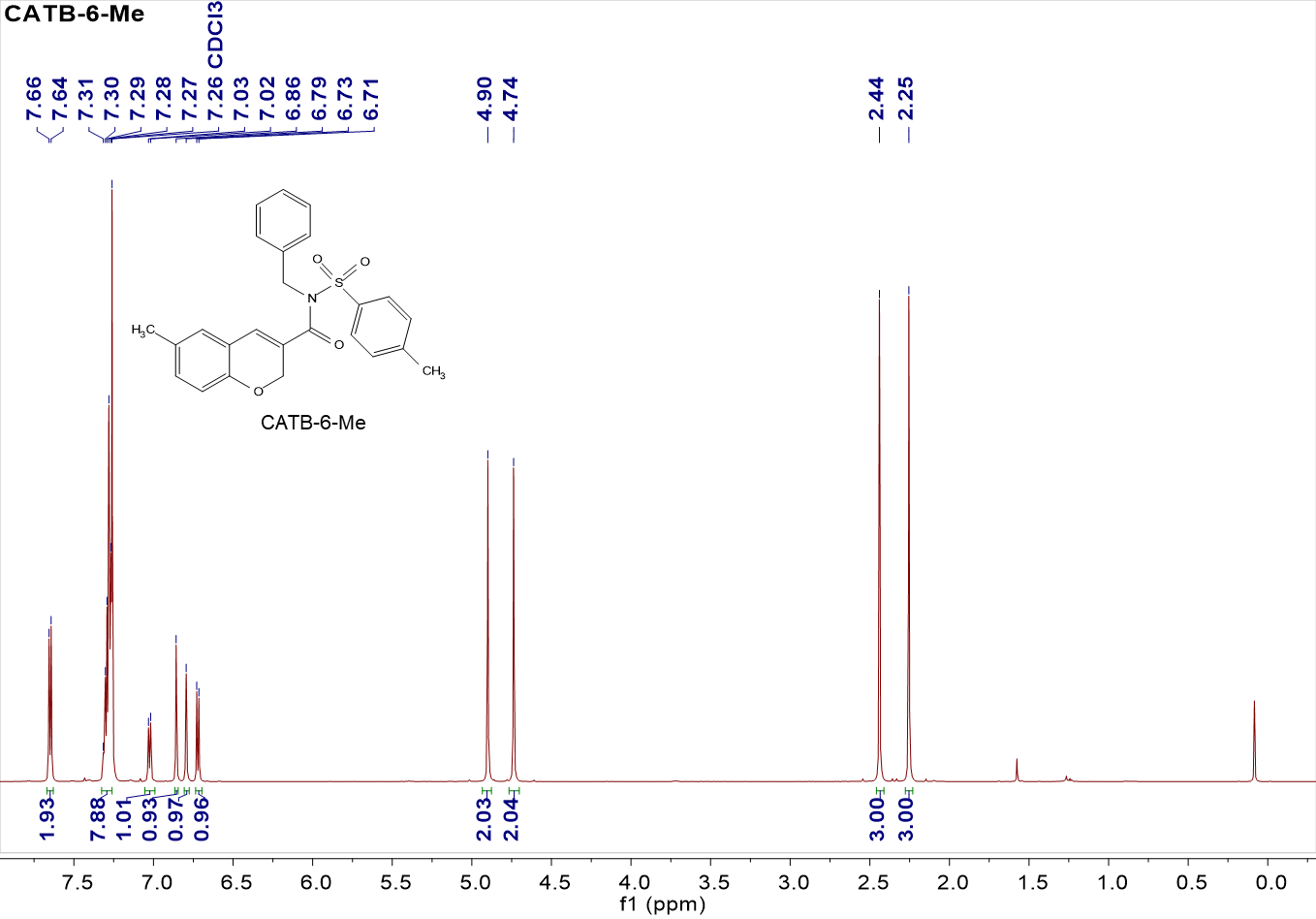


**Figure S43. ^1^H NMR spectrum of CATB-6-Me.**


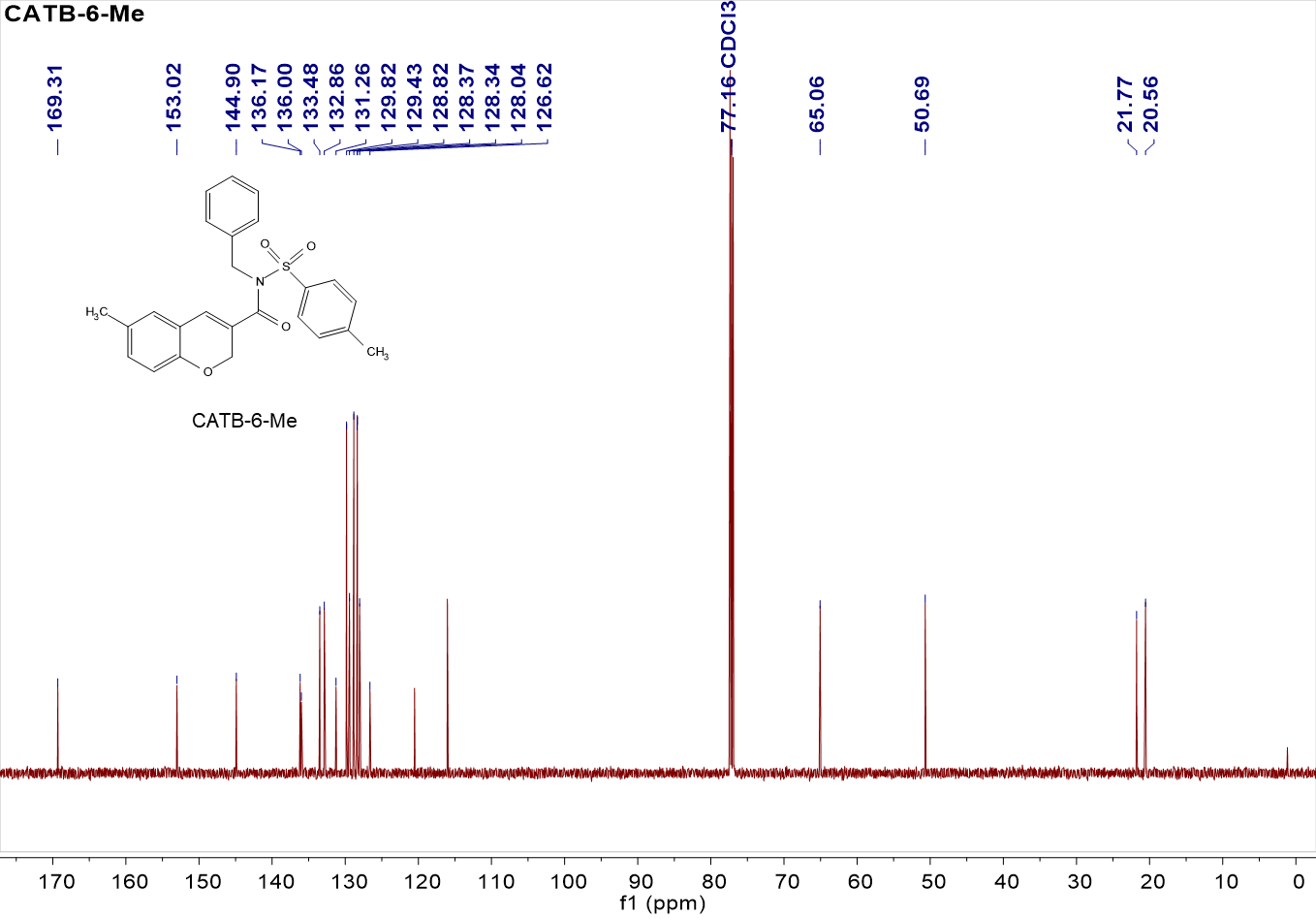


**Figure S44. ^13^C NMR spectrum of CATB-6-Me.**


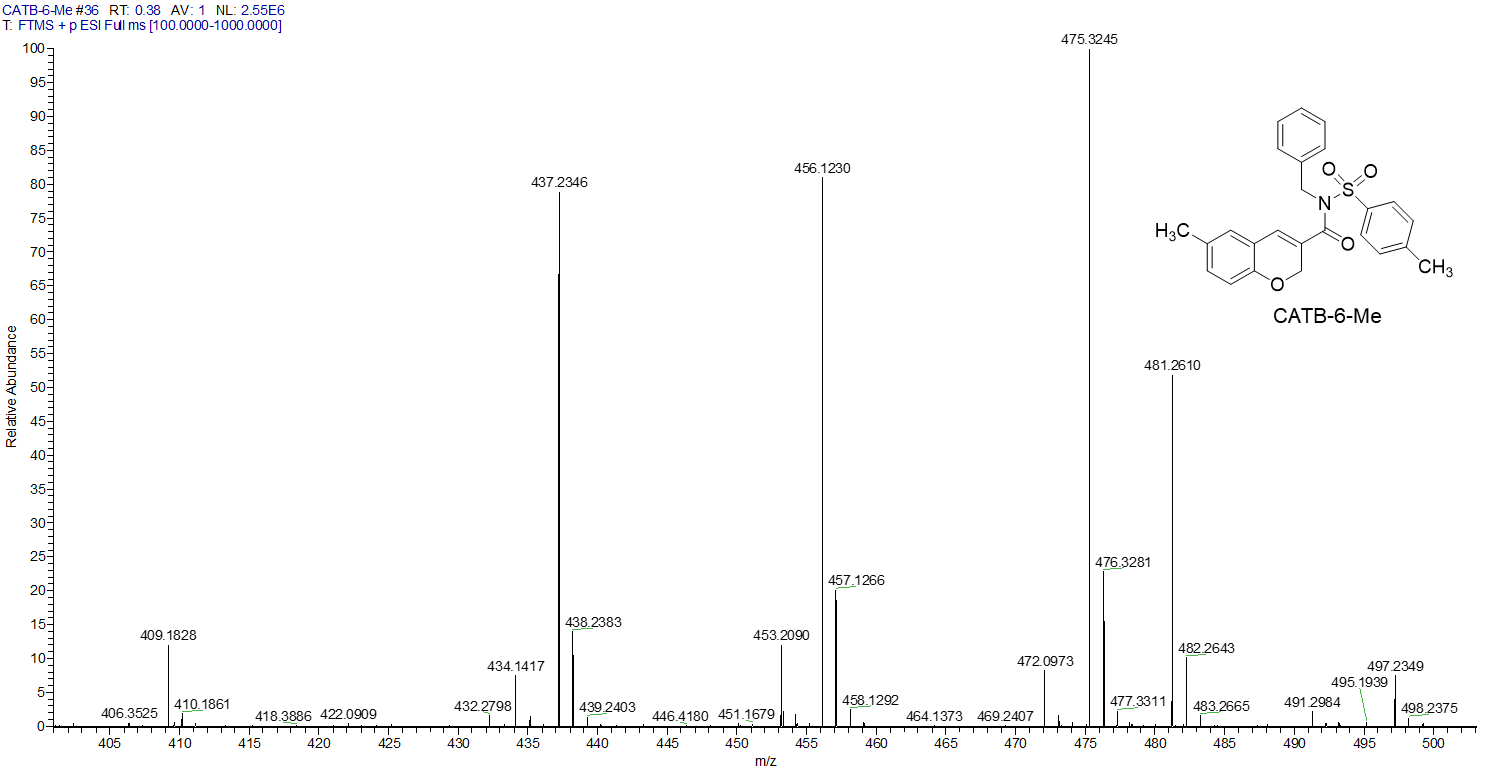


**Figure S45.** **High-resolution mass spectrum of CATB-6-Me.**


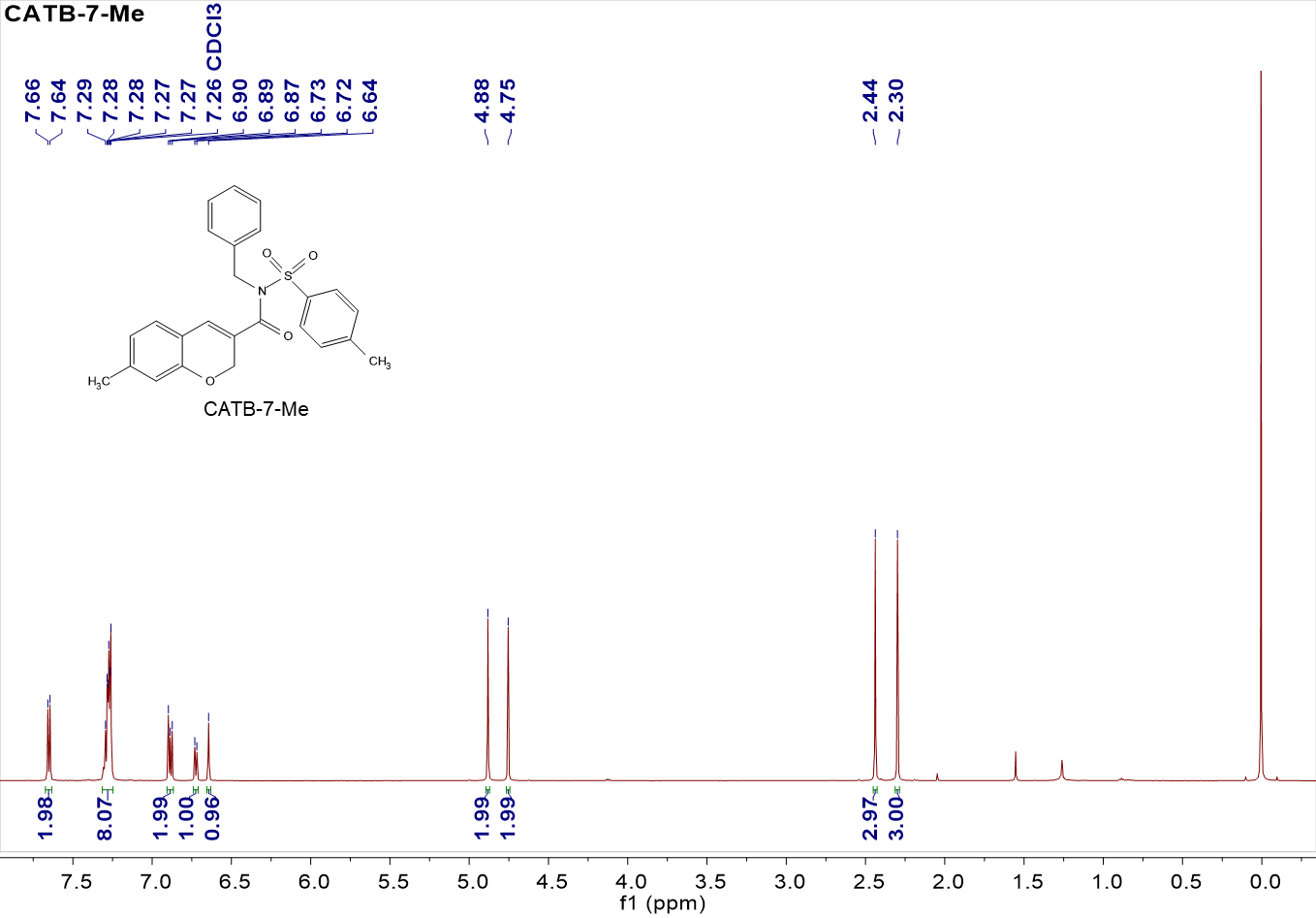


**Figure S46. ^1^H NMR spectrum of CATB-7-Me.**


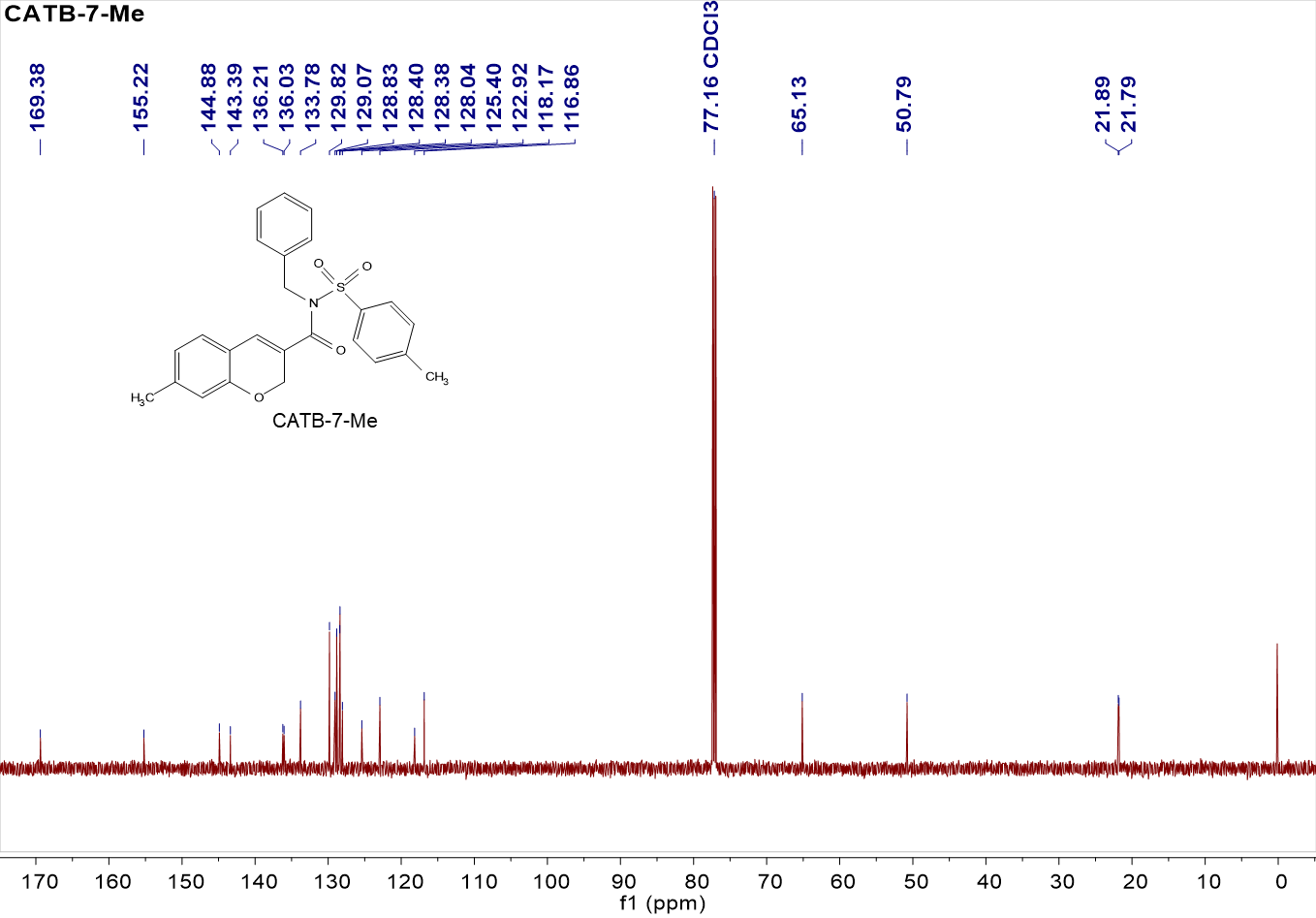


**Figure S47. ^13^C NMR spectrum of CATB-7-Me.**


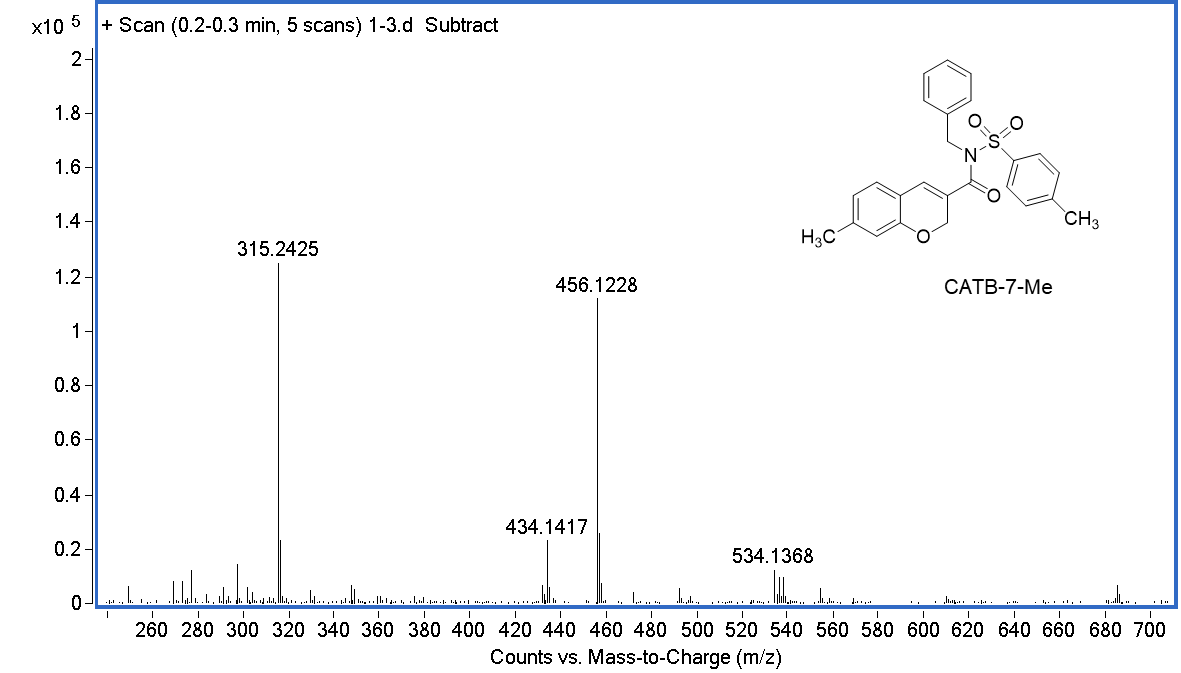


**Figure S48.** **High-resolution mass spectrum of CATB-7-Me.**


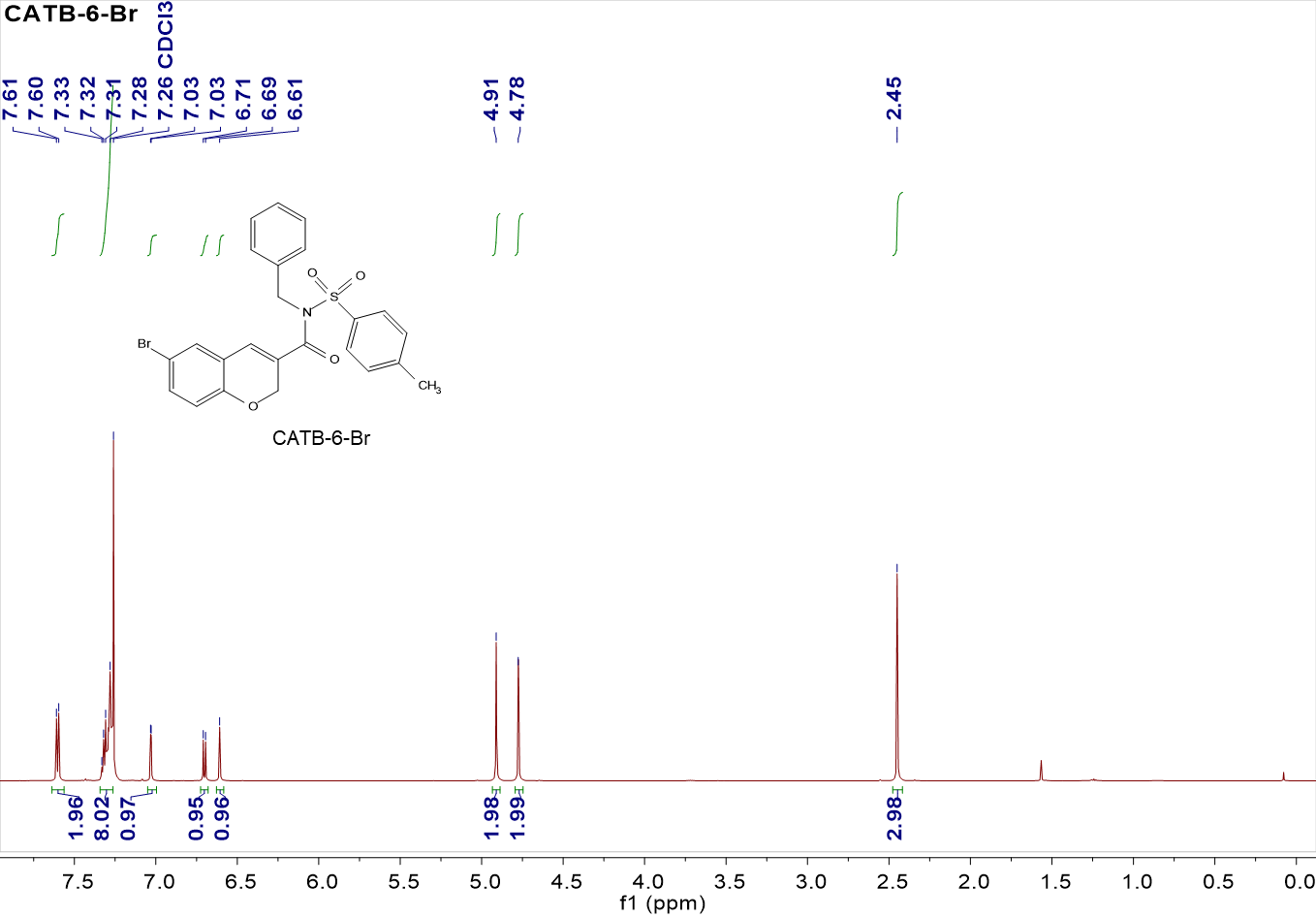


**Figure S49. ^1^H NMR spectrum of CATB-6-Br.**


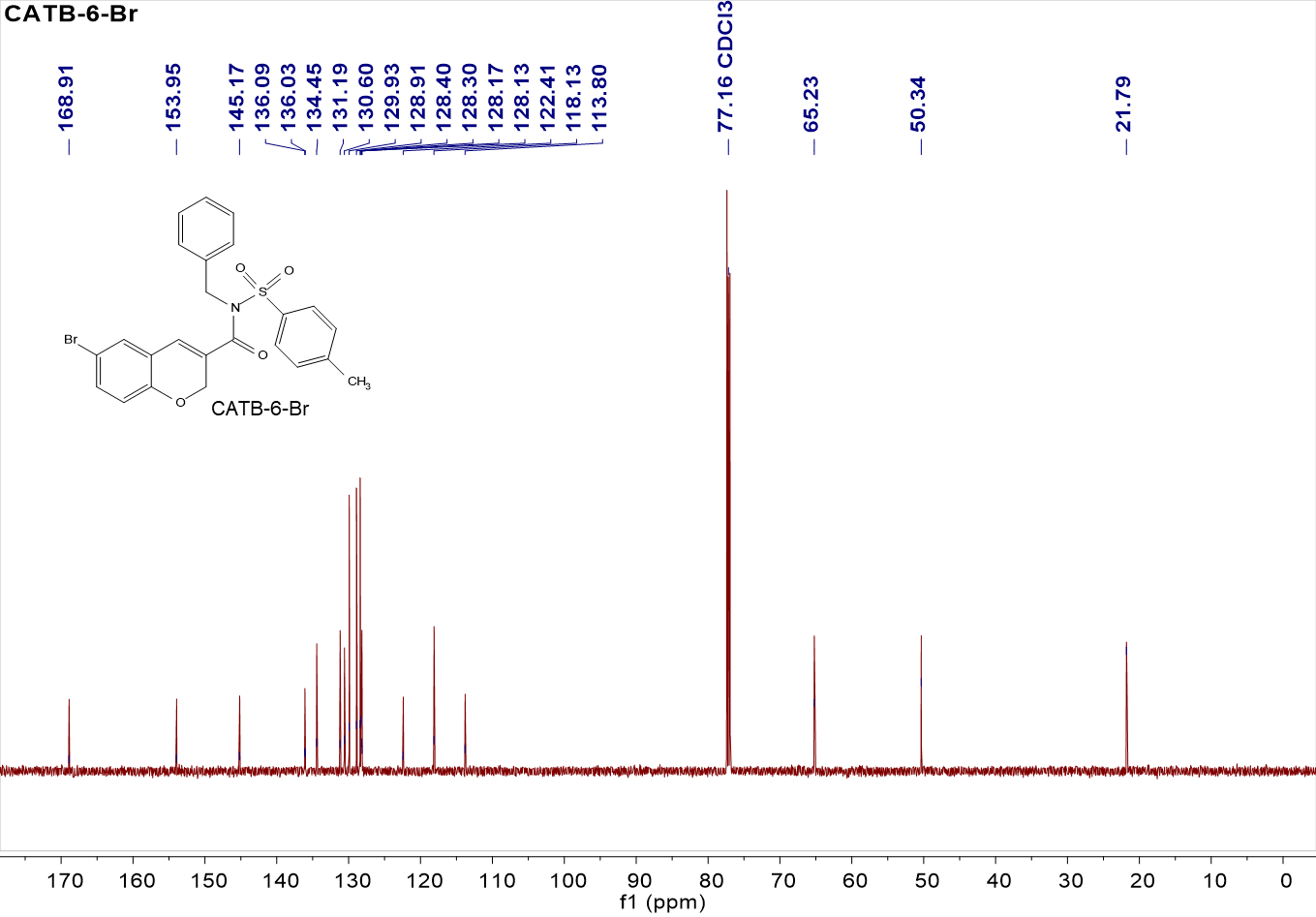


**Figure S50. ^13^C NMR spectrum of CATB-6-Br.**


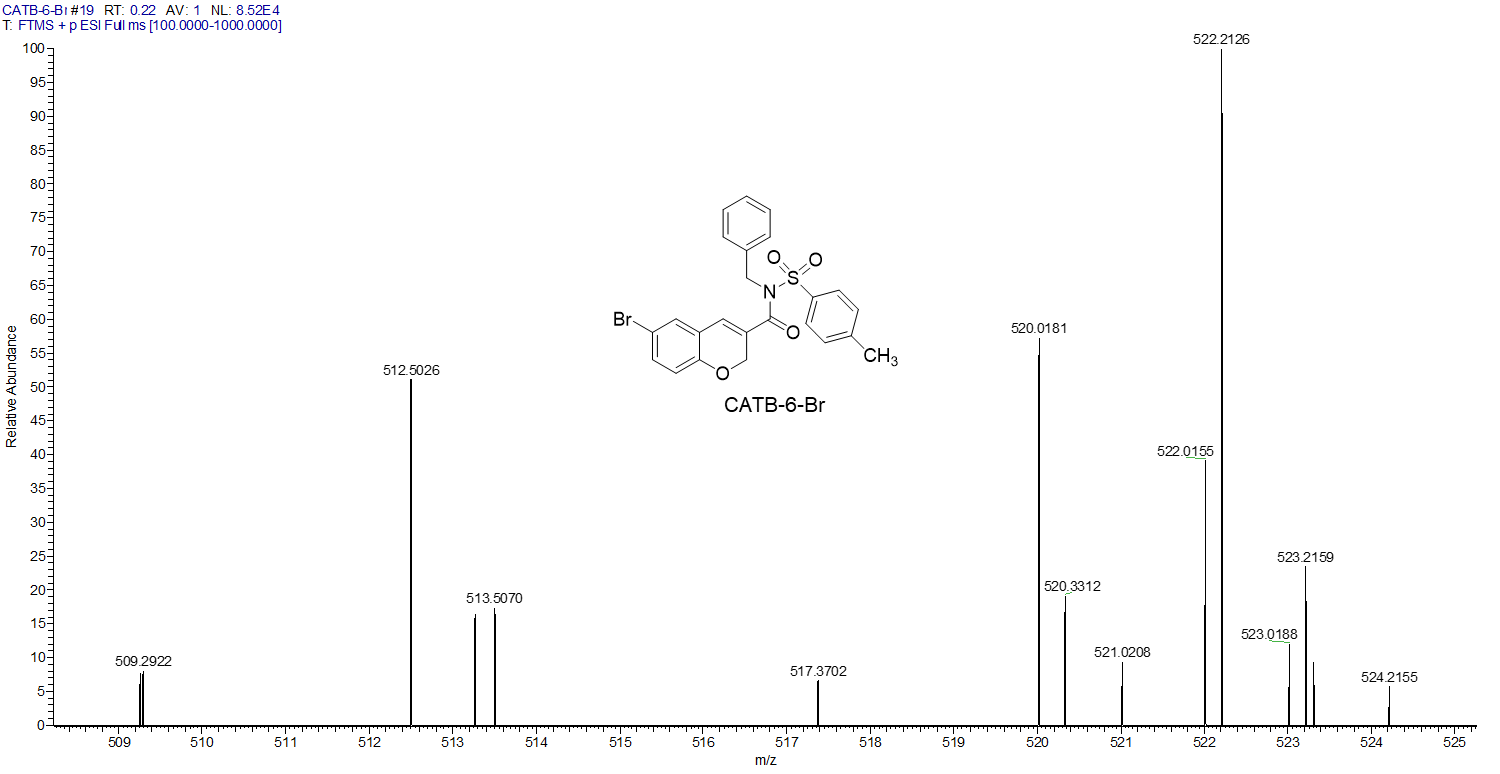


**Figure S51.** **High-resolution mass spectrum of CATB-6-Br.**


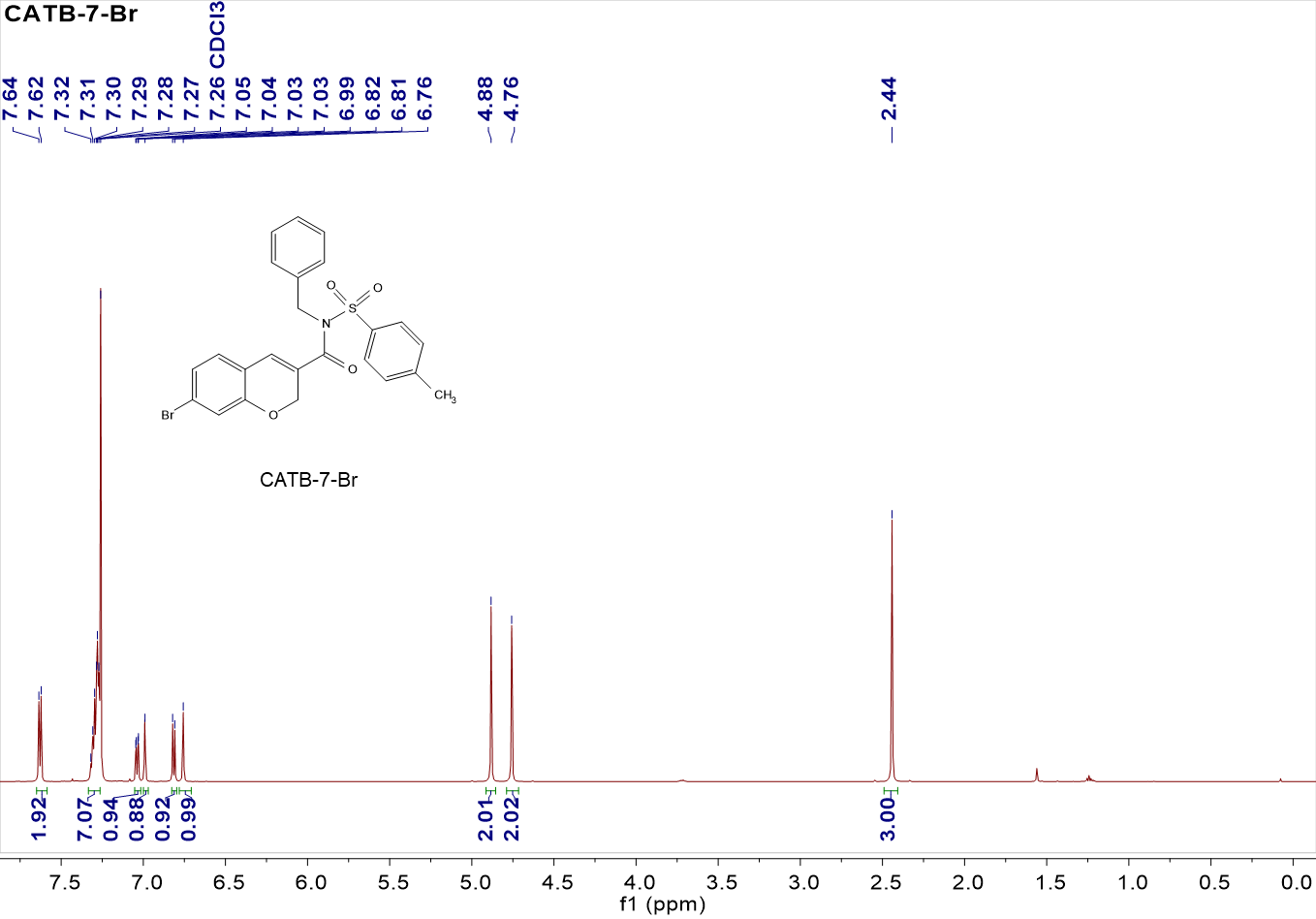


**Figure S52. ^1^H NMR spectrum of CATB-7-Br.**


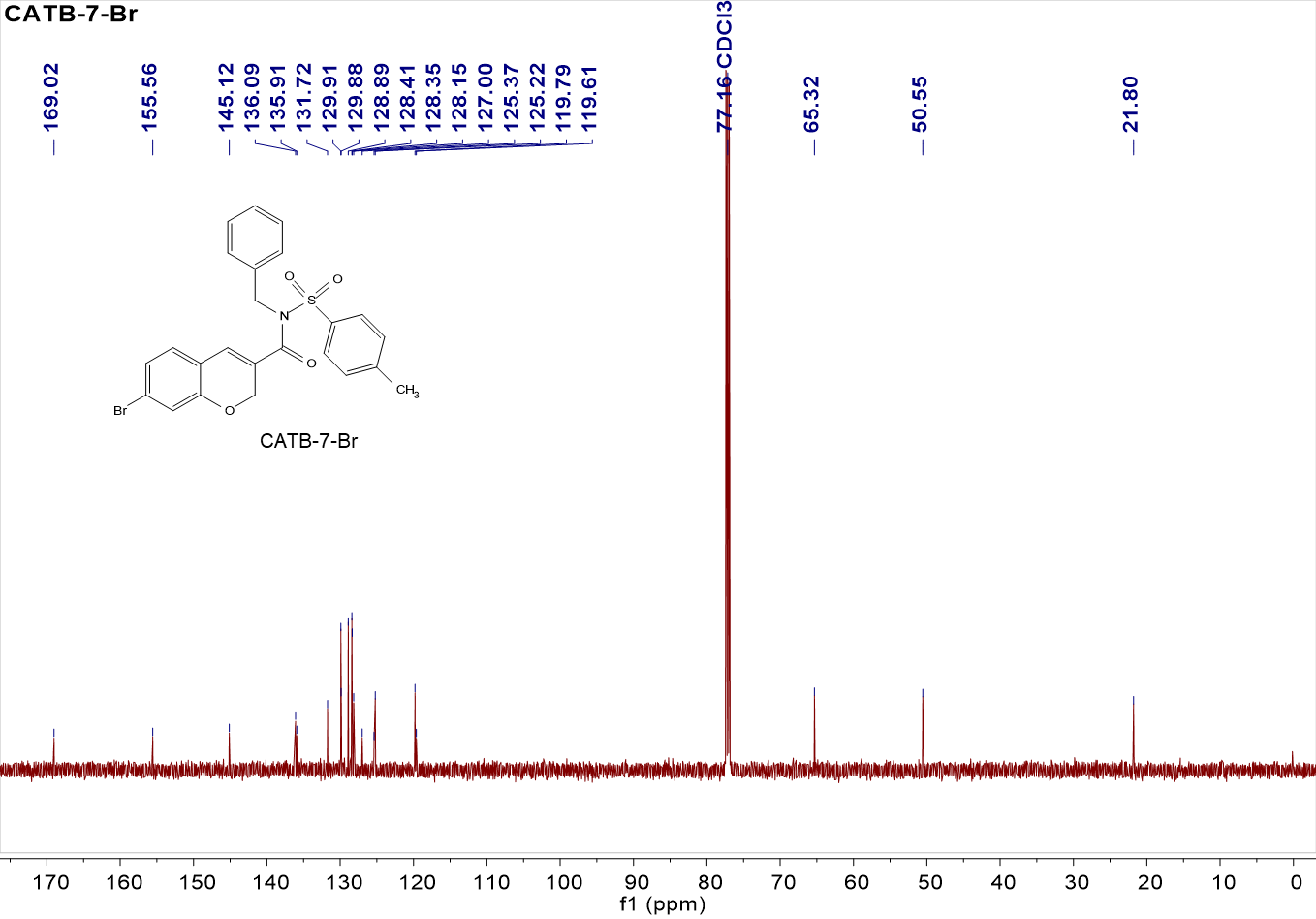


**Figure S53. ^13^C NMR spectrum of CATB-7-Br.**


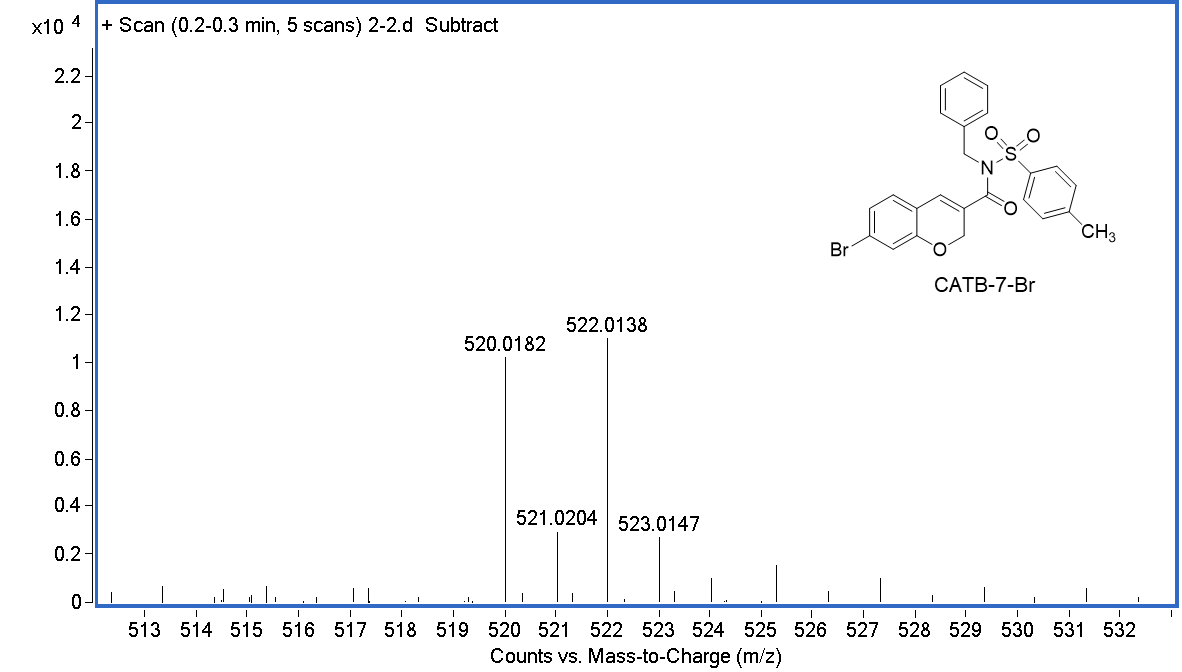


**Figure S54. High-resolution mass spectrum of CATB-7-Br.**


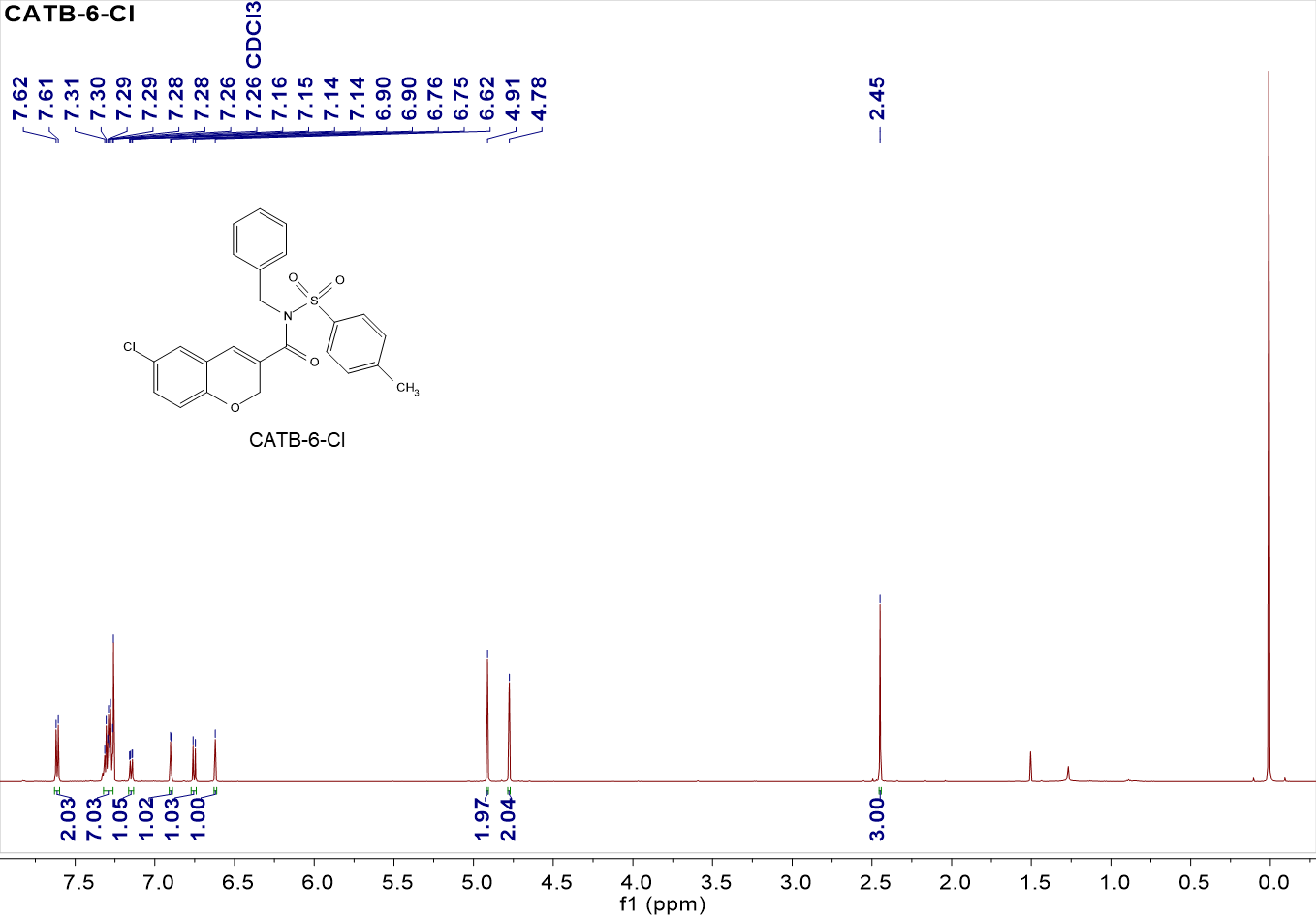


**Figure S55. ^1^H NMR spectrum of CATB-6-Cl.**


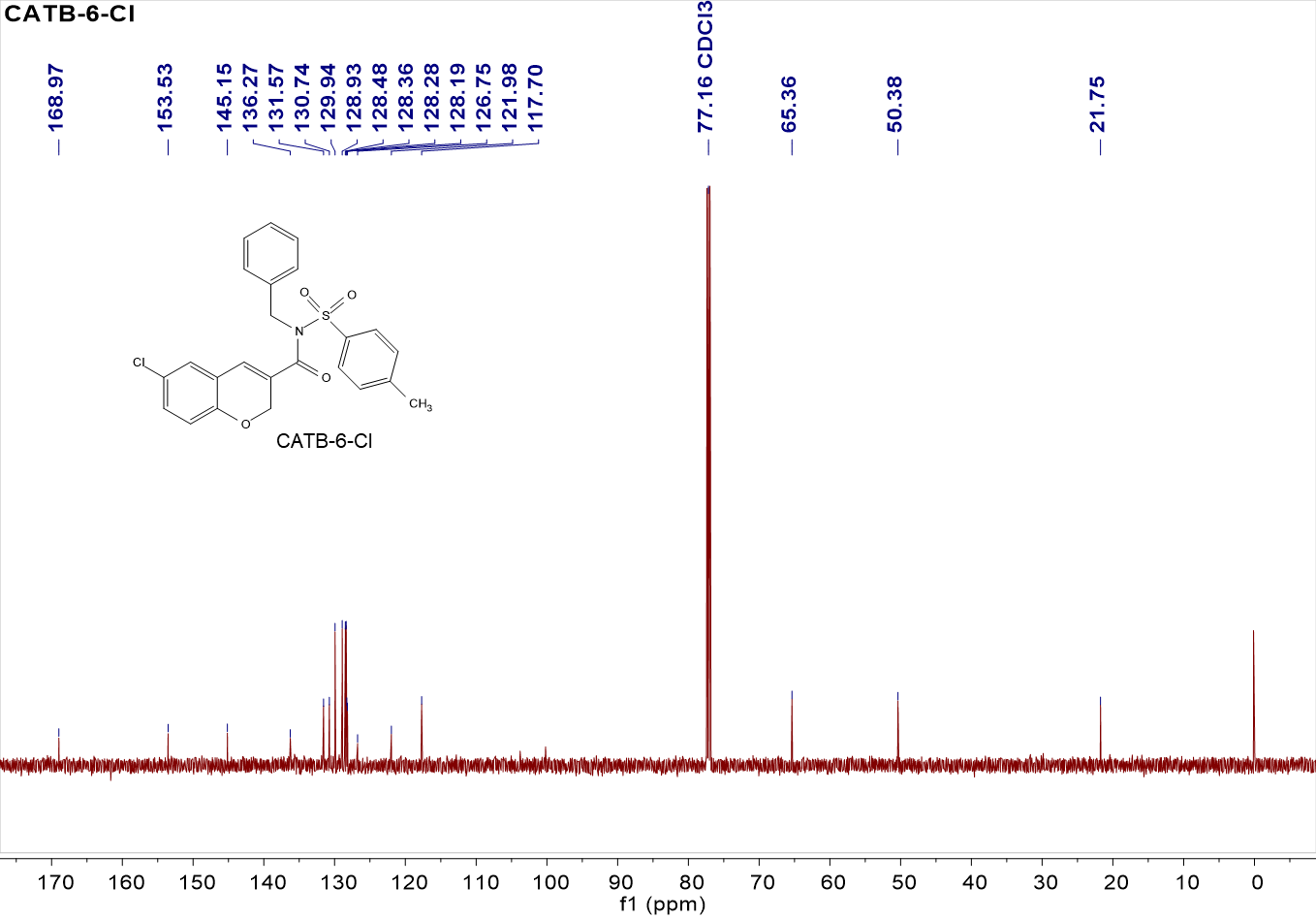


**Figure S56. ^13^C NMR spectrum of CATB-6-Cl.**


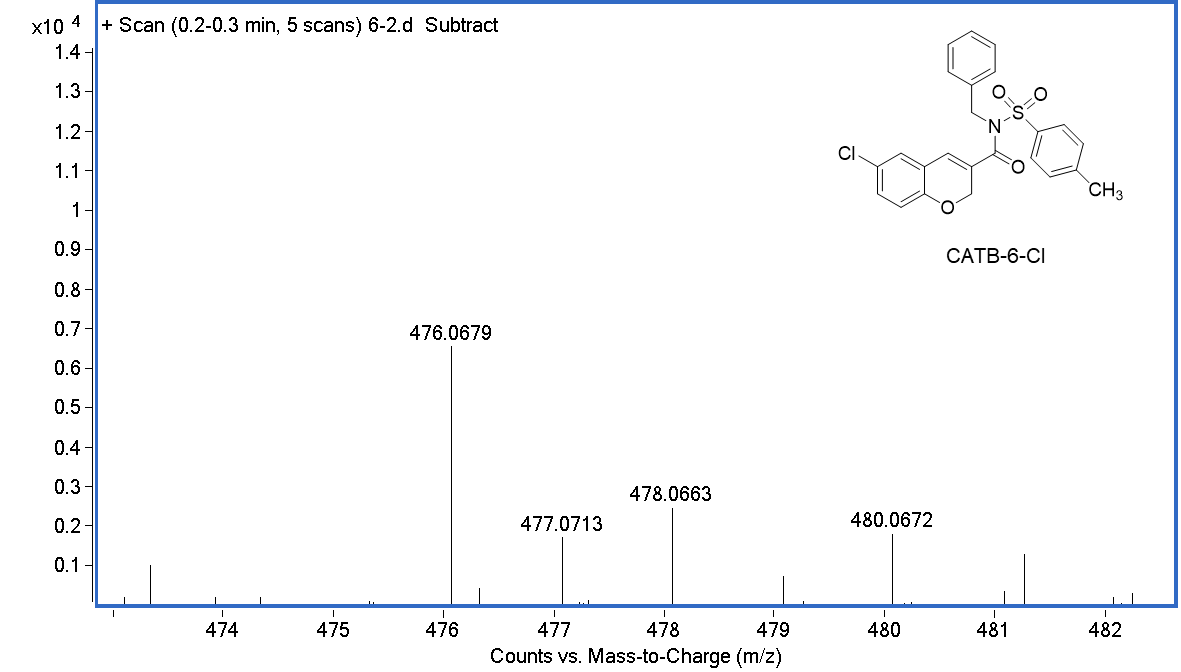


**Figure S57.** **High-resolution mass spectrum of CATB-6-Cl.**


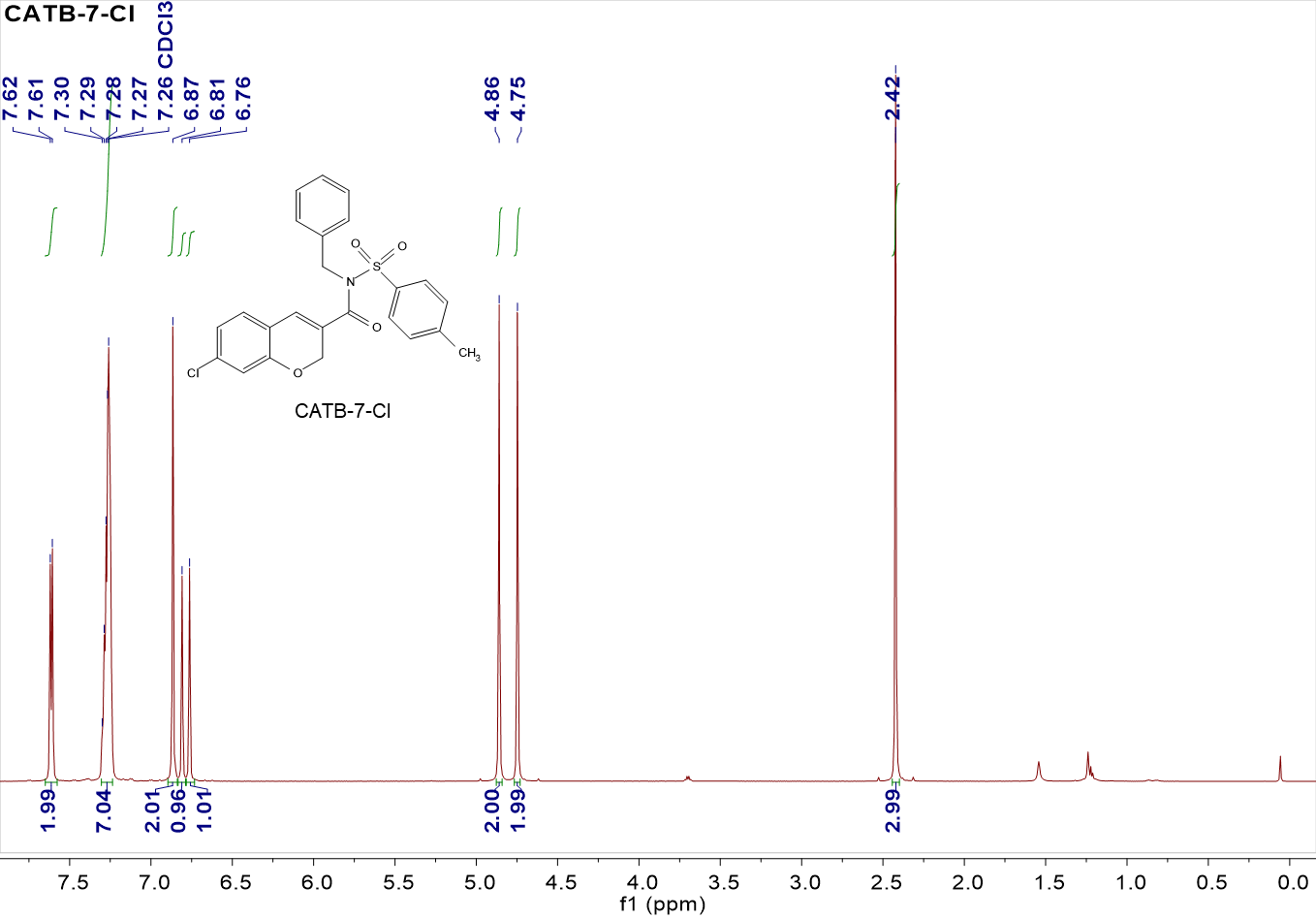


**Figure S58. ^1^H NMR spectrum of CATB-7-Cl.**


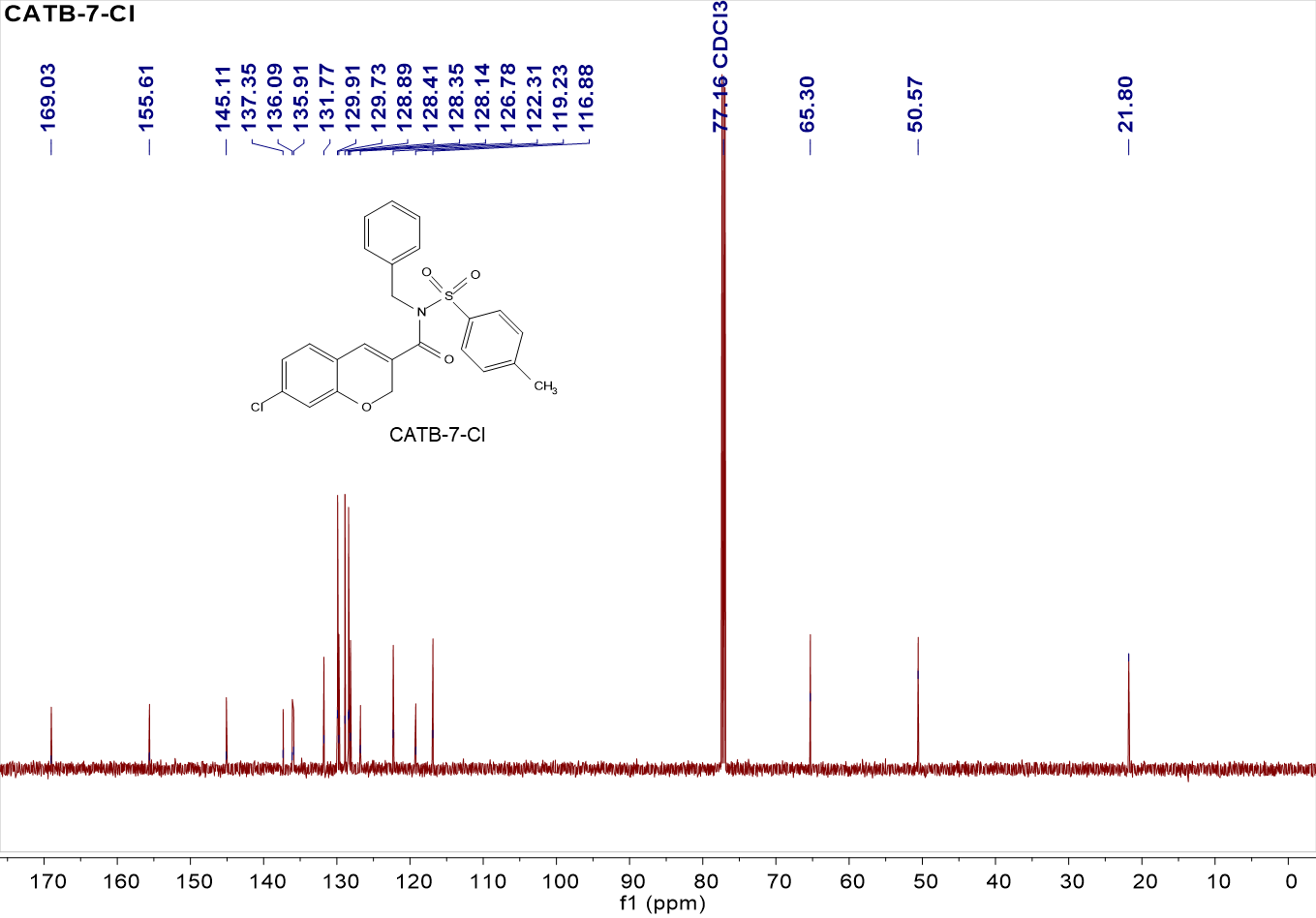


**Figure S59. ^13^C NMR spectrum of CATB-7-Cl**


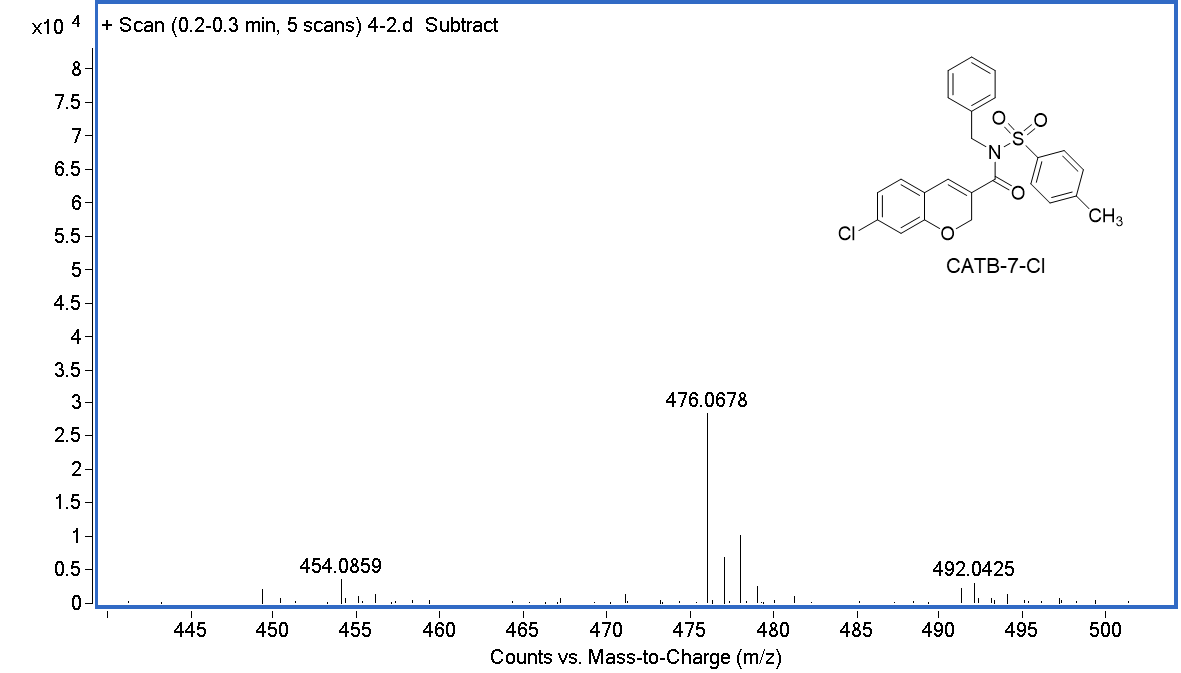


**Figure S60.** **High-resolution mass spectrum of CATB-7-Cl.**


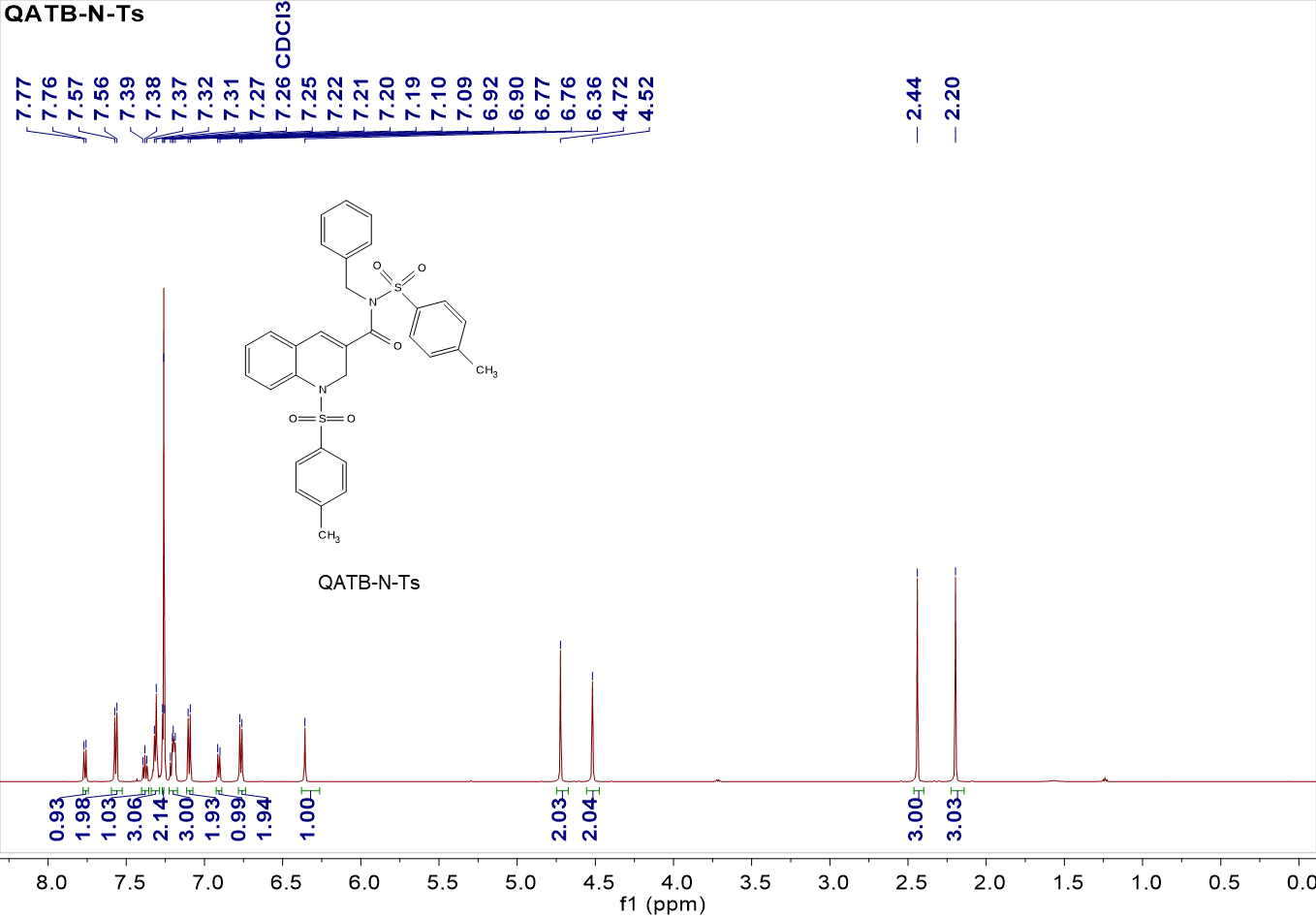


**Figure S61. ^1^H NMR spectrum of QATB-N-Ts.**


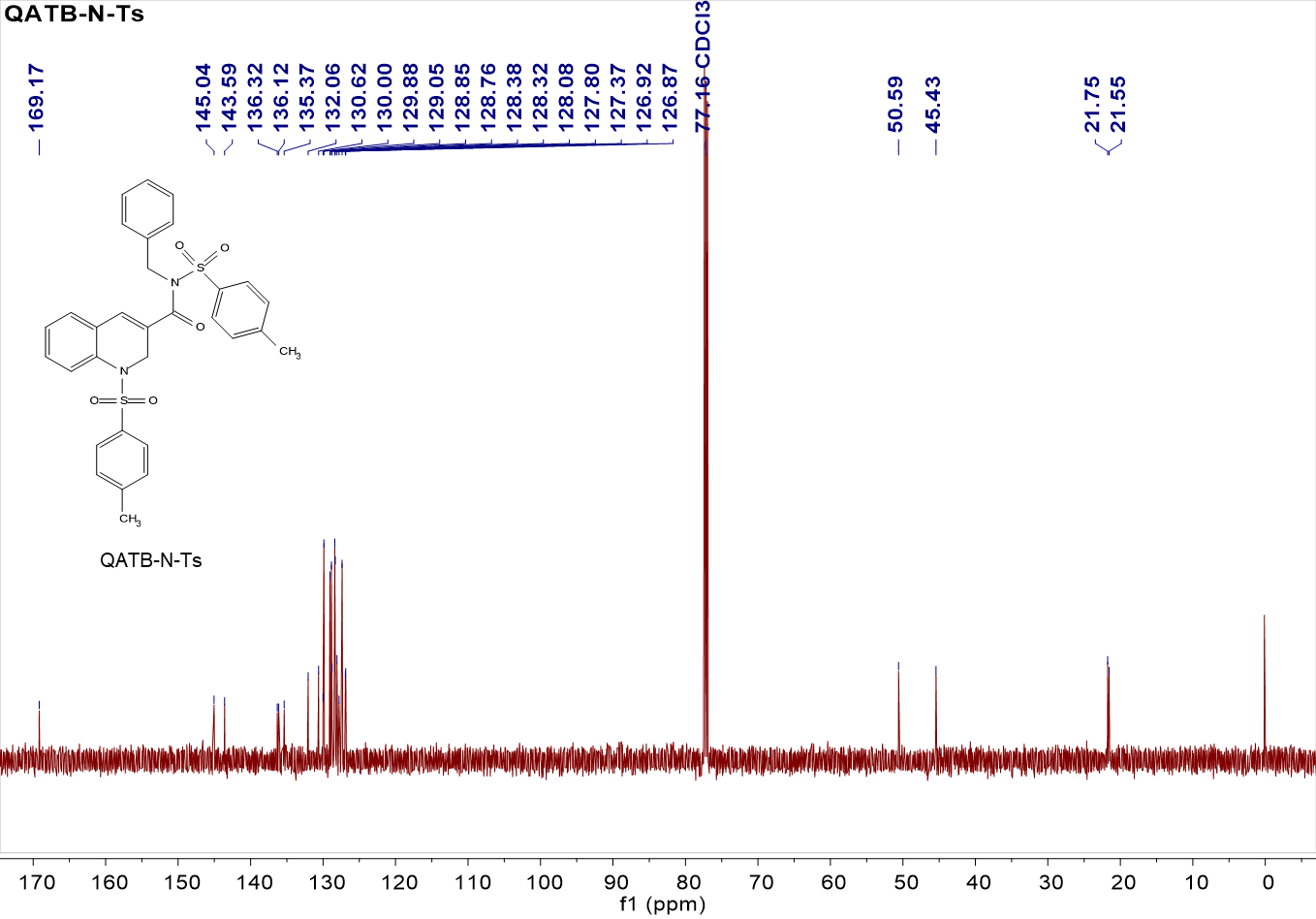


**Figure S62. ^13^C NMR spectrum of QATB-N-Ts.**


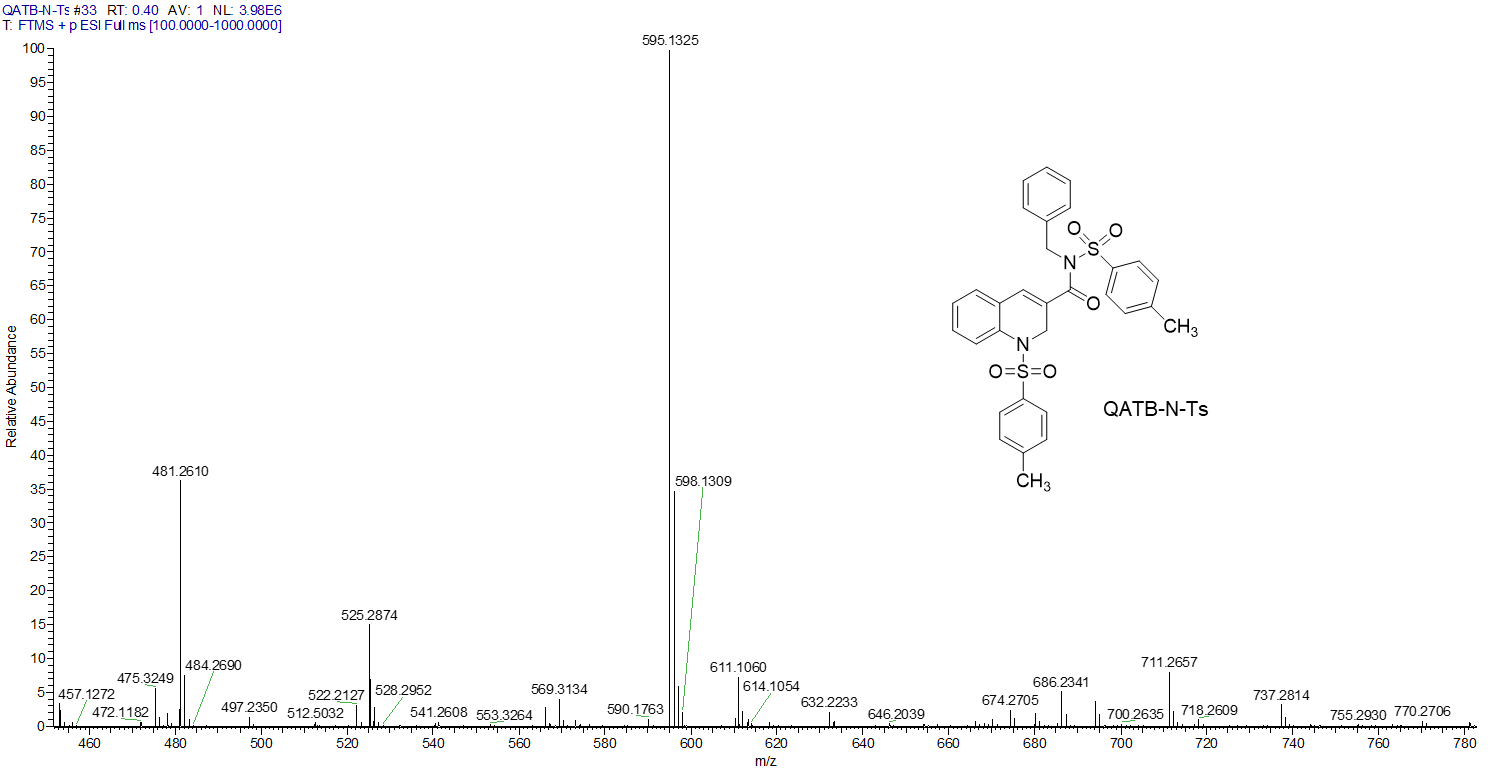


**Figure S63.** **High-resolution mass spectrum of QATB-N-Ts.**

**6. Cartesian coordinates of optimized molecular geometry**

**Table S3**. Cartesian coordinates of optimized CTAB in the ground state.

|  | X | | Y | | Z | |
| --- | --- | --- | --- | --- | --- | --- |
| S | | 2.113538 | | 0.908687 | | 1.173522 |
| O | | -2.653584 | | -3.206581 | | -0.025676 |
| O | | 1.057098 | | -1.829182 | | 0.940372 |
| O | | 2.006694 | | 0.428184 | | 2.545024 |
| O | | 2.181277 | | 2.343000 | | 0.867256 |
| N | | 0.690040 | | 0.372720 | | 0.334210 |
| C | | -3.701757 | | -2.334104 | | -0.055850 |
| C | | -4.987764 | | -2.849727 | | -0.213579 |
| H | | -5.110051 | | -3.918919 | | -0.348042 |
| C | | -6.079295 | | -1.981351 | | -0.200682 |
| H | | -7.079064 | | -2.383277 | | -0.336345 |
| C | | -5.899153 | | -0.604712 | | -0.021182 |
| C | | -4.616495 | | -0.097947 | | 0.164750 |
| H | | -4.454298 | | 0.965986 | | 0.314619 |
| C | | -3.503022 | | -0.951526 | | 0.150213 |
| C | | -2.150477 | | -0.492722 | | 0.403446 |
| H | | -2.007613 | | 0.489134 | | 0.837877 |
| C | | -1.104202 | | -1.304584 | | 0.151968 |
| C | | -1.372236 | | -2.683031 | | -0.409644 |
| H | | -1.316465 | | -2.663357 | | -1.511066 |
| H | | -0.631938 | | -3.392306 | | -0.037643 |
| C | | 0.294152 | | -0.976790 | | 0.510246 |
| C | | 3.501824 | | 0.130013 | | 0.357552 |
| C | | 3.931022 | | -1.133863 | | 0.767818 |
| H | | 3.411252 | | -1.644070 | | 1.567844 |
| C | | 5.014680 | | -1.714398 | | 0.116213 |
| H | | 5.355692 | | -2.698062 | | 0.427148 |
| C | | 5.673454 | | -1.056484 | | -0.933536 |
| C | | 5.222471 | | 0.213602 | | -1.317010 |
| H | | 5.727130 | | 0.741400 | | -2.121482 |
| C | | 4.143338 | | 0.817275 | | -0.674949 |
| H | | 3.814484 | | 1.813095 | | -0.949490 |
| C | | 6.864368 | | -1.691807 | | -1.608017 |
| H | | 7.777451 | | -1.517996 | | -1.025741 |
| H | | 7.028751 | | -1.279823 | | -2.607495 |
| H | | 6.739940 | | -2.774948 | | -1.699926 |
| C | | 0.297771 | | 1.128629 | | -0.886914 |
| H | | 0.063114 | | 0.399648 | | -1.666520 |
| H | | 1.182808 | | 1.677583 | | -1.218420 |
| C | | -0.861433 | | 2.088974 | | -0.717936 |
| C | | -0.903502 | | 3.003622 | | 0.341850 |
| H | | -0.080233 | | 3.041279 | | 1.046093 |
| C | | -1.999691 | | 3.853208 | | 0.490099 |
| H | | -2.026375 | | 4.553289 | | 1.319959 |
| C | | -3.058571 | | 3.809233 | | -0.421269 |
| H | | -3.911101 | | 4.471048 | | -0.300063 |
| C | | -3.013508 | | 2.911270 | | -1.488503 |
| H | | -3.832198 | | 2.865010 | | -2.200465 |
| C | | -1.920375 | | 2.054924 | | -1.631481 |
| H | | -1.904291 | | 1.335669 | | -2.446399 |
| H | | -6.755372 | | 0.061976 | | -0.017693 |

**Table S4**. Cartesian coordinates of optimized CTAB-6-OMe in the ground state.

|  | X | | Y | | Z | |
| --- | --- | --- | --- | --- | --- | --- |
| S | | 2.530517 | | 0.893894 | | 1.210483 |
| O | | -6.517714 | | -0.066439 | | -0.084627 |
| O | | -2.081557 | | -3.375364 | | -0.025984 |
| O | | 1.625473 | | -1.902240 | | 0.888765 |
| O | | 2.453103 | | 0.360431 | | 2.564381 |
| O | | 2.510069 | | 2.339505 | | 0.953255 |
| N | | 1.143919 | | 0.300939 | | 0.354316 |
| C | | -3.147184 | | -2.517370 | | -0.064441 |
| C | | -4.428255 | | -3.040615 | | -0.249624 |
| H | | -4.544179 | | -4.108210 | | -0.400847 |
| C | | -5.528030 | | -2.190562 | | -0.245229 |
| H | | -6.530130 | | -2.574955 | | -0.402707 |
| C | | -5.368838 | | -0.807996 | | -0.046166 |
| C | | -4.095218 | | -0.286912 | | 0.173417 |
| H | | -3.930059 | | 0.771368 | | 0.336208 |
| C | | -2.978172 | | -1.140074 | | 0.164471 |
| C | | -1.637292 | | -0.660560 | | 0.432231 |
| H | | -1.512923 | | 0.316110 | | 0.883087 |
| C | | -0.574614 | | -1.444995 | | 0.161603 |
| C | | -0.818578 | | -2.820192 | | -0.420762 |
| H | | -0.777182 | | -2.778182 | | -1.522809 |
| H | | -0.056688 | | -3.518263 | | -0.071457 |
| C | | 0.814799 | | -1.074432 | | 0.501508 |
| C | | 3.965197 | | 0.231318 | | 0.372197 |
| C | | 4.460480 | | -1.025050 | | 0.727898 |
| H | | 3.964720 | | -1.599383 | | 1.499210 |
| C | | 5.577904 | | -1.515299 | | 0.059423 |
| H | | 5.970256 | | -2.492303 | | 0.327931 |
| C | | 6.206133 | | -0.774947 | | -0.953576 |
| C | | 5.688751 | | 0.485026 | | -1.282417 |
| H | | 6.168505 | | 1.075923 | | -2.057733 |
| C | | 4.574362 | | 0.998978 | | -0.622570 |
| H | | 4.193083 | | 1.987028 | | -0.854022 |
| C | | 7.433920 | | -1.313022 | | -1.646318 |
| H | | 8.332600 | | -1.117711 | | -1.048665 |
| H | | 7.581928 | | -0.846553 | | -2.624180 |
| H | | 7.368960 | | -2.395818 | | -1.789393 |
| C | | 0.736382 | | 1.045351 | | -0.869498 |
| H | | 0.557097 | | 0.312256 | | -1.659991 |
| H | | 1.597145 | | 1.643597 | | -1.179392 |
| C | | -0.475685 | | 1.942557 | | -0.719985 |
| C | | -0.586590 | | 2.849485 | | 0.341921 |
| H | | 0.219926 | | 2.925576 | | 1.062437 |
| C | | -1.728956 | | 3.639116 | | 0.471958 |
| H | | -1.808530 | | 4.333135 | | 1.303538 |
| C | | -2.765637 | | 3.545029 | | -0.461472 |
| H | | -3.652696 | | 4.163113 | | -0.356553 |
| C | | -2.652002 | | 2.655867 | | -1.530984 |
| H | | -3.451312 | | 2.571725 | | -2.261482 |
| C | | -1.513484 | | 1.857121 | | -1.654016 |
| H | | -1.444408 | | 1.143006 | | -2.470545 |
| C | | -6.404915 | | 1.333224 | | 0.120126 |
| H | | -7.416154 | | 1.734154 | | 0.036558 |
| H | | -5.763488 | | 1.804522 | | -0.637132 |
| H | | -6.004039 | | 1.567566 | | 1.115511 |

**Table S5**. Cartesian coordinates of optimized CTAB-6-OMe in the excited state.

|  | X | | Y | | Z | |
| --- | --- | --- | --- | --- | --- | --- |
| S | | 2.370968 | | 0.418486 | | 1.355625 |
| O | | -6.421431 | | 0.419088 | | 0.255171 |
| O | | -2.444137 | | -3.239396 | | -0.456606 |
| O | | 1.606205 | | -2.222571 | | -0.129445 |
| O | | 2.228503 | | -0.532742 | | 2.455942 |
| O | | 2.290864 | | 1.871466 | | 1.579149 |
| N | | 1.133129 | | 0.062447 | | 0.256451 |
| C | | -3.377425 | | -2.304344 | | -0.216805 |
| C | | -4.712733 | | -2.659911 | | -0.481673 |
| H | | -4.912801 | | -3.665972 | | -0.834055 |
| C | | -5.735215 | | -1.738155 | | -0.309125 |
| H | | -6.771141 | | -1.978531 | | -0.512501 |
| C | | -5.399831 | | -0.444017 | | 0.129789 |
| C | | -4.045789 | | -0.092427 | | 0.416141 |
| H | | -3.791624 | | 0.910026 | | 0.737239 |
| C | | -3.024116 | | -1.009210 | | 0.268300 |
| C | | -1.630410 | | -0.694480 | | 0.514759 |
| H | | -1.371001 | | 0.275662 | | 0.908014 |
| C | | -0.658279 | | -1.584457 | | 0.110773 |
| C | | -1.034428 | | -2.968131 | | -0.312717 |
| H | | -0.581035 | | -3.235204 | | -1.275343 |
| H | | -0.663245 | | -3.707013 | | 0.409998 |
| C | | 0.757904 | | -1.336761 | | 0.071892 |
| C | | 3.938038 | | 0.129207 | | 0.526698 |
| C | | 4.402568 | | -1.177971 | | 0.359264 |
| H | | 3.803566 | | -2.009266 | | 0.707891 |
| C | | 5.616647 | | -1.378916 | | -0.288706 |
| H | | 5.983841 | | -2.392681 | | -0.425783 |
| C | | 6.374881 | | -0.300928 | | -0.772185 |
| C | | 5.883982 | | 0.997072 | | -0.589043 |
| H | | 6.458703 | | 1.844414 | | -0.953342 |
| C | | 4.670624 | | 1.221489 | | 0.061414 |
| H | | 4.297925 | | 2.226213 | | 0.224947 |
| C | | 7.702317 | | -0.543053 | | -1.448701 |
| H | | 8.484948 | | -0.750478 | | -0.708732 |
| H | | 8.022154 | | 0.324883 | | -2.032029 |
| H | | 7.655708 | | -1.406830 | | -2.119514 |
| C | | 0.981813 | | 0.974919 | | -0.904458 |
| H | | 1.017234 | | 0.374596 | | -1.818899 |
| H | | 1.852378 | | 1.637875 | | -0.928019 |
| C | | -0.282776 | | 1.811480 | | -0.869540 |
| C | | -0.504532 | | 2.715796 | | 0.178974 |
| H | | 0.244887 | | 2.808754 | | 0.958577 |
| C | | -1.684302 | | 3.457874 | | 0.228718 |
| H | | -1.845865 | | 4.155478 | | 1.045873 |
| C | | -2.651815 | | 3.316362 | | -0.772089 |
| H | | -3.561268 | | 3.911217 | | -0.740859 |
| C | | -2.432507 | | 2.423149 | | -1.822547 |
| H | | -3.177280 | | 2.304228 | | -2.604460 |
| C | | -1.253415 | | 1.676001 | | -1.866428 |
| H | | -1.094425 | | 0.965959 | | -2.673960 |
| C | | -6.170505 | | 1.770994 | | 0.659764 |
| H | | -7.139551 | | 2.267799 | | 0.629657 |
| H | | -5.474575 | | 2.264545 | | -0.026181 |
| H | | -5.769142 | | 1.805168 | | 1.677808 |

**Table S6**. Cartesian coordinates of optimized CTAB-6-Me in the ground state.

|  | X | | Y | | Z | |
| --- | --- | --- | --- | --- | --- | --- |
| S | | -2.409156 | | 1.285792 | | -0.577560 |
| O | | 2.013522 | | -3.262300 | | 0.248652 |
| O | | -1.281595 | | -1.263840 | | -1.313726 |
| O | | -2.336560 | | 1.406085 | | -2.030692 |
| O | | -2.567794 | | 2.469754 | | 0.273012 |
| N | | -0.900454 | | 0.584958 | | -0.024635 |
| C | | 3.206098 | | -2.607570 | | 0.147163 |
| C | | 4.382785 | | -3.318066 | | 0.377216 |
| H | | 4.321944 | | -4.380185 | | 0.588559 |
| C | | 5.605815 | | -2.651908 | | 0.330175 |
| H | | 6.519680 | | -3.215183 | | 0.501794 |
| C | | 5.689426 | | -1.274738 | | 0.063626 |
| C | | 4.498790 | | -0.576741 | | -0.138091 |
| H | | 4.522013 | | 0.495643 | | -0.319607 |
| C | | 3.252641 | | -1.221204 | | -0.103011 |
| C | | 1.994275 | | -0.508696 | | -0.221735 |
| H | | 2.022847 | | 0.574341 | | -0.217510 |
| C | | 0.836566 | | -1.192960 | | -0.337356 |
| C | | 0.919323 | | -2.699363 | | -0.495125 |
| H | | 0.019254 | | -3.190032 | | -0.123394 |
| H | | 1.026792 | | -2.961046 | | -1.558950 |
| C | | -0.509739 | | -0.630606 | | -0.598592 |
| C | | -3.688018 | | 0.122420 | | -0.124436 |
| C | | -4.131280 | | -0.823666 | | -1.048900 |
| H | | -3.665439 | | -0.878982 | | -2.023803 |
| C | | -5.154746 | | -1.689824 | | -0.677397 |
| H | | -5.506497 | | -2.431981 | | -1.388621 |
| C | | -5.735448 | | -1.627258 | | 0.598134 |
| C | | -5.268785 | | -0.663122 | | 1.502241 |
| H | | -5.712255 | | -0.597054 | | 2.491834 |
| C | | -4.251714 | | 0.220214 | | 1.148613 |
| H | | -3.910185 | | 0.984592 | | 1.837373 |
| C | | -6.862544 | | -2.557450 | | 0.973807 |
| H | | -6.929804 | | -2.691579 | | 2.057022 |
| H | | -6.737911 | | -3.542091 | | 0.513591 |
| H | | -7.824875 | | -2.157583 | | 0.631529 |
| C | | -0.202019 | | 1.277912 | | 1.067865 |
| H | | 0.341737 | | 0.519089 | | 1.635832 |
| H | | -0.963371 | | 1.696349 | | 1.730626 |
| C | | 0.742630 | | 2.386150 | | 0.635446 |
| C | | 1.688473 | | 2.851533 | | 1.557952 |
| H | | 1.733376 | | 2.408991 | | 2.550610 |
| C | | 2.578538 | | 3.867200 | | 1.212600 |
| H | | 3.308585 | | 4.215028 | | 1.937715 |
| C | | 2.534944 | | 4.429087 | | -0.065604 |
| H | | 3.229470 | | 5.217790 | | -0.339176 |
| C | | 1.596543 | | 3.967741 | | -0.988794 |
| H | | 1.556362 | | 4.396634 | | -1.985636 |
| C | | 0.702963 | | 2.951814 | | -0.642501 |
| H | | -0.010178 | | 2.591367 | | -1.376022 |
| C | | 7.029741 | | -0.582789 | | -0.013029 |
| H | | 6.925503 | | 0.502186 | | 0.078166 |
| H | | 7.528287 | | -0.785554 | | -0.968851 |
| H | | 7.702204 | | -0.924369 | | 0.780735 |

**Table S7**. Cartesian coordinates of optimized CTAB-6-Me in the excited state.

|  | X | | Y | | Z | |
| --- | --- | --- | --- | --- | --- | --- |
| S | | -1.503998 | | -0.580819 | | -1.092357 |
| O | | 3.100488 | | -1.710489 | | 2.164062 |
| O | | -0.802499 | | -0.268391 | | 2.366202 |
| O | | -0.792393 | | -1.859079 | | -1.011492 |
| O | | -1.498884 | | 0.199914 | | -2.338788 |
| N | | -0.867397 | | 0.402722 | | 0.120645 |
| C | | 3.828427 | | -1.525565 | | 1.055706 |
| C | | 5.148662 | | -2.005237 | | 1.096998 |
| H | | 5.489456 | | -2.482129 | | 2.009658 |
| C | | 5.978970 | | -1.860601 | | -0.015239 |
| H | | 6.998397 | | -2.229377 | | 0.011555 |
| C | | 5.482999 | | -1.236645 | | -1.164620 |
| C | | 4.133083 | | -0.755873 | | -1.180583 |
| H | | 3.764512 | | -0.276857 | | -2.083552 |
| C | | 3.281172 | | -0.877156 | | -0.101099 |
| C | | 1.912173 | | -0.407618 | | -0.066881 |
| H | | 1.498938 | | 0.096513 | | -0.929185 |
| C | | 1.151273 | | -0.605108 | | 1.065823 |
| C | | 1.731217 | | -1.281754 | | 2.260432 |
| H | | 1.684876 | | -0.636656 | | 3.148133 |
| H | | 1.161299 | | -2.183993 | | 2.522126 |
| C | | -0.212004 | | -0.197118 | | 1.268429 |
| C | | -3.226391 | | -0.824830 | | -0.636824 |
| C | | -3.562861 | | -1.088919 | | 0.693925 |
| H | | -2.799708 | | -1.084779 | | 1.467300 |
| C | | -4.900147 | | -1.307030 | | 1.014494 |
| H | | -5.172569 | | -1.501245 | | 2.048566 |
| C | | -5.902017 | | -1.271906 | | 0.032978 |
| C | | -5.532932 | | -0.994091 | | -1.289729 |
| H | | -6.297679 | | -0.947709 | | -2.060502 |
| C | | -4.201259 | | -0.766996 | | -1.632545 |
| H | | -3.913726 | | -0.533338 | | -2.651629 |
| C | | -7.341709 | | -1.545134 | | 0.394675 |
| H | | -8.030503 | | -1.086974 | | -0.320819 |
| H | | -7.582788 | | -1.165349 | | 1.392158 |
| H | | -7.543371 | | -2.623433 | | 0.400093 |
| C | | -1.524438 | | 1.704335 | | 0.349167 |
| H | | -2.006012 | | 1.687617 | | 1.331585 |
| H | | -2.305314 | | 1.831430 | | -0.405631 |
| C | | -0.511240 | | 2.821881 | | 0.241694 |
| C | | -0.200088 | | 3.625626 | | 1.339756 |
| H | | -0.699704 | | 3.450502 | | 2.289099 |
| C | | 0.753193 | | 4.641828 | | 1.225463 |
| H | | 0.989427 | | 5.260912 | | 2.086386 |
| C | | 1.402495 | | 4.855383 | | 0.010179 |
| H | | 2.143900 | | 5.644276 | | -0.080422 |
| C | | 1.097889 | | 4.048755 | | -1.091608 |
| H | | 1.601963 | | 4.212224 | | -2.040199 |
| C | | 0.148307 | | 3.036563 | | -0.976505 |
| H | | -0.089589 | | 2.394010 | | -1.820389 |
| C | | 6.328439 | | -1.056653 | | -2.388018 |
| H | | 6.408230 | | 0.006148 | | -2.650039 |
| H | | 5.867155 | | -1.552748 | | -3.251585 |
| H | | 7.335586 | | -1.457459 | | -2.255776 |

**Table S8**. Cartesian coordinates of optimized CTAB-6-Br in the ground state.

|  | X | | Y | | Z | |
| --- | --- | --- | --- | --- | --- | --- |
| S | | -3.206650 | | 1.279861 | | -0.553858 |
| O | | 1.307817 | | -3.191172 | | 0.228592 |
| O | | -2.016574 | | -1.234084 | | -1.325572 |
| O | | -3.135641 | | 1.420150 | | -2.005042 |
| O | | -3.390169 | | 2.448206 | | 0.312523 |
| N | | -1.679449 | | 0.604557 | | -0.009333 |
| C | | 2.486730 | | -2.517609 | | 0.132007 |
| C | | 3.673040 | | -3.216317 | | 0.352134 |
| H | | 3.627390 | | -4.281530 | | 0.549630 |
| C | | 4.892292 | | -2.541641 | | 0.311653 |
| H | | 5.819784 | | -3.079494 | | 0.470689 |
| C | | 4.916689 | | -1.167846 | | 0.058864 |
| C | | 3.739557 | | -0.457018 | | -0.141262 |
| H | | 3.763659 | | 0.614036 | | -0.312756 |
| C | | 2.508855 | | -1.127435 | | -0.108064 |
| C | | 1.238594 | | -0.432834 | | -0.224071 |
| H | | 1.250790 | | 0.650111 | | -0.217810 |
| C | | 0.093551 | | -1.135340 | | -0.338167 |
| C | | 0.193509 | | -2.640330 | | -0.496758 |
| H | | -0.692980 | | -3.142924 | | -0.108907 |
| H | | 0.285734 | | -2.901189 | | -1.561580 |
| C | | -1.264125 | | -0.592199 | | -0.598011 |
| C | | -4.454756 | | 0.079379 | | -0.115146 |
| C | | -4.878415 | | -0.861744 | | -1.053825 |
| H | | -4.415479 | | -0.889646 | | -2.031325 |
| C | | -5.879418 | | -1.758197 | | -0.693144 |
| H | | -6.216135 | | -2.496651 | | -1.415364 |
| C | | -6.456783 | | -1.730273 | | 0.585146 |
| C | | -6.009932 | | -0.770130 | | 1.503601 |
| H | | -6.451339 | | -0.730830 | | 2.495496 |
| C | | -5.015667 | | 0.142988 | | 1.161397 |
| H | | -4.689988 | | 0.903841 | | 1.861660 |
| C | | -7.560216 | | -2.692790 | | 0.949110 |
| H | | -7.619683 | | -2.847061 | | 2.030063 |
| H | | -7.414978 | | -3.666095 | | 0.471238 |
| H | | -8.532876 | | -2.309233 | | 0.617616 |
| C | | -0.985393 | | 1.309475 | | 1.079126 |
| H | | -0.435374 | | 0.558998 | | 1.652291 |
| H | | -1.751386 | | 1.724499 | | 1.738425 |
| C | | -0.050137 | | 2.422352 | | 0.638494 |
| C | | 0.917594 | | 2.876247 | | 1.544070 |
| H | | 0.984691 | | 2.423355 | | 2.530772 |
| C | | 1.803255 | | 3.892189 | | 1.188006 |
| H | | 2.551895 | | 4.229575 | | 1.898781 |
| C | | 1.732320 | | 4.466227 | | -0.083542 |
| H | | 2.424321 | | 5.253903 | | -0.365932 |
| C | | 0.771055 | | 4.017390 | | -0.989277 |
| H | | 0.710136 | | 4.455701 | | -1.980905 |
| C | | -0.117753 | | 3.000805 | | -0.632697 |
| H | | -0.847186 | | 2.649013 | | -1.354335 |
| Br | | 6.594897 | | -0.250427 | | 0.002784 |

**Table S9**. Cartesian coordinates of optimized CTAB-6-Br in the excited state.

|  | X | | Y | | Z | |
| --- | --- | --- | --- | --- | --- | --- |
| S | | -2.027054 | | -0.722787 | | -0.992008 |
| O | | 2.186031 | | -1.118673 | | 2.892236 |
| O | | -1.778155 | | 0.085922 | | 2.454950 |
| O | | -1.262713 | | -1.926440 | | -0.654608 |
| O | | -1.903637 | | -0.106623 | | -2.321220 |
| N | | -1.605939 | | 0.448543 | | 0.146474 |
| C | | 3.032320 | | -1.026233 | | 1.860138 |
| C | | 4.360203 | | -1.403389 | | 2.116234 |
| H | | 4.613874 | | -1.736657 | | 3.116548 |
| C | | 5.316772 | | -1.344922 | | 1.104387 |
| H | | 6.345354 | | -1.630598 | | 1.281166 |
| C | | 4.897284 | | -0.902326 | | -0.152931 |
| C | | 3.557276 | | -0.519598 | | -0.421944 |
| H | | 3.288246 | | -0.188051 | | -1.418066 |
| C | | 2.591177 | | -0.567822 | | 0.570940 |
| C | | 1.211043 | | -0.196000 | | 0.386744 |
| H | | 0.878740 | | 0.165519 | | -0.576264 |
| C | | 0.329061 | | -0.296242 | | 1.444526 |
| C | | 0.794587 | | -0.773466 | | 2.776857 |
| H | | 0.608494 | | -0.023631 | | 3.557767 |
| H | | 0.243947 | | -1.670556 | | 3.092335 |
| C | | -1.065759 | | 0.047781 | | 1.431785 |
| C | | -3.777153 | | -1.026098 | | -0.715839 |
| C | | -4.265526 | | -1.142155 | | 0.588689 |
| H | | -3.608801 | | -0.986733 | | 1.440034 |
| C | | -5.618944 | | -1.411251 | | 0.774165 |
| H | | -6.009967 | | -1.491262 | | 1.785024 |
| C | | -6.487906 | | -1.571739 | | -0.315589 |
| C | | -5.968827 | | -1.439053 | | -1.610222 |
| H | | -6.630468 | | -1.544985 | | -2.465789 |
| C | | -4.618687 | | -1.164155 | | -1.819486 |
| H | | -4.216766 | | -1.042395 | | -2.819193 |
| C | | -7.944983 | | -1.897597 | | -0.094925 |
| H | | -8.561418 | | -1.577677 | | -0.939847 |
| H | | -8.329896 | | -1.417813 | | 0.809986 |
| H | | -8.087557 | | -2.978700 | | 0.024541 |
| C | | -2.355997 | | 1.719950 | | 0.119503 |
| H | | -2.966058 | | 1.789057 | | 1.025123 |
| H | | -3.030616 | | 1.699838 | | -0.740588 |
| C | | -1.395321 | | 2.881606 | | 0.001455 |
| C | | -1.260996 | | 3.817368 | | 1.028579 |
| H | | -1.865373 | | 3.716253 | | 1.926344 |
| C | | -0.349878 | | 4.871087 | | 0.910606 |
| H | | -0.251707 | | 5.593660 | | 1.715867 |
| C | | 0.434253 | | 4.989977 | | -0.236218 |
| H | | 1.143194 | | 5.807820 | | -0.329321 |
| C | | 0.307099 | | 4.051247 | | -1.265775 |
| H | | 0.916818 | | 4.140644 | | -2.160615 |
| C | | -0.600766 | | 3.001865 | | -1.147146 |
| H | | -0.700876 | | 2.257755 | | -1.933273 |
| Br | | 6.149745 | | -0.803520 | | -1.557801 |

**Table S10**. Cartesian coordinates of optimized CTAB-6-Cl in the ground state.

|  | X | | Y | | Z | |
| --- | --- | --- | --- | --- | --- | --- |
| S | | -2.680965 | | 1.292555 | | -0.553931 |
| O | | 1.787596 | | -3.221581 | | 0.234351 |
| O | | -1.514365 | | -1.232326 | | -1.326080 |
| O | | -2.608082 | | 1.432469 | | -2.005056 |
| O | | -2.853542 | | 2.462345 | | 0.312724 |
| N | | -1.160452 | | 0.602464 | | -0.008975 |
| C | | 2.973968 | | -2.561019 | | 0.135305 |
| C | | 4.152786 | | -3.272003 | | 0.355433 |
| H | | 4.095638 | | -4.336243 | | 0.554860 |
| C | | 5.379367 | | -2.611109 | | 0.312426 |
| H | | 6.302513 | | -3.156868 | | 0.470978 |
| C | | 5.418739 | | -1.237623 | | 0.056808 |
| C | | 4.248633 | | -0.514135 | | -0.143133 |
| H | | 4.285621 | | 0.556334 | | -0.316705 |
| C | | 3.011142 | | -1.171642 | | -0.107074 |
| C | | 1.747927 | | -0.463868 | | -0.223053 |
| H | | 1.770827 | | 0.618961 | | -0.217218 |
| C | | 0.595700 | | -1.154674 | | -0.336781 |
| C | | 0.680516 | | -2.660734 | | -0.494017 |
| H | | -0.211832 | | -3.153894 | | -0.107519 |
| H | | 0.772282 | | -2.923403 | | -1.558475 |
| C | | -0.756354 | | -0.598005 | | -0.597671 |
| C | | -3.940480 | | 0.103806 | | -0.115925 |
| C | | -4.372704 | | -0.833028 | | -1.054988 |
| H | | -3.909789 | | -0.864980 | | -2.032373 |
| C | | -5.382167 | | -1.720156 | | -0.694822 |
| H | | -5.725605 | | -2.455217 | | -1.417337 |
| C | | -5.959573 | | -1.687178 | | 0.583328 |
| C | | -5.503979 | | -0.731520 | | 1.502172 |
| H | | -5.945262 | | -0.688366 | | 2.493961 |
| C | | -4.501098 | | 0.172320 | | 1.160495 |
| H | | -4.168461 | | 0.929873 | | 1.861065 |
| C | | -7.072055 | | -2.639428 | | 0.946750 |
| H | | -7.133128 | | -2.793561 | | 2.027633 |
| H | | -6.935897 | | -3.613864 | | 0.468512 |
| H | | -8.041017 | | -2.246615 | | 0.615269 |
| C | | -0.459852 | | 1.300811 | | 1.079429 |
| H | | 0.083857 | | 0.545314 | | 1.652018 |
| H | | -1.221849 | | 1.722228 | | 1.739316 |
| C | | 0.484746 | | 2.405780 | | 0.638838 |
| C | | 1.453624 | | 2.854190 | | 1.545918 |
| H | | 1.515017 | | 2.402606 | | 2.533595 |
| C | | 2.347196 | | 3.863337 | | 1.190339 |
| H | | 3.096407 | | 4.196737 | | 1.902398 |
| C | | 2.283268 | | 4.435870 | | -0.082272 |
| H | | 2.981332 | | 5.218345 | | -0.364200 |
| C | | 1.321026 | | 3.992341 | | -0.989563 |
| H | | 1.265409 | | 4.429576 | | -1.981978 |
| C | | 0.424220 | | 2.982626 | | -0.633434 |
| H | | -0.306314 | | 2.635138 | | -1.356051 |
| Cl | | 6.971083 | | -0.410426 | | 0.001377 |

**Table S11**. Cartesian coordinates of optimized CTAB-6-Cl in the excited state.

|  | X | | Y | | Z | |
| --- | --- | --- | --- | --- | --- | --- |
| S | | -1.650329 | | -0.653611 | | -1.049623 |
| O | | 2.807256 | | -1.448340 | | 2.474579 |
| O | | -1.120734 | | -0.069696 | | 2.415312 |
| O | | -0.907233 | | -1.899561 | | -0.844078 |
| O | | -1.599574 | | 0.043254 | | -2.343004 |
| N | | -1.107532 | | 0.428802 | | 0.126582 |
| C | | 3.580443 | | -1.320201 | | 1.389274 |
| C | | 4.906305 | | -1.761557 | | 1.521586 |
| H | | 5.217621 | | -2.167786 | | 2.477615 |
| C | | 5.788672 | | -1.672796 | | 0.446295 |
| H | | 6.815252 | | -2.006111 | | 0.525151 |
| C | | 5.298332 | | -1.134542 | | -0.747259 |
| C | | 3.958969 | | -0.686624 | | -0.890511 |
| H | | 3.633922 | | -0.282127 | | -1.842143 |
| C | | 3.066251 | | -0.763226 | | 0.167280 |
| C | | 1.693279 | | -0.329644 | | 0.110812 |
| H | | 1.306992 | | 0.106586 | | -0.799675 |
| C | | 0.886966 | | -0.466667 | | 1.223967 |
| C | | 1.427976 | | -1.046810 | | 2.483801 |
| H | | 1.327926 | | -0.344812 | | 3.323237 |
| H | | 0.865385 | | -1.942444 | | 2.783248 |
| C | | -0.490239 | | -0.070842 | | 1.339271 |
| C | | -3.385530 | | -0.918744 | | -0.663802 |
| C | | -3.781745 | | -1.107354 | | 0.663351 |
| H | | -3.059761 | | -1.031165 | | 1.471463 |
| C | | -5.127090 | | -1.344659 | | 0.931860 |
| H | | -5.446334 | | -1.480442 | | 1.961784 |
| C | | -6.078313 | | -1.402695 | | -0.097882 |
| C | | -5.650146 | | -1.199124 | | -1.416347 |
| H | | -6.375673 | | -1.225386 | | -2.224970 |
| C | | -4.309527 | | -0.954202 | | -1.707930 |
| H | | -3.977111 | | -0.777932 | | -2.724888 |
| C | | -7.526541 | | -1.695712 | | 0.209732 |
| H | | -8.190481 | | -1.299776 | | -0.563970 |
| H | | -7.826603 | | -1.266132 | | 1.170262 |
| H | | -7.699887 | | -2.777291 | | 0.268932 |
| C | | -1.816392 | | 1.719563 | | 0.236707 |
| H | | -2.362509 | | 1.743296 | | 1.184429 |
| H | | -2.546479 | | 1.777357 | | -0.574954 |
| C | | -0.828147 | | 2.858910 | | 0.130978 |
| C | | -0.592075 | | 3.714765 | | 1.208251 |
| H | | -1.135746 | | 3.566700 | | 2.137736 |
| C | | 0.342964 | | 4.748190 | | 1.098537 |
| H | | 0.520481 | | 5.408591 | | 1.942651 |
| C | | 1.049337 | | 4.926324 | | -0.090420 |
| H | | 1.776739 | | 5.728483 | | -0.177188 |
| C | | 0.820385 | | 4.067272 | | -1.170660 |
| H | | 1.369419 | | 4.202929 | | -2.098345 |
| C | | -0.111395 | | 3.038132 | | -1.060298 |
| H | | -0.290258 | | 2.355166 | | -1.886925 |
| Cl | | 6.356527 | | -1.001373 | | -2.115740 |

**Table S12**. Cartesian coordinates of optimized CTAB-7-OMe in the ground state.

|  | X | | Y | | Z | |
| --- | --- | --- | --- | --- | --- | --- |
| S | | -2.745048 | | 0.710974 | | -1.201965 |
| O | | 2.571336 | | -2.656308 | | -0.046863 |
| O | | -1.343510 | | -1.862394 | | -0.873223 |
| O | | -2.575050 | | 0.199927 | | -2.556260 |
| O | | -3.000510 | | 2.134413 | | -0.946299 |
| N | | -1.269012 | | 0.393596 | | -0.351924 |
| C | | 3.472058 | | -1.634977 | | -0.030280 |
| C | | 4.821996 | | -1.968913 | | 0.078369 |
| H | | 5.084343 | | -3.012380 | | 0.190214 |
| C | | 5.777791 | | -0.946577 | | 0.045300 |
| C | | 5.384810 | | 0.397608 | | -0.103759 |
| C | | 4.042876 | | 0.706084 | | -0.237989 |
| H | | 3.728485 | | 1.738452 | | -0.363440 |
| C | | 3.057888 | | -0.297705 | | -0.202437 |
| C | | 1.652268 | | -0.045571 | | -0.420448 |
| H | | 1.354405 | | 0.908660 | | -0.837130 |
| C | | 0.742229 | | -1.011803 | | -0.169150 |
| C | | 1.231356 | | -2.336455 | | 0.371172 |
| H | | 1.205177 | | -2.333903 | | 1.473633 |
| H | | 0.601367 | | -3.150065 | | 0.010403 |
| C | | -0.690916 | | -0.899092 | | -0.497166 |
| C | | -4.028337 | | -0.208419 | | -0.358708 |
| C | | -4.285428 | | -1.533529 | | -0.716523 |
| H | | -3.695542 | | -2.004261 | | -1.491705 |
| C | | -5.290383 | | -2.223211 | | -0.045575 |
| H | | -5.497336 | | -3.255015 | | -0.316169 |
| C | | -6.039494 | | -1.614293 | | 0.972766 |
| C | | -5.761590 | | -0.281606 | | 1.304000 |
| H | | -6.338390 | | 0.208669 | | 2.083373 |
| C | | -4.764232 | | 0.430810 | | 0.641058 |
| H | | -4.571046 | | 1.471767 | | 0.873953 |
| C | | -7.143813 | | -2.371696 | | 1.668389 |
| H | | -8.064311 | | -2.348977 | | 1.072437 |
| H | | -7.374756 | | -1.940037 | | 2.646182 |
| H | | -6.877075 | | -3.423064 | | 1.812357 |
| C | | -1.011732 | | 1.197082 | | 0.875366 |
| H | | -0.693850 | | 0.508130 | | 1.661989 |
| H | | -1.970856 | | 1.617927 | | 1.188209 |
| C | | 0.006396 | | 2.308923 | | 0.726795 |
| C | | -0.067320 | | 3.229300 | | -0.326442 |
| H | | -0.881560 | | 3.158038 | | -1.038620 |
| C | | 0.904973 | | 4.220445 | | -0.459145 |
| H | | 0.843441 | | 4.924147 | | -1.284125 |
| C | | 1.953225 | | 4.312621 | | 0.461005 |
| H | | 2.709620 | | 5.084287 | | 0.351647 |
| C | | 2.021703 | | 3.408332 | | 1.521636 |
| H | | 2.833966 | | 3.467288 | | 2.240024 |
| C | | 1.052932 | | 2.411367 | | 1.649438 |
| H | | 1.127830 | | 1.689888 | | 2.459077 |
| H | | 6.152859 | | 1.162187 | | -0.120960 |
| O | | 7.116570 | | -1.152516 | | 0.158980 |
| C | | 7.590230 | | -2.487107 | | 0.289217 |
| H | | 7.316134 | | -3.098684 | | -0.579603 |
| H | | 8.676741 | | -2.414545 | | 0.350994 |
| H | | 7.206590 | | -2.963942 | | 1.200179 |

**Table S13**. Cartesian coordinates of optimized CTAB-7-OMe in the excited state.

|  | X | | Y | | Z | |
| --- | --- | --- | --- | --- | --- | --- |
| S | | -2.669695 | | 0.481307 | | -1.311740 |
| O | | 2.724546 | | -2.591606 | | 0.121940 |
| O | | -1.365342 | | -2.101116 | | -0.383197 |
| O | | -2.483102 | | -0.279693 | | -2.543649 |
| O | | -2.876422 | | 1.937872 | | -1.331869 |
| N | | -1.259153 | | 0.252215 | | -0.377180 |
| C | | 3.557501 | | -1.544834 | | -0.024054 |
| C | | 4.914641 | | -1.805010 | | 0.201793 |
| H | | 5.201383 | | -2.815079 | | 0.463534 |
| C | | 5.830222 | | -0.759450 | | 0.086292 |
| C | | 5.365694 | | 0.533402 | | -0.252574 |
| C | | 4.007175 | | 0.777704 | | -0.474929 |
| H | | 3.670136 | | 1.781178 | | -0.713593 |
| C | | 3.057095 | | -0.246320 | | -0.389859 |
| C | | 1.649654 | | -0.067080 | | -0.611275 |
| H | | 1.285982 | | 0.893601 | | -0.941468 |
| C | | 0.773779 | | -1.109931 | | -0.336764 |
| C | | 1.309260 | | -2.467782 | | -0.054167 |
| H | | 0.856958 | | -2.909605 | | 0.845000 |
| H | | 1.047760 | | -3.161944 | | -0.869335 |
| C | | -0.670514 | | -1.070215 | | -0.363341 |
| C | | -4.047422 | | -0.205163 | | -0.389094 |
| C | | -4.300935 | | -1.578268 | | -0.435298 |
| H | | -3.649507 | | -2.223538 | | -1.009613 |
| C | | -5.375601 | | -2.084732 | | 0.288023 |
| H | | -5.579449 | | -3.151903 | | 0.258829 |
| C | | -6.199438 | | -1.247429 | | 1.056563 |
| C | | -5.922109 | | 0.124687 | | 1.080751 |
| H | | -6.553075 | | 0.789434 | | 1.664596 |
| C | | -4.853753 | | 0.654751 | | 0.357998 |
| H | | -4.654740 | | 1.720333 | | 0.351508 |
| C | | -7.375389 | | -1.816536 | | 1.812415 |
| H | | -8.222193 | | -1.994707 | | 1.138390 |
| H | | -7.717231 | | -1.136839 | | 2.597908 |
| H | | -7.124536 | | -2.775837 | | 2.275992 |
| C | | -1.120872 | | 1.101464 | | 0.837578 |
| H | | -0.917019 | | 0.442425 | | 1.686606 |
| H | | -2.091778 | | 1.569759 | | 1.025822 |
| C | | -0.054630 | | 2.173945 | | 0.740692 |
| C | | -0.076570 | | 3.112625 | | -0.299749 |
| H | | -0.878411 | | 3.072160 | | -1.029161 |
| C | | 0.937338 | | 4.065199 | | -0.404661 |
| H | | 0.915979 | | 4.783620 | | -1.219248 |
| C | | 1.975771 | | 4.100907 | | 0.530668 |
| H | | 2.762914 | | 4.844775 | | 0.445220 |
| C | | 1.992363 | | 3.178510 | | 1.578879 |
| H | | 2.795740 | | 3.195389 | | 2.309749 |
| C | | 0.981206 | | 2.221431 | | 1.679266 |
| H | | 1.011415 | | 1.486885 | | 2.480181 |
| H | | 6.096436 | | 1.330631 | | -0.335679 |
| O | | 7.172050 | | -0.866033 | | 0.278510 |
| C | | 7.700728 | | -2.142870 | | 0.612946 |
| H | | 7.499676 | | -2.879395 | | -0.175994 |
| H | | 8.777719 | | -2.005922 | | 0.714130 |
| H | | 7.290021 | | -2.511251 | | 1.562246 |

**Table S14**. Cartesian coordinates of optimized CTAB-7-Me in the ground state.

|  | X | | Y | | Z | |
| --- | --- | --- | --- | --- | --- | --- |
| S | | -2.531579 | | 1.162789 | | -0.558864 |
| O | | 2.321321 | | -2.948649 | | 0.149622 |
| O | | -1.175358 | | -1.260427 | | -1.333040 |
| O | | -2.489100 | | 1.301808 | | -2.011536 |
| O | | -2.792191 | | 2.318899 | | 0.304844 |
| N | | -0.956412 | | 0.605088 | | -0.030512 |
| C | | 3.443416 | | -2.180583 | | 0.053280 |
| C | | 4.679554 | | -2.785895 | | 0.263181 |
| H | | 4.708007 | | -3.854429 | | 0.452123 |
| C | | 5.855176 | | -2.026824 | | 0.224955 |
| C | | 5.767715 | | -0.644900 | | -0.012853 |
| C | | 4.531676 | | -0.037239 | | -0.200115 |
| H | | 4.464923 | | 1.036095 | | -0.358725 |
| C | | 3.348893 | | -0.790628 | | -0.173352 |
| C | | 2.027994 | | -0.201686 | | -0.269247 |
| H | | 1.952124 | | 0.878972 | | -0.247734 |
| C | | 0.939858 | | -0.992890 | | -0.384999 |
| C | | 1.167218 | | -2.480786 | | -0.570754 |
| H | | 0.324475 | | -3.063670 | | -0.197940 |
| H | | 1.286526 | | -2.713346 | | -1.639928 |
| C | | -0.457486 | | -0.561983 | | -0.622060 |
| C | | -3.688752 | | -0.120687 | | -0.102386 |
| C | | -4.050507 | | -1.097703 | | -1.030263 |
| H | | -3.592483 | | -1.101177 | | -2.010418 |
| C | | -4.982925 | | -2.059893 | | -0.655614 |
| H | | -5.270592 | | -2.826718 | | -1.369497 |
| C | | -5.552803 | | -2.062399 | | 0.626374 |
| C | | -5.169823 | | -1.065140 | | 1.533734 |
| H | | -5.606609 | | -1.048821 | | 2.528355 |
| C | | -4.245157 | | -0.086476 | | 1.177098 |
| H | | -3.970377 | | 0.701864 | | 1.868702 |
| C | | -6.582136 | | -3.098562 | | 1.005315 |
| H | | -6.633064 | | -3.238258 | | 2.088690 |
| H | | -6.362503 | | -4.066415 | | 0.544905 |
| H | | -7.579959 | | -2.795500 | | 0.665295 |
| C | | -0.316373 | | 1.349298 | | 1.063865 |
| H | | 0.306274 | | 0.640378 | | 1.615083 |
| H | | -1.106815 | | 1.682045 | | 1.740988 |
| C | | 0.509034 | | 2.551028 | | 0.637257 |
| C | | 1.410613 | | 3.099389 | | 1.558481 |
| H | | 1.507909 | | 2.652872 | | 2.545604 |
| C | | 2.190464 | | 4.203761 | | 1.219013 |
| H | | 2.887527 | | 4.615631 | | 1.943031 |
| C | | 2.079671 | | 4.772590 | | -0.052042 |
| H | | 2.688523 | | 5.630605 | | -0.321042 |
| C | | 1.185317 | | 4.228821 | | -0.974066 |
| H | | 1.093714 | | 4.662568 | | -1.965396 |
| C | | 0.401980 | | 3.123842 | | -0.633595 |
| H | | -0.277448 | | 2.701421 | | -1.366039 |
| H | | 6.674520 | | -0.047826 | | -0.039983 |
| C | | 7.196980 | | -2.694957 | | 0.399960 |
| H | | 7.131194 | | -3.558161 | | 1.068845 |
| H | | 7.580281 | | -3.056609 | | -0.562328 |
| H | | 7.939040 | | -2.001807 | | 0.806691 |

**Table S15**. Cartesian coordinates of optimized CTAB-7-Me in the excited state.

|  | X | | Y | | Z | |
| --- | --- | --- | --- | --- | --- | --- |
| S | | -1.562806 | | -0.840862 | | -1.145693 |
| O | | 3.250411 | | -1.041008 | | 2.002638 |
| O | | -0.690575 | | 0.272413 | | 2.108777 |
| O | | -0.756910 | | -2.029845 | | -0.856287 |
| O | | -1.700943 | | -0.344671 | | -2.521129 |
| N | | -0.903017 | | 0.425006 | | -0.221100 |
| C | | 3.916435 | | -1.095938 | | 0.838316 |
| C | | 5.257177 | | -1.497227 | | 0.919278 |
| H | | 5.658814 | | -1.726457 | | 1.901525 |
| C | | 6.045125 | | -1.595157 | | -0.235987 |
| C | | 5.426984 | | -1.275415 | | -1.452892 |
| C | | 4.072903 | | -0.870031 | | -1.532984 |
| H | | 3.643990 | | -0.638203 | | -2.502713 |
| C | | 3.272968 | | -0.762814 | | -0.403428 |
| C | | 1.891977 | | -0.355008 | | -0.396834 |
| H | | 1.408742 | | -0.085545 | | -1.325722 |
| C | | 1.197606 | | -0.303520 | | 0.797716 |
| C | | 1.871566 | | -0.659547 | | 2.074220 |
| H | | 1.828202 | | 0.170879 | | 2.794165 |
| H | | 1.362550 | | -1.496797 | | 2.574931 |
| C | | -0.168739 | | 0.103759 | | 0.986174 |
| C | | -3.224053 | | -1.075300 | | -0.500033 |
| C | | -3.434529 | | -1.133653 | | 0.880961 |
| H | | -2.612399 | | -0.975411 | | 1.572379 |
| C | | -4.727502 | | -1.343183 | | 1.352386 |
| H | | -4.901894 | | -1.377110 | | 2.424573 |
| C | | -5.809064 | | -1.502221 | | 0.472769 |
| C | | -5.566550 | | -1.428509 | | -0.905039 |
| H | | -6.394473 | | -1.535339 | | -1.600848 |
| C | | -4.281442 | | -1.211894 | | -1.399154 |
| H | | -4.092149 | | -1.137167 | | -2.464182 |
| C | | -7.197673 | | -1.763862 | | 1.002975 |
| H | | -7.966043 | | -1.463963 | | 0.284784 |
| H | | -7.376353 | | -1.226116 | | 1.939081 |
| H | | -7.339997 | | -2.831768 | | 1.209641 |
| C | | -1.708216 | | 1.665808 | | -0.154726 |
| H | | -2.362973 | | 1.638241 | | 0.721960 |
| H | | -2.328386 | | 1.699630 | | -1.053379 |
| C | | -0.781485 | | 2.857315 | | -0.109722 |
| C | | -0.462820 | | 3.465868 | | 1.108021 |
| H | | -0.898634 | | 3.075154 | | 2.022834 |
| C | | 0.427064 | | 4.541528 | | 1.146652 |
| H | | 0.667855 | | 5.009844 | | 2.096909 |
| C | | 1.007644 | | 5.011729 | | -0.031967 |
| H | | 1.697741 | | 5.850259 | | -0.002861 |
| C | | 0.701397 | | 4.398983 | | -1.250520 |
| H | | 1.154241 | | 4.759417 | | -2.169895 |
| C | | -0.185865 | | 3.324796 | | -1.287343 |
| H | | -0.417792 | | 2.832615 | | -2.228419 |
| H | | 6.003876 | | -1.339752 | | -2.371366 |
| C | | 7.487176 | | -2.024051 | | -0.156151 |
| H | | 8.077326 | | -1.330232 | | 0.454469 |
| H | | 7.584205 | | -3.016017 | | 0.300544 |
| H | | 7.945190 | | -2.067274 | | -1.147735 |

**Table S16**. Cartesian coordinates of optimized CTAB-7-Br in the ground state.

|  | X | | Y | | Z | |
| --- | --- | --- | --- | --- | --- | --- |
| S | | -3.520761 | | 0.921171 | | -0.503297 |
| O | | 1.947827 | | -2.367866 | | 0.028304 |
| O | | -1.799605 | | -1.228900 | | -1.368057 |
| O | | -3.529020 | | 1.102397 | | -1.951728 |
| O | | -3.945872 | | 1.996701 | | 0.397812 |
| N | | -1.862254 | | 0.613664 | | -0.016176 |
| C | | 2.927087 | | -1.430644 | | -0.078508 |
| C | | 4.248625 | | -1.841794 | | 0.088122 |
| H | | 4.468316 | | -2.889060 | | 0.252770 |
| C | | 5.255817 | | -0.881048 | | 0.031986 |
| C | | 4.980746 | | 0.472718 | | -0.177795 |
| C | | 3.654072 | | 0.865817 | | -0.321772 |
| H | | 3.412608 | | 1.915909 | | -0.462616 |
| C | | 2.610397 | | -0.069071 | | -0.276938 |
| C | | 1.209507 | | 0.303577 | | -0.336413 |
| H | | 0.963405 | | 1.357873 | | -0.297475 |
| C | | 0.259552 | | -0.648667 | | -0.440252 |
| C | | 0.709215 | | -2.081665 | | -0.648712 |
| H | | -0.017380 | | -2.791122 | | -0.251871 |
| H | | 0.825735 | | -2.288860 | | -1.722748 |
| C | | -1.195166 | | -0.440265 | | -0.646512 |
| C | | -4.440208 | | -0.546637 | | -0.065185 |
| C | | -4.659055 | | -1.543070 | | -1.016863 |
| H | | -4.230929 | | -1.443164 | | -2.005443 |
| C | | -5.411610 | | -2.655872 | | -0.655137 |
| H | | -5.587004 | | -3.438988 | | -1.387450 |
| C | | -5.942060 | | -2.788329 | | 0.636923 |
| C | | -5.705885 | | -1.767642 | | 1.568218 |
| H | | -6.115244 | | -1.851663 | | 2.571013 |
| C | | -4.963200 | | -0.640426 | | 1.225455 |
| H | | -4.804558 | | 0.162320 | | 1.936620 |
| C | | -6.778029 | | -3.990003 | | 1.002452 |
| H | | -6.777595 | | -4.167835 | | 2.081389 |
| H | | -6.415261 | | -4.894697 | | 0.505665 |
| H | | -7.820285 | | -3.844637 | | 0.693223 |
| C | | -1.322741 | | 1.435685 | | 1.077077 |
| H | | -0.587258 | | 0.826182 | | 1.607941 |
| H | | -2.142361 | | 1.633582 | | 1.771992 |
| C | | -0.702152 | | 2.755363 | | 0.651858 |
| C | | 0.134242 | | 3.419755 | | 1.558210 |
| H | | 0.327594 | | 2.978626 | | 2.533531 |
| C | | 0.728450 | | 4.634079 | | 1.218325 |
| H | | 1.377138 | | 5.135790 | | 1.930462 |
| C | | 0.494715 | | 5.198336 | | -0.038064 |
| H | | 0.959129 | | 6.142241 | | -0.307370 |
| C | | -0.335576 | | 4.539782 | | -0.945073 |
| H | | -0.521619 | | 4.969515 | | -1.924820 |
| C | | -0.933125 | | 3.324262 | | -0.604313 |
| H | | -1.563103 | | 2.815379 | | -1.325959 |
| H | | 5.786775 | | 1.194949 | | -0.217057 |
| Br | | 7.071471 | | -1.437738 | | 0.242600 |

**Table S17**. Cartesian coordinates of optimized CTAB-7-Br in the excited state.

|  | X | | Y | | Z | |
| --- | --- | --- | --- | --- | --- | --- |
| S | | -3.090698 | | 0.224917 | | -1.357947 |
| O | | 2.279756 | | -2.425465 | | 0.503081 |
| O | | -1.843558 | | -2.093007 | | 0.309578 |
| O | | -2.730161 | | -0.757633 | | -2.377540 |
| O | | -3.284796 | | 1.645196 | | -1.687514 |
| N | | -1.827987 | | 0.203640 | | -0.217799 |
| C | | 3.065919 | | -1.397520 | | 0.137241 |
| C | | 4.443897 | | -1.606501 | | 0.252338 |
| H | | 4.806180 | | -2.563092 | | 0.608341 |
| C | | 5.308796 | | -0.570121 | | -0.093601 |
| C | | 4.794324 | | 0.655848 | | -0.545831 |
| C | | 3.409382 | | 0.850270 | | -0.658898 |
| H | | 3.026958 | | 1.809587 | | -0.991779 |
| C | | 2.496972 | | -0.161313 | | -0.338635 |
| C | | 1.077510 | | -0.033769 | | -0.436426 |
| H | | 0.655043 | | 0.876659 | | -0.833848 |
| C | | 0.254479 | | -1.060548 | | 0.012442 |
| C | | 0.853440 | | -2.340897 | | 0.467969 |
| H | | 0.518403 | | -2.615562 | | 1.479792 |
| H | | 0.520164 | | -3.178401 | | -0.166603 |
| C | | -1.186058 | | -1.069677 | | 0.055584 |
| C | | -4.585980 | | -0.308772 | | -0.522281 |
| C | | -4.791988 | | -1.667824 | | -0.271539 |
| H | | -4.038349 | | -2.388359 | | -0.561886 |
| C | | -5.956985 | | -2.058550 | | 0.379912 |
| H | | -6.124432 | | -3.113312 | | 0.581305 |
| C | | -6.918385 | | -1.119223 | | 0.784915 |
| C | | -6.683819 | | 0.235300 | | 0.519080 |
| H | | -7.418508 | | 0.976469 | | 0.821913 |
| C | | -5.524469 | | 0.648954 | | -0.136175 |
| H | | -5.350230 | | 1.694373 | | -0.363949 |
| C | | -8.188607 | | -1.569060 | | 1.464573 |
| H | | -8.660071 | | -0.752919 | | 2.019089 |
| H | | -7.998362 | | -2.391005 | | 2.161720 |
| H | | -8.916446 | | -1.931000 | | 0.728097 |
| C | | -1.891726 | | 1.206217 | | 0.876004 |
| H | | -1.872323 | | 0.676145 | | 1.833567 |
| H | | -2.860872 | | 1.709125 | | 0.808924 |
| C | | -0.779873 | | 2.231738 | | 0.805740 |
| C | | 0.203806 | | 2.288428 | | 1.796787 |
| H | | 0.149793 | | 1.609086 | | 2.643807 |
| C | | 1.267653 | | 3.187101 | | 1.692987 |
| H | | 2.030694 | | 3.214396 | | 2.465484 |
| C | | 1.353667 | | 4.039310 | | 0.591282 |
| H | | 2.181317 | | 4.737750 | | 0.505538 |
| C | | 0.366260 | | 3.994790 | | -0.398671 |
| H | | 0.425629 | | 4.660375 | | -1.255154 |
| C | | -0.697180 | | 3.099266 | | -0.292309 |
| H | | -1.454532 | | 3.039307 | | -1.067835 |
| H | | 5.476526 | | 1.456553 | | -0.808241 |
| Br | | 7.189553 | | -0.809821 | | 0.051648 |

**Table S18**. Cartesian coordinates of optimized CTAB-7-Cl in the ground state.

|  | X | | Y | | Z | |
| --- | --- | --- | --- | --- | --- | --- |
| S | | -2.877495 | | 1.068977 | | -0.532316 |
| O | | 2.263092 | | -2.700103 | | 0.094223 |
| O | | -1.351743 | | -1.241288 | | -1.350266 |
| O | | -2.852424 | | 1.229267 | | -1.982975 |
| O | | -3.210845 | | 2.192246 | | 0.348930 |
| N | | -1.260333 | | 0.616052 | | -0.020400 |
| C | | 3.323952 | | -1.855727 | | -0.006429 |
| C | | 4.600419 | | -2.383474 | | 0.179775 |
| H | | 4.724028 | | -3.444924 | | 0.354207 |
| C | | 5.691687 | | -1.517920 | | 0.130456 |
| C | | 5.541955 | | -0.145949 | | -0.092156 |
| C | | 4.257991 | | 0.363627 | | -0.255368 |
| H | | 4.113834 | | 1.429985 | | -0.406213 |
| C | | 3.133631 | | -0.472768 | | -0.218096 |
| C | | 1.772914 | | 0.024105 | | -0.296840 |
| H | | 1.622602 | | 1.096552 | | -0.267893 |
| C | | 0.742004 | | -0.839275 | | -0.405754 |
| C | | 1.062988 | | -2.308435 | | -0.599439 |
| H | | 0.270965 | | -2.946622 | | -0.206876 |
| H | | 1.172873 | | -2.533363 | | -1.670590 |
| C | | -0.685479 | | -0.502208 | | -0.630669 |
| C | | -3.934473 | | -0.300473 | | -0.085987 |
| C | | -4.232124 | | -1.287922 | | -1.025631 |
| H | | -3.784007 | | -1.244151 | | -2.009415 |
| C | | -5.088947 | | -2.320536 | | -0.658068 |
| H | | -5.326393 | | -3.095999 | | -1.380977 |
| C | | -5.646294 | | -2.382339 | | 0.627963 |
| C | | -5.329089 | | -1.372601 | | 1.547020 |
| H | | -5.757700 | | -1.401964 | | 2.544849 |
| C | | -4.480952 | | -0.324462 | | 1.198133 |
| H | | -4.258329 | | 0.471861 | | 1.899288 |
| C | | -6.594460 | | -3.495667 | | 0.999394 |
| H | | -6.625039 | | -3.654680 | | 2.080833 |
| H | | -6.310047 | | -4.438116 | | 0.522139 |
| H | | -7.614578 | | -3.260185 | | 0.672346 |
| C | | -0.661899 | | 1.396178 | | 1.072875 |
| H | | 0.008253 | | 0.727417 | | 1.618677 |
| H | | -1.468610 | | 1.674562 | | 1.755178 |
| C | | 0.081657 | | 2.649786 | | 0.645047 |
| C | | 0.963097 | | 3.244182 | | 1.557298 |
| H | | 1.102677 | | 2.796812 | | 2.538936 |
| C | | 1.669721 | | 4.396162 | | 1.215497 |
| H | | 2.351946 | | 4.843740 | | 1.932362 |
| C | | 1.504755 | | 4.967170 | | -0.048725 |
| H | | 2.056605 | | 5.862330 | | -0.319492 |
| C | | 0.629872 | | 4.378080 | | -0.961583 |
| H | | 0.496447 | | 4.813515 | | -1.947374 |
| C | | -0.080223 | | 3.225260 | | -0.618912 |
| H | | -0.744487 | | 2.768873 | | -1.344905 |
| H | | 6.411712 | | 0.498750 | | -0.125373 |
| Cl | | 7.304626 | | -2.178243 | | 0.348356 |

**Table S19**. Cartesian coordinates of optimized CTAB-7-Cl in the excited state.

|  | X | | Y | | Z | |
| --- | --- | --- | --- | --- | --- | --- |
| S | | -2.510433 | | 0.238557 | | -1.360535 |
| O | | 2.747638 | | -2.583454 | | 0.539276 |
| O | | -1.360885 | | -2.099633 | | 0.361286 |
| O | | -2.175145 | | -0.771826 | | -2.361517 |
| O | | -2.647304 | | 1.660087 | | -1.712699 |
| N | | -1.263286 | | 0.185880 | | -0.205295 |
| C | | 3.569962 | | -1.589369 | | 0.160353 |
| C | | 4.939615 | | -1.850458 | | 0.272216 |
| H | | 5.267875 | | -2.816662 | | 0.635484 |
| C | | 5.842006 | | -0.850312 | | -0.086528 |
| C | | 5.372584 | | 0.390200 | | -0.547830 |
| C | | 3.994965 | | 0.635597 | | -0.657048 |
| H | | 3.647952 | | 1.605645 | | -0.997296 |
| C | | 3.046174 | | -0.337260 | | -0.324534 |
| C | | 1.631600 | | -0.157593 | | -0.418744 |
| H | | 1.242013 | | 0.764444 | | -0.822924 |
| C | | 0.772548 | | -1.148921 | | 0.041923 |
| C | | 1.325264 | | -2.445017 | | 0.511106 |
| H | | 0.986825 | | -2.693131 | | 1.528720 |
| H | | 0.956583 | | -3.278016 | | -0.109579 |
| C | | -0.667076 | | -1.105341 | | 0.088287 |
| C | | -4.034048 | | -0.226859 | | -0.535199 |
| C | | -4.288882 | | -1.572847 | | -0.259523 |
| H | | -3.556129 | | -2.324053 | | -0.524632 |
| C | | -5.474402 | | -1.910730 | | 0.384183 |
| H | | -5.679801 | | -2.954865 | | 0.604971 |
| C | | -6.408688 | | -0.931467 | | 0.757148 |
| C | | -6.125304 | | 0.408520 | | 0.466871 |
| H | | -6.838301 | | 1.180001 | | 0.744797 |
| C | | -4.944320 | | 0.769512 | | -0.181079 |
| H | | -4.731647 | | 1.803616 | | -0.427127 |
| C | | -7.700899 | | -1.325033 | | 1.430230 |
| H | | -8.163036 | | -0.477172 | | 1.943361 |
| H | | -7.541762 | | -2.122510 | | 2.162826 |
| H | | -8.423662 | | -1.700252 | | 0.695462 |
| C | | -1.304073 | | 1.201757 | | 0.876818 |
| H | | -1.319180 | | 0.682130 | | 1.840218 |
| H | | -2.251566 | | 1.741481 | | 0.789806 |
| C | | -0.151408 | | 2.181293 | | 0.810199 |
| C | | 0.827120 | | 2.203187 | | 1.807631 |
| H | | 0.739325 | | 1.531726 | | 2.658095 |
| C | | 1.927723 | | 3.056773 | | 1.706105 |
| H | | 2.686037 | | 3.057396 | | 2.483727 |
| C | | 2.055997 | | 3.898292 | | 0.600341 |
| H | | 2.912157 | | 4.561719 | | 0.516493 |
| C | | 1.074219 | | 3.888662 | | -0.396202 |
| H | | 1.166581 | | 4.546327 | | -1.255880 |
| C | | -0.025719 | | 3.038138 | | -0.292191 |
| H | | -0.779422 | | 3.004240 | | -1.072898 |
| H | | 6.085190 | | 1.160967 | | -0.819620 |
| Cl | | 7.562336 | | -1.138637 | | 0.043157 |

**7. References**

1. Chen D, Zhang Y and Pan X *et al*. Oxidation of Tertiary Aromatic Alcohols to Ketones in Water. *Adv Synth Catal* 2018; **360**: 3607-3612.
2. Kurtz KCM, Hsung RP and Zhang Y. A Ring-Closing Yne-Carbonyl Metathesis of Ynamides. *Org Lett* 2006; **8**: 231-234.
3. Lipshutz BH, Ghorai S and Abela, A. R *et al*. TPGS-750-M: A Second-Generation Amphiphile for Metal-Catalyzed Cross-Couplings in Water at Room Temperature. *J Org Chem* 2011; **76**: 4379-4391.
4. Yang Y, Meng X and Zhu B *et al*. A Micellar Catalysis Strategy for Amidation of Alkynyl Bromides: Synthesis of Ynamides in Water. *Eur J Org Chem* 2019; **2019**:1166-1169.
